# Elucidating the multifaceted roles of GPR146 in non-specific orbital inflammation: a concerted analytical approach through the prisms of bioinformatics and machine learning

**DOI:** 10.3389/fmed.2024.1309510

**Published:** 2024-06-05

**Authors:** Zixuan Wu, Ling Li, Tingting Xu, Yi Hu, Xin Peng, Zheyuan Zhang, Xiaolei Yao, Qinghua Peng

**Affiliations:** ^1^Hunan University of Traditional Chinese Medicine, Changsha, Hunan, China; ^2^Dongying People’s Hospital (Dongying Hospital of Shandong Provincial Hospital Group), Dongying, Shandong, China; ^3^Department of Ophthalmology, The First Affiliated Hospital of Hunan University of Traditional Chinese Medicine, Changsha, Hunan, China

**Keywords:** non-specific orbital inflammation (NSOI), GPR146, LASSO regression, SVM-RFE, autoimmune inflammatory disorder

## Abstract

**Background:**

Non-specific Orbital Inflammation (NSOI) is a chronic idiopathic condition marked by extensive polymorphic lymphoid infiltration in the orbital area. The integration of metabolic and immune pathways suggests potential therapeutic roles for C-peptide and G protein-coupled receptor 146 (GPR146) in diabetes and its sequelae. However, the specific mechanisms through which GPR146 modulates immune responses remain poorly understood. Furthermore, the utility of GPR146 as a diagnostic or prognostic marker for NSOI has not been conclusively demonstrated.

**Methods:**

We adopted a comprehensive analytical strategy, merging differentially expressed genes (DEGs) from the Gene Expression Omnibus (GEO) datasets GSE58331 and GSE105149 with immune-related genes from the ImmPort database. Our methodology combined LASSO regression and support vector machine-recursive feature elimination (SVM-RFE) for feature selection, followed by Gene Set Enrichment Analysis (GSEA) and Gene Set Variation Analysis (GSVA) to explore gene sets co-expressed with GPR146, identifying a significant enrichment in immune-related pathways. The tumor microenvironment’s immune composition was quantified using the CIBERSORT algorithm and the ESTIMATE method, which confirmed a positive correlation between GPR146 expression and immune cell infiltration. Validation of GPR146 expression was performed using the GSE58331 dataset.

**Results:**

Analysis identified 113 DEGs associated with GPR146, with a significant subset showing distinct expression patterns. Using LASSO and SVM-RFE, we pinpointed 15 key hub genes. Functionally, these genes and GPR146 were predominantly linked to receptor ligand activity, immune receptor activity, and cytokine-mediated signaling. Specific immune cells, such as memory B cells, M2 macrophages, resting mast cells, monocytes, activated NK cells, plasma cells, and CD8+ T cells, were positively associated with GPR146 expression. In contrast, M0 macrophages, naive B cells, M1 macrophages, activated mast cells, activated memory CD4+ T cells, naive CD4+ T cells, and gamma delta T cells showed inverse correlations. Notably, our findings underscore the potential diagnostic relevance of GPR146 in distinguishing NSOI.

**Conclusion:**

Our study elucidates the immunological signatures associated with GPR146 in the context of NSOI, highlighting its prognostic and diagnostic potential. These insights pave the way for GPR146 to be a novel biomarker for monitoring the progression of NSOI, providing a foundation for future therapeutic strategies targeting immune-metabolic pathways.

## 1 Introduction

Non-Specific Orbital Inflammation (NSOI) epitomizes a complex, benign inflammatory disorder localized to the orbital compartment, absent of discernable systemic or local etiological factors. Contributing to 6–16% of all ocular pathologies and accounting for 11% of neoplastic phenomena within the orbital realm, NSOI disproportionately impacts middle-aged individuals and manifests a marked female predominance (1, 2). Despite its epidemiological significance, the pathophysiological underpinnings of NSOI remain substantially elusive. Existing literature tentatively links NSOI with diverse medical conditions such as Streptococcal pharyngitis, viral upper respiratory infections, and a spectrum of autoimmune disorders including rheumatologic conditions, multifocal fibrosis, and Crohn’s disease (3, 4). The clinical phenotype of NSOI is notably heterogeneous, with presentations ranging from dacryoadenitis and lacrimal gland inflammation to myositis involving singular or multiple extraocular muscles (EOMs), alongside other non-canonical manifestations (5). Although systemic corticosteroids serve as the cornerstone of current therapeutic regimens, their protracted utilization is marred by a litany of documented adverse sequelae (6). Moreover, the specter of disease recurrence looms large, exceeding a staggering 50% even following ostensibly successful corticosteroid interventions (7). Given this backdrop, the imperative to delineate the molecular machinations underpinning NSOI becomes increasingly urgent. Such elucidation holds the promise of paving the way for innovative therapeutic interventions, with the objective of reducing recurrence rates and thereby enhancing patient prognosis.

In 2013, GPR146 was identified through GWAS as a significant gene involved in lipid metabolism (8). The common SNP rs1997243, located near the GPR146 locus, occurs in 14% of the population and is associated with elevated total cholesterol levels in plasma (8). Transcriptomic data indicate that GPR146 is predominantly expressed in white adipose tissue, playing a role in adipocyte differentiation (9, 10). Interestingly, GPR146 has also been recognized as an antiviral modulator. Research led by Huang et al. highlighted that GPR146 is upregulated by interferon via a STAT1-dependent pathway. However, surprisingly, GPR146 knockout in murine models did not alter viral susceptibility, a phenomenon possibly explained by the suppression of endogenous GPR146 expression through an IRF3/HES1 signaling pathway in virus-stimulated cells (11). This suggests a complex and potentially reciprocal interaction between GPR146 and HES1 signaling, with implications for therapeutic strategies against viral infections (12). Although GPR146 has been extensively studied in the context of lipid-related disorders such as hypercholesterolemia and atherosclerosis, its role in other immune-inflammatory conditions is less understood. A deeper comprehension of the regulatory networks linking GPR146 with other chemokine systems is crucial for the design of targeted therapies not only for metabolic and viral ailments but also for inflammatory diseases such as those affecting the retina. Thus, elucidating these intricate interactions remains a critical area for future research and therapeutic development.

**FIGURE 1:**
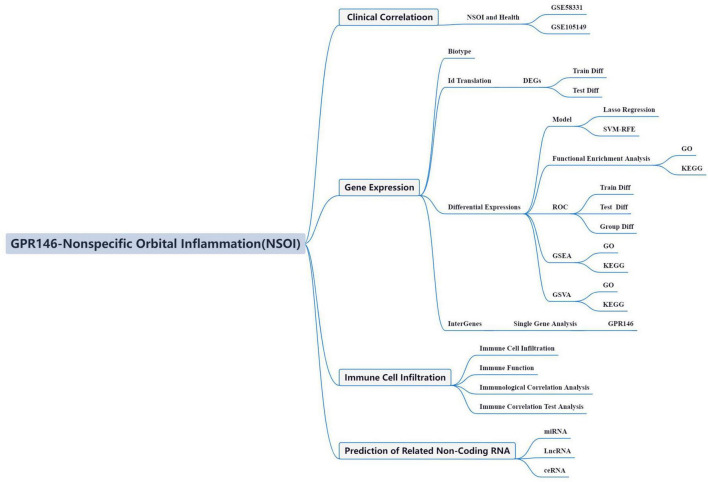
Framework.

In the dynamic field of oncological research, recent paradigms have spotlighted a unique metabolic phenotype characteristic of neoplastic cells that fundamentally reconfigures the immune microenvironment. This environment, a complex assembly of cellular entities supported by a compromised vascular system, hinders efficient nutrient and oxygen distribution (13). These insights have intensified investigations into non-tumorigenic immune infiltration within this microenvironment, a burgeoning area of scientific inquiry. Seminal work by Sharma et al. suggests that the immunosuppressive milieu, teeming with diverse immune cells, orchestrates extracellular mechanisms that confer resistance to immunotherapeutic strategies (14). However, the intricate relationship between this immunosuppressive architecture and NSOI system remains poorly understood, highlighting a critical gap in our mechanistic understanding and the development of tailored therapeutic interventions. Advances in high-throughput technologies and bioinformatics have transformed our ability to identify biomarkers and therapeutic targets. Modern bioinformatics now leverages sophisticated approaches such as Weighted Gene Co-expression Network Analysis (WGCNA) and machine learning algorithms—LASSO, SVM-RFE, and random forest (RF)—to pinpoint potential disease targets and refine diagnostic models. These tools enable comprehensive gene functional network analyses across different disease models, shedding light on complex molecular mechanisms (15). Utilizing the rich transcriptome sequencing data and clinical annotations of the NSOI Initiative, this study aims to delve into the transcriptional alterations and associated molecular pathways of NSOI. These bioinformatics explorations are intended to provide deep insights into the pathophysiology and underlying mechanisms of NSOI. Despite these advancements, the specific role of bioinformatics in exploring NSOI within its own context has not been previously addressed (16, 17). Thus, this study seeks to investigate NSOI-related datasets from the GEO, focusing on biomarkers and pathways integral to NSOI, as illustrated in Figure 1. This approach promises to open new avenues for understanding and potentially improving the management of this complex condition.

## 2 Materials and methods

We used the approaches proposed by Wu et al. (18).

### 2.1 Source of transcriptional profiling data

The mRNA expression datasets pertinent to NSOI were acquired from the GEO repository. Specifically, this investigation harnessed the datasets GSE58331 and GSE105149, underpinned by the GPL570-55999 platform. GSE58331 functioned as the training cohort, while GSE105149 constituted the testing group.

Gene Expression Omnibus was searched for mRNA expression. Series: GSE58331 and GSE105149. Platform: GPL570-55999. GSE58331 and GSE105149 were used as the trian and test groups, respectively. Strategy for searching (“eye” [MeSH] mRNA [All Fields] and normal) AND (“Homo sapiens” [Organism] AND “Non-coding RNA profiling by array” [Filter]). Specifically, this investigation harnessed the datasets GSE58331 and GSE105149, underpinned by the GPL570-55999 platform. GSE58331 functioned as the training cohort, while GSE105149 constituted the testing group (Table 1).

**TABLE 1:** The clinical characteristics of patients.

GSE58331		GSE105149	
**Variables**	**Number of samples**	**Variables**	**Number of samples**
**Gender**		**Gender**	
Male/female	19/56	Male/female	9/18
**Diagnosis**		**Diagnosis**	
NSOI/normal	75/29	NSOI/normal	0/27
**Tissue**		**Tissue**	
Anterior Orbit/lacrimal gland	33/42	Anterior Orbit/lacrimal gland	0/27

### 2.2 Transcriptomic data refinement and pre-processing

The acquired probe-centric expression matrices were transmuted into gene-level expression matrices, capitalizing on the auxiliary probe annotation documentation. In instances where multiple probes corresponded to a solitary gene, an arithmetic mean of these probe values was computed to typify the definitive expression metric of the respective gene. Subsequent to the standardization of the datasets, batch effect normalization was executed employing the SVA package. The efficacy of batch effect rectification was gauged through principal component analysis (PCA). Differential expression analyses between NSOI and control groups were conducted utilizing the Linear Models for Microarray Data (limma) package. Criteria for defining DEGs were set at an absolute log fold change (| log FC|) greater than 1 and an adjusted *p*-value less than 0.05, with the aim of isolating DEGs in NSOI cases.

### 2.3 Immune landscape characterization

To reveal the complex interactions between GPR146 and immune-related biological functions, we applied network ranking and estimation algorithms to perform in-depth analysis of immune microenvironment features in individual samples through the limma, GSVA, GSEABase, ggpubr, reshape2 packages. This sophisticated computational approach enabled us to precisely dissect the distribution and differential representation of 22 immune cell subsets across NSOI and control specimens. Statistical validation of these distinctions was achieved using the Wilcoxon rank-sum test, providing robust evidence of differential immunocyte composition. This methodological rigor enhances our understanding of GPR146’s role within the immune landscape, potentially illuminating its influence on immune response mechanisms and its broader implications in disease pathology.

### 2.4 Predictive modeling and computational learning

In this study, we refined our search for key genes by intersecting the differential genes identified through various algorithms using the VNN package, setting the stage for model construction. To build a predictive model noted for its precision and reliability, we employed the glmnet package to implement Lasso regression, enhanced by rigorous cross-validation. This approach effectively minimized overfitting and improved the model’s accuracy across complex biological datasets. Further validation was achieved using the SVM-RFE algorithm through the e1071 package, meticulously crafting a robust machine learning model. Cross-validation was essential in evaluating the model’s error rates and enhancing its precision, thus bolstering its robustness and dependability. Additionally, the Random Forest algorithm was utilized for its ensemble learning capability, generating numerous decision trees to amalgamate outcomes, which reduced overfitting and improved generalization. A distinctive feature of this method—random selection of features and bootstrap sampling—increased diversity among decision trees, enhancing overall model accuracy (19, 20). By employing the randomForest and ggplot2 packages, we concentrated on analyzing differentially expressed genes to identify crucial genes for accurate disease classification. In the final phase, we evaluated the significance of these genes using an integrated approach combining insights from Lasso regression, Random Forest, and SVM models, providing a nuanced view of their roles in disease pathology. These genes, validated through comprehensive methodologies, are set for further exploratory studies. The area under the curve (AUC) from the receiver operating characteristic (ROC) curve is an essential metric that plots the true positive rate against the false positive rate at various thresholds. An AUC of 1.0 signifies an ideal diagnostic test, while an AUC close to 0.5 suggests non-discriminatory power equivalent to random chance. This metric is invaluable for evaluating the diagnostic accuracy of medical tests and the predictive reliability of models. Utilizing the R pROC package, we integrated and analyzed the dataset combining NSOI outcomes with pivotal genes to assess their predictive accuracy, further employing the dataset GSE58331 for validation. Through this ROC analysis, we established a comprehensive methodological framework to gauge the diagnostic performance of these biomarkers, enhancing our understanding of their clinical utility.

### 2.5 Functional annotation via GO and KEGG pathway analyses

The biological pathways associated with the DEGs were then examined using Gene Ontology (GO). Biological processes (BP), molecular functions (MF), and cellular components (CC) controlled by the differentially expressed ATG genes were further investigated using R software, clusterProfiler, org.Hs.eg.db, enrichplot, and ggplot2 package based on KEGG data.

### 2.6 Integrated enrichment analysis using GSEA and GSVA

Global gene-set enrichment analyses, encompassing GSEA and GSVA, were utilized to identify functionally coherent gene sets and signaling cascades differentially active across the studied samples. Enrichment scores and accompanying visual representations were generated to discern dynamic activities and pathways across various risk stratifications. R was deployed to investigate the influence of differential GPR146 expression on BP, MF, CC, and implicated pathways form GSEA database.^1^

### 2.7 Biomarker-immune infiltrate correlation analyses

The core tenet of immune infiltration analysis lies in the precise quantification of the prevalence and functional status of various immune cell types, such as T cells, macrophages, and dendritic cells, within the immune microenvironment. This evaluation is deeply intertwined with the expression profiles of specific biomarkers, through the use of sophisticated statistical techniques and computational models. These approaches enable researchers to delineate the correlations between distinct immune cells and particular biomarkers, thereby illuminating their roles in the initiation and progression of diseases. In examining the role of GPR146, an extensive immune infiltration analysis was carried out employing a suite of bioinformatics tools. Differential expression analysis was conducted using the limma package, while gene set enrichment analysis utilized GSVA and GSEABase. Additionally, the ggpub and reshape2 packages were instrumental for data visualization and restructuring, respectively. Spearman/s rank correlation coefficient was applied to reveal the intricate associations between diagnostic biomarkers and the patterns of immune cell infiltration, significantly deepening our comprehension of their interactive dynamics within the immune microenvironment.

### 2.8 Dissecting miRNA and lncRNA cross-talk in NSOI

Non-coding RNAs, notably miRNAs and lncRNAs, serve as pivotal modulators of gene expression. While miRNAs principally function through post-transcriptional regulation either by promoting or inhibiting mRNA degradation and translation, lncRNAs engage in multifaceted regulatory capacities, including chromatin remodeling, transcriptional activation, and interference mechanisms. Recent discoveries underscore the intricate interplay between miRNAs and lncRNAs, revealing ceRNA networks. Accordingly, this study aims to unearth common regulatory axes and developmental trajectories involving miRNAs and lncRNAs within the NSOI context.

### 2.9 Construction of integrated mRNA-miRNA-lncRNA regulatory networks

Empirically validated target gene information for the common miRNAs and lncRNAs was retrieved from miRTarbase and PrognoScan databases. An intersecting regulatory network, encapsulating mRNA-miRNA-lncRNA interplay and their shared targets in NSOI, was assembled and visualized using Cytoscape software.

### 2.10 Statistical considerations

Statistical assessment of gene expression disparities between the distinct cohorts was executed via the ggpubr package in R (version 4.3.1). For data adhering to a normal distribution, two-sample independent *t*-tests were utilized; alternatively, the Wilcoxon rank-sum test was applied for non-normally distributed data. A *p*-value threshold of less than 0.05 was deemed statistically significant for all tests.

## 3 Results

### 3.1 Identification of DEGs and principal component analysis

**FIGURE 2:**
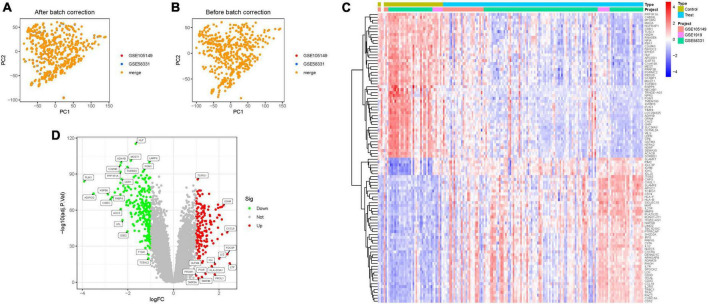
Principal component analysis. **(A,B)** Analysis of PCA. **(C)** Heatmap. **(D)** Volcano map.

Through the integration of GSE58331 and GSE105149 datasets, and subsequent batch match evidence synthesis, we corroborated the effective stratification of patients into distinct risk cohorts via Principal Component Analysis (PCA) (Figures 2A, B). Of the identified 314 Differential Expressed Genes (DEGs), a subset demonstrated significant variations. Furthermore, distinct clustering of several genes was observed in the treatment and control groups, respectively; Treatment group: PPP1R1A, CAB39L, MTURN, MAOA2, NGFRAP1, CDR1, etc., Control group: ITGB2, CAPG, CHI3L1, SLAMF8, APOC1, TCIRG1, etc. (Figure 2C). Notably, several DEGs such as TCIRG1, IGHM, CXCL9, PROM1, PIGR, HLA-DQA1, exhibited significant up-regulation, while others including HLF, ADH1B, MGST1, LARP6, PGM1, C2orf40, TGFBR3, manifested significant down-regulation (Figure 2D; Supplementary Tables 1, 2).

### 3.2 Model construction

**FIGURE 3:**
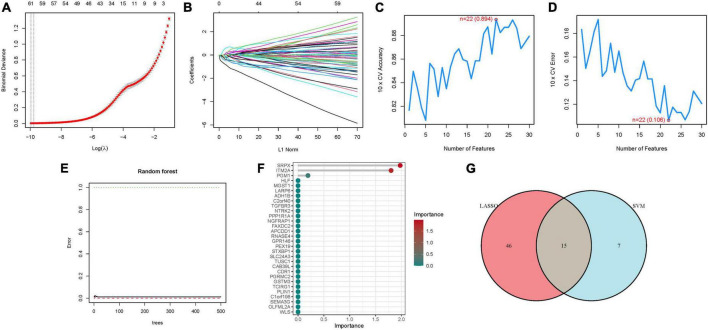
The development of the signature. **(A)** Regression of the NSOI-related genes using LASSO. **(B)** Cross-validation is used in the LASSO regression to fine-tune parameter selection. **(C,D)** Accuracy and error of this model. **(E,F)** Random forest analysis. **(G)** Venn.

Utilizing LASSO, Cox regression analysis, and optimum value, we established a gene signature (Figures 3A, B). Subsequently, SVM-RFE was employed to develop a machine-learning model to ascertain the accuracy and reliability of the constructed model. The derived model exhibited an accuracy of 0.894, and an error rate of 0.106 (Figures 3C, D). Essential genes including SRPX, ITM2A, PGM1, HLF, were identified through Random Forest analysis (Figures 3E, F). An attempt to amalgamate key genes from these three algorithms for model construction revealed that only models constructed through LASSO and SVM-RFE demonstrated optimal stability in key gene construction. Consequently, a total of 15 hub genes were elucidated (Figure 3G; Supplementary Table 3).

### 3.3 Visualization and identification of DEGs

**FIGURE 4:**
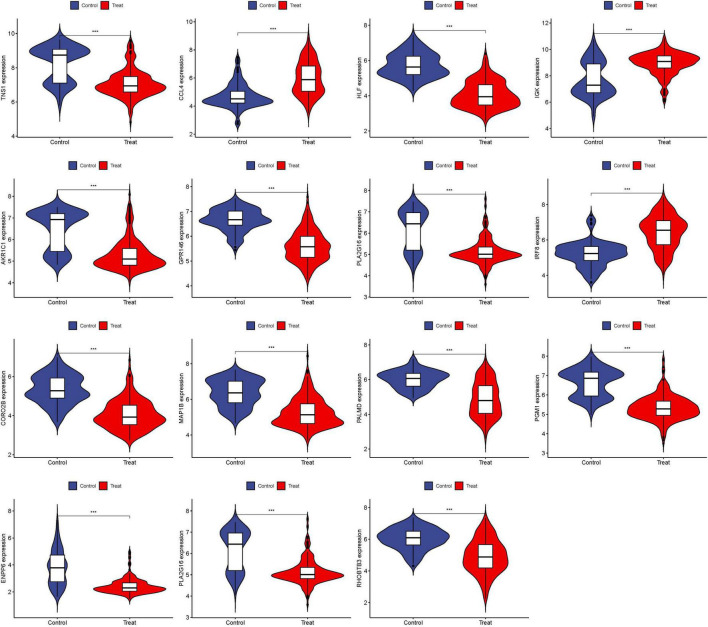
Expression of 15 hub genes in NSOI group and normal sample group, respectively. ***P < 0.001.

**FIGURE 5:**
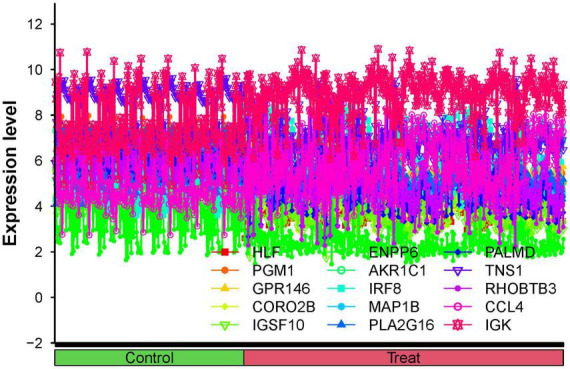
All hub genes are co-expressed in the same line plot.

**FIGURE 6:**
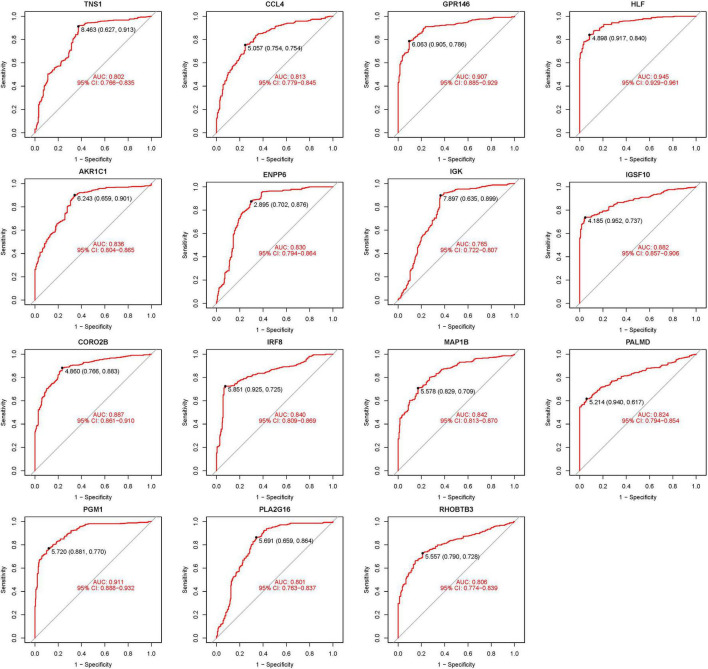
Receiver operating characteristic (ROC) of 15 hub genes.

The 15 identified hub genes were visualized within both the NSOI group and the normal sample group (Figure 4), and were further represented in a comprehensive graph for comparative visualization (Figure 5). The verification of these 15 hub genes was accomplished through the analysis of the Receiver Operating Characteristic (ROC) of each gene, demonstrating high accuracy for each. Specifically, the following Area Under Curve (AUC) values were observed: HLF (0.945), PGM1 (0.911), GPR146 (0.907), IRF8 (0.840), TNS1 (0.802), PLA2G16 (0.801), PALMD (0.824), CCL4 (0.813), IGK (0.765), CORO2B (0.887), IGSF10 (0.882), AKR1C1 (0.836), ENPP6 (0.830), MAP1B (0.842), RHOBTB3 (0.806) (Figure 6).

### 3.4 Validation of hub genes

**FIGURE 7:**
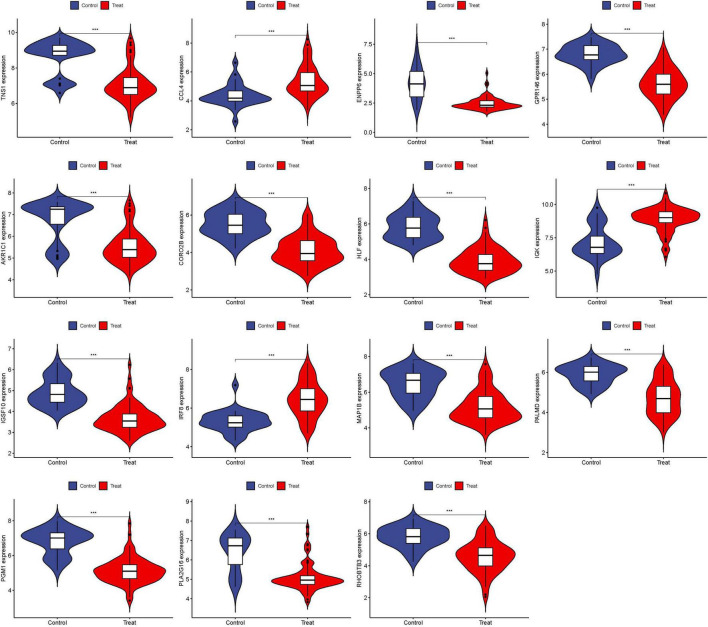
Expression of 15 hub genes in GSE58331 analysis. ***P < 0.001.

**FIGURE 8:**
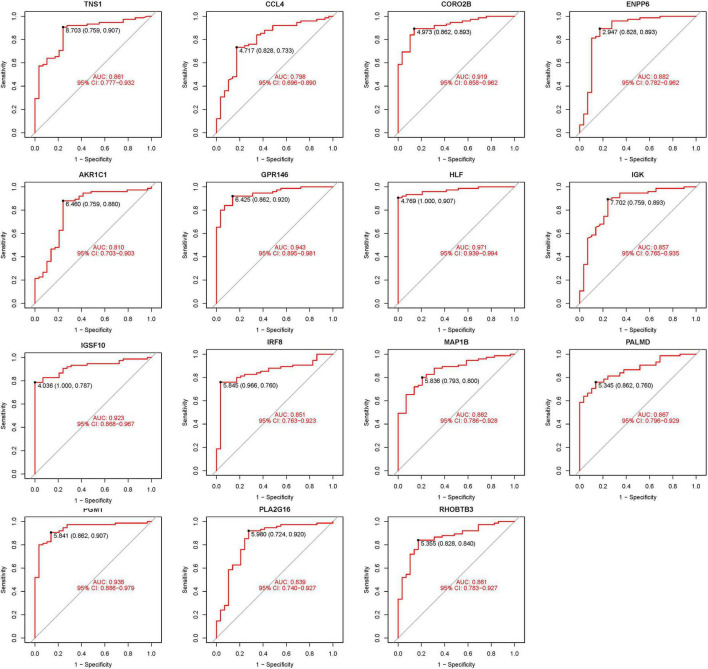
Receiver operating characteristic (ROC) of 15 hub genes.

To enhance the confidence and predictive accuracy of our model regarding the hub genes, GSE58331 was employed for validation. Intriguingly, a significant difference in these DEGs was manifested in the GSE58331 analysis (Figure 7). In the validation of the 15 hub genes within GSE58331, the ROC analysis of these genes showcased their high accuracy, evidenced by the AUC values: HLF (0.971), PGM1 (0.938), GPR146 (0.943), IRF8 (0.851), TNS1 (0.861), PLA2G16 (0.839), PALMD (0.867), CCL4 (0.798), IGK (0.857), CORO2B (0.919), IGSF10 (0.923), AKR1C1 (0.810), ENPP6 (0.882), MAP1B (0.862), RHOBTB3 (0.861). These outcomes further corroborate the high reliability and accuracy of our devised model (Figure 8).

### 3.5 DEG identification of GPR146

**FIGURE 9:**
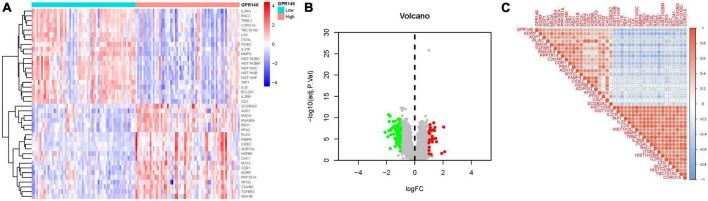
Differentially expressed gene (DEG) Identification of GPR146. **(A)** Heatmap. **(B)** Volcano map. **(C)** Correlation matrix diagram.

Through differential analysis targeting individual genes, 113 DEGs were identified. Among them, certain DEGs exhibited significant variance. Additionally, distinct gene clusters were observed in both high and low groups; High: AQP1, MAOA, RNASE4, PBX1, PFN2, PLIN1, FABP4, CIDEC, etc., Low: IL2RG, RAC2, TRBC1, CORO1A, TBC1D10C, LCK, ITGAL, ITGB2, etc. (Figures 9A, B). Furthermore, we generated a correlation matrix plot associated with GPR146 (Figure 9C; Supplementary Table 4).

### 3.6 Enrichment analysis of DEGs of GPR146

**FIGURE 10:**
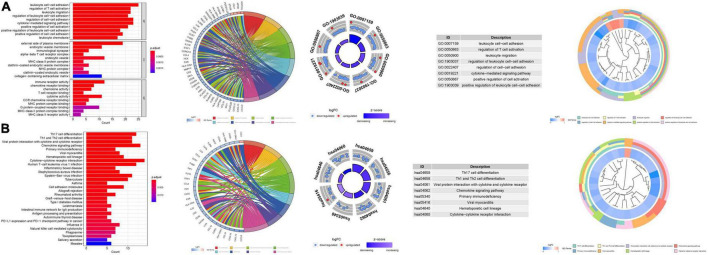
For PMGs, GO, and KEGG analyses were performed. **(A)** The GO circle illustrates the barplot, chord, circos, and cluster of the selected gene’s logFC. **(B)** The KEGG barplot, chord, circos, and cluster illustrates the scatter map of the logFC of the indicated gene.

Gene ontology enrichment analysis disclosed 1120 core targets, encompassing BP, MF, and CC. Predominantly, MF was associated with receptor ligand activity (GO:0048018), immune receptor activity (GO:0140375), and cytokine activity (GO:0005125). CC was chiefly implicated in the external side of the plasma membrane (GO:0009897), endocytic vesicle (GO:0030139), and collagen-containing extracellular matrix (GO:0062023). BP was principally involved in leukocyte cell-cell adhesion (GO:0007159), cytokine-mediated signaling pathway (GO:0019221), and regulation of cell-cell adhesion (GO:0022407). KEGG enrichment analysis illuminated that the over-expressed genes were predominantly engaged in Cytokine-cytokine receptor interaction (hsa04060), Chemokine signaling pathway (hsa04062), and Th17 cell differentiation (hsa04659) (Figure 10; Supplementary Tables 5a, b).

### 3.7 GSEA

**FIGURE 11:**
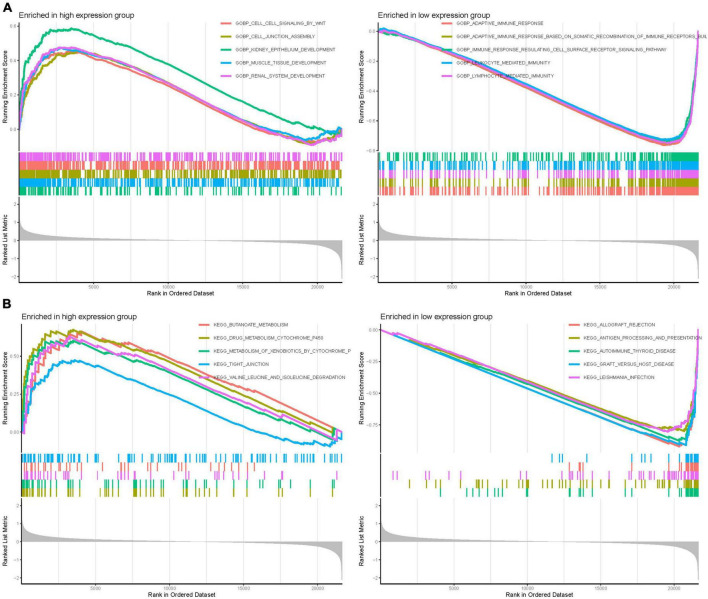
Gene set enrichment (GSEA) of Analysis in GPR146. **(A)** GO. **(B)** KEGG.

Gene Set Enrichment Analysis was employed to discern functional alterations across the DEGs of GPR146. In the high-expression group of GO analysis, functional enrichment was primarily observed in BP such as kidney epithelium development, muscle tissue development, and renal system development. Conversely, in the low-expression group, the enrichment focused on adaptive immune response, adaptive immune response based on somatic recombination of immune receptors, and lymphocyte-mediated immunity (Figure 11A). Regarding KEGG analysis, the high-expression group showcased enrichment in pathways like butanoate metabolism, drug metabolism cytochrome p450, and valine, leucine, and isoleucine degradation. The low-expression group highlighted involvement in allograft rejection, autoimmune thyroid disease, and graft-vs.-host disease (Figure 11B; Supplementary Table 6).

### 3.8 GSVA of analysis

**FIGURE 12:**
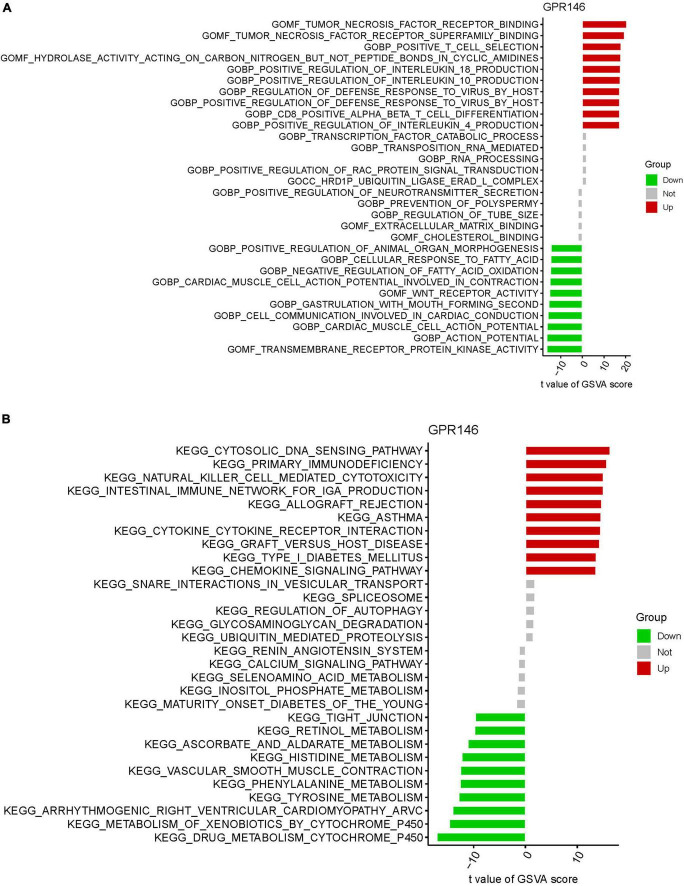
Gene set variation (GSVA) of Analysis in GPR146. **(A)** GO. **(B)** KEGG.

Gene Set Variation Analysis identified functional alterations in the DEGs of GPR146. GO analysis revealed significant involvement in Molecular Functions (MF) such as tumor necrosis factor receptor binding, tumor necrosis factor receptor superfamily binding, alongside Biological Processes (BP) like positive T cell selection and positive regulation of interleukin 18 production, and Cellular Components (CC) like the HRD1P ubiquitin ligase ERAD-L complex (Figure 12A). KEGG analysis indicated enrichment in the cytosolic DNA sensing pathway, primary immunodeficiency, natural killer cell-mediated cytotoxicity, and intestinal immune network for IgA production (Figure 12B; Supplementary Table 7).

### 3.9 Characterization of the immune landscape

**FIGURE 13:**
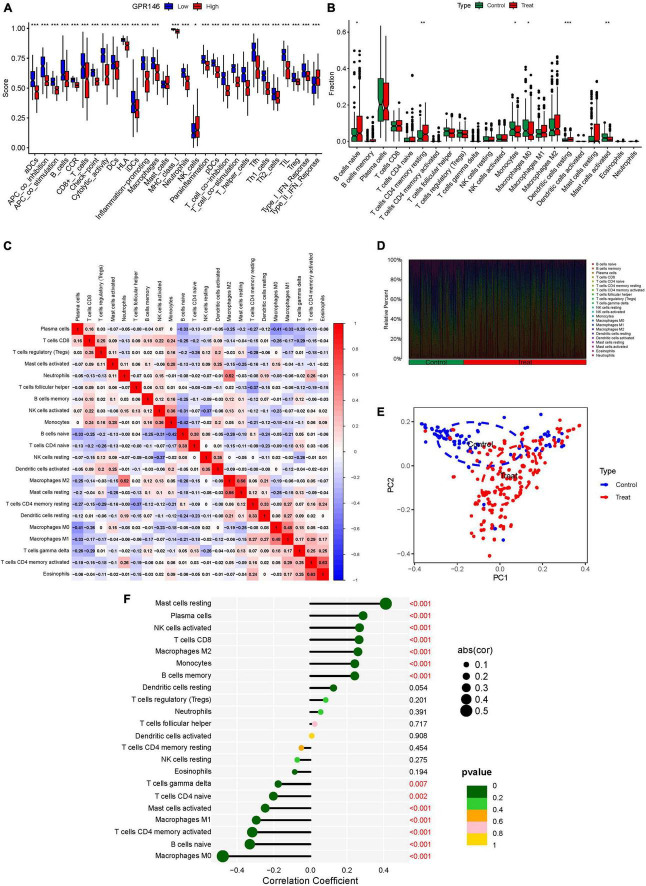
Immune Landscape Characterization. **(A)** Expression of immune function. **(B)** Expression of immune cells **(C)** Correlation rectangle plot. **(D)** Heatmap. **(E)** PCA analysis. **(F)** The expression patterns of Correlation Coefficient. ns, not significant; *P < 0.05; **P < 0.01; ***P < 0.001.

**FIGURE 14:**
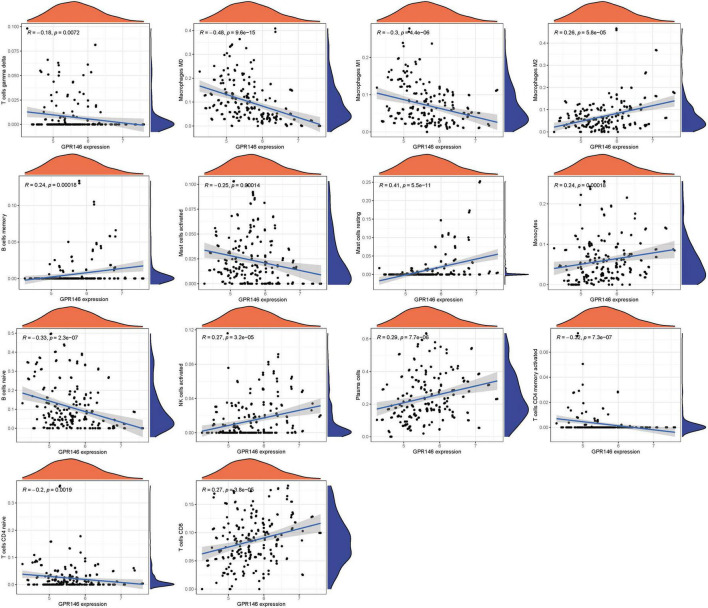
Immune infiltration analyses.

Variance in the infiltration of aDCs, APC co-inhibition, APC co-stimulation, B cells, CCR, and CD8+ T cells was significant between low and high-risk groups, whereas Mast cells showed no significant variance (*P* > 0.05) (Figure 13A). In immune cell characterization, B cells naive, T cells CD4 memory resting, and Dendritic cells resting were highly expressed in the treatment group, while Monocytes, Macrophages M0, and Mast cells activated were predominant in the Control group (Figure 13B). Further, we constructed an immune infiltration correlation rectangle plot and heatmap, facilitating successful immune-based patient categorization through PCA analysis (Figures 13C–E). A Lollipop plot was generated to exhibit the expression patterns of Correlation Coefficients, with Mast cells resting, Plasma cells, NK cells activated, T cells CD8, and Macrophages M2 demonstrating the highest correlation (Figure 13F). Several immune cells showed a positive association with GPR146, while others were negatively linked (Figure 14; Supplementary Table 7).

### 3.10 Construction of miRNAs-lncRNAs shared genes network and identification of common RNAs

**FIGURE 15:**
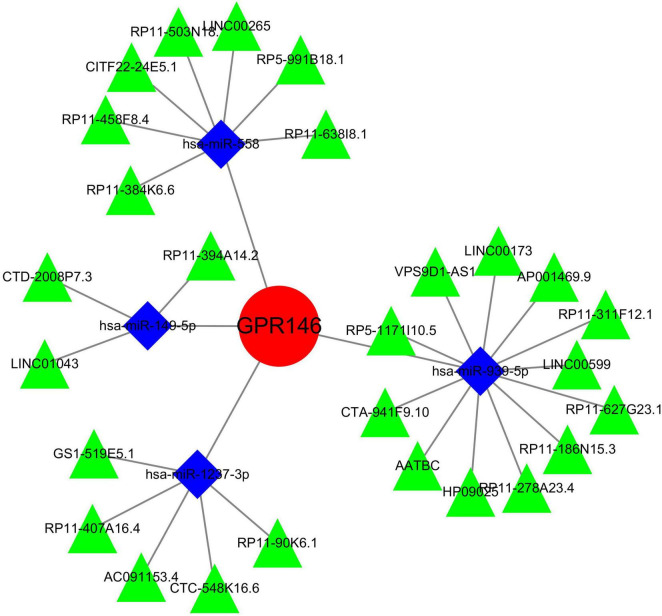
miRNAs-LncRNAs shared genes network. Red circles are mrnas, blue quadrangles are miRNAs, and green triangles are lncRNAs.

An exhaustive search across three databases yielded 9 miRNAs and 27 lncRNAs associated with NSOI (Supplementary Tables 7a, b). The intersecting network of miRNAs-lncRNAs-genes was established by integrating shared genes identified through Lasso regression and SVM-RFE. Consequently, the resulting network comprised 27 lncRNAs (e.g., LINC01043, AATBC, GS1-519E5.1) and 9 miRNAs (e.g., hsa-miR-4269, hsa-miR-1237-3p, hsa-miR-149-5p) (Figure 15; Supplementary Table 8).

## 4 Discussion

Non-specific Orbital Inflammation presents a perplexing ocular anomaly characterized by unilateral orbital edema lacking identifiable viral or systemic origins. Notably, exacerbations in NSOI may stem from optic nerve dysfunction, yet the molecular underpinnings of this disorder remain largely elusive (21). Mounting evidence implicates gene expression modulation as pivotal in NSOI pathophysiology, with the orphan G protein-coupled receptor gene GPR146 emerging as a prominent regulator of plasma cholesterol levels (22). Studies in murine models have demonstrated that GPR146 deficiency confers significant protection against atherosclerosis, reducing lesion areas by up to 90% through an LDL receptor-independent mechanism (22). Yu et al. (23) recently proposed GPR146 as a susceptibility gene influencing plasma cholesterol metabolism, thereby impacting the development of Atherosclerosis and Homozygous Familial Hypercholesterolemia (23). Specifically, GPR146 knockdown impedes VLDL secretion, mitigating aortic atherosclerotic lesions independently of LDLR activity (24). Critical to GPR146-mediated cholesterol homeostasis is the ERK1/2/SREBP2 signaling axis, offering protection against atherosclerosis and hypercholesterolemia in LDLR-deficient models (25). Moreover, GPR146 is implicated in modulating antitumoral immune responses, influencing neoplastic growth (26). The intricate interplay between GPR146 and the immune milieu holds profound implications for various pathological conditions, disease trajectories, therapeutic responses, and prognostic outcomes (27). While numerous risk markers have been proposed in ocular pathologies, many await robust peer review and large-scale validation (28). A comprehensive understanding of GPR146’s involvement in NSOI could unveil the disorder’s molecular intricacies and pioneer novel therapeutic avenues for this enigmatic condition.

Within the framework of NSOI, our multidimensional computational pipeline discerned 113 DEGs involving GPR146. Utilizing a synergistic analytical approach that amalgamated Lasso regression and SVM-RFE, we pinpointed a specific cadre of key DEGs intimately associated with NSOI pathophysiology. Subsequent stratification through cross-over analyses revealed a signature set of 15 hub genes—specifically, HLF, PGM1, GPR146, IRF8, TNS1, PLA2G16, PALMD, CCL4, IGK, CORO2B, IGSF10, AKR1C1, ENPP6, MAP1B, and RHOBTB3. Validation against independent external datasets corroborated their diagnostic utility, firmly implicating them within the convoluted mechanistic nexus underlying NSOI. It is imperative to underscore that, despite these significant strides, the current dataset stops short of definitively attributing these genes to any particular transcriptional regulators pivotal for NSOI modulation. Nevertheless, among the delineated hub genes, GPR146 stands as an exceptionally compelling candidate. It has garnered considerable attention owing to its well-documented involvement in inflammation and immune responses. The unmasking of these hub genes furnishes viable platforms for subsequent investigative ventures. However, a more intricate comprehension of their regulatory architectures remains a sine qua non for decoding the esoteric molecular mechanisms that orchestrate NSOI.

In a groundbreaking revelation, Rimbert et al. uncover an intriguing association between GPR146 and plasma concentrations of CRP, a cardinal biomarker for systemic inflammation. This discovery is particularly salient given the pivotal role that inflammation is known to play in atherogenesis (29). Postulated to be universally modulated by pathophysiological states, secretory epithelia-including those in ocular tissues-are implicated in various disease processes. Intriguingly, the abrogation of GPR146 functionality is not correlated with elevations in hepatic enzymes, often employed as diagnostic indices for hepatic steatosis or non-alcoholic fatty liver disease. Such data collectively point toward the prospective utility of GPR146 antagonists as potentially superior therapeutic agents compared to existing alternatives (26). In the realm of biomedical research, the relationship between GPR146 and NSOI has garnered substantial interest. GPR146, a G protein-coupled receptor of potential biological significance, plays a pivotal role in various physiological and pathological processes. However, the specific contributions of GPR146 to the pathogenesis of NSOI and its roles within this disease have not been thoroughly investigated. NSOI is a complex inflammatory orbital disorder with an incompletely understood pathogenic mechanism. Increasing evidence suggests that inflammatory mediators and immune modulators play crucial roles in the progression of NSOI. Given the potential of GPR146 as an immune modulatory molecule, its involvement in regulating inflammatory and immune responses may be significant. Consequently, in-depth studies into the role of GPR146 in NSOI could enhance our understanding of the disease’s pathophysiology. Future research employing cellular and animal models could probe the specific mechanisms by which GPR146 influences the onset and progression of NSOI. Furthermore, analyzing the expression and functional changes of GPR146, in conjunction with clinical case studies and trials, may reveal its viability as a therapeutic target, offering new avenues and strategies for the treatment of NSOI. In an endeavor to decipher the complex regulatory matrix, understanding the orchestrated interactions among these key elements could furnish invaluable insights into modulating proinflammatory responses, not just in retinal tissues but also in extra-retinal domains. Lending empirical weight to these biological complexities, our inquiry substantiates the pivotal role of DEGs, with particular emphasis on GPR146, in the intricate pathophysiological fabric of NSOI. Evidence derived from the GSE105149 dataset intimates that a GPR146-associated phenotypic trait could serve as a potent prognostic marker, elevating the gene’s clinical significance.

The immune landscape plays a pivotal role in the onset and progression of NSOI. Notably, distinct differences in immune cell infiltration were observed in risk-associated profiles within the GPR146 cohort. In the multifaceted realm of NSOI, burgeoning data are beginning to upend the traditional paradigm that ascribes heightened immune reactivity solely to CD4 T-cell activities. Rather, a more intricate interplay involving a diverse repertoire of preexisting T-regulatory cells, together with a complex equilibrium of both proinflammatory and regulatory cytokines, appears to be at play (30). This finely balanced immunological landscape serves as a precursor for a perturbed immune reconstitution, rendering the host susceptible to a plethora of opportunistic infections, whether extant, latent, or hitherto contained (31). Diseases such as Tuberculosis, Cytomegalovirus infections, Progressive Multifocal Leukoencephalopathy, Kaposi’s Sarcoma, and various autoimmune disorders exhibit the potential to either exacerbate the condition or elude conventional diagnostic modalities. Significantly, Cytomegalovirus retinitis emerges as the predominant opportunistic infection correlated with Immunological Recovery Uveitis (21, 32). In a therapeutic context, advances targeting the elevation of intracellular cAMP levels present a promising frontier in the quest to mitigate chronic inflammatory pathologies. Specifically, small-molecule PDE4 inhibitors, which forestall cAMP degradation, have manifested robust efficacy across a broad spectrum of inflammatory diseases, including but not limited to Inflammatory Bowel Disease, Atopic Dermatitis, and Rheumatoid Arthritis (33, 34). This elucidates not only the intricate etiological factors underlying NSOI but also extends a critical platform for the exploration of innovative therapeutic strategies.

In a logical extension of our preceding research endeavors, we rigorously assessed the expression dynamics of GPR146 within the context of the immunological microenvironment. Utilizing a Lollipop plot, we visualized the nuanced correlation coefficient landscape, identifying a spectrum of immune cells that exhibited marked correlation with GPR146 (Figure 13F). Notably, Mast cells (resting), Plasma cells, NK cells (activated), T cells CD8, and Macrophages M2 emerged as the most closely correlated immunological entities. On the flip side, we observed an inverse relationship between GPR146 and several other cell types, namely, Macrophages M0, B cells (naive), Macrophages M1, Mast cells (activated), T cells CD4 (memory activated), T cells CD4 (naive), and T cells gamma delta. This complex tableau of interactions between GPR146 and a diverse assortment of immunological cell types accentuates the integral role of inflammation and immune responses in the intricate pathophysiology of NSOI. These findings not only elucidate the multi-dimensional nature of GPR146’s involvement but also lay the groundwork for the development of targeted therapeutic avenues in the treatment of this enigmatic condition.

Within the relatively uncharted interface of biomarkers and NSOI, this study serves as a groundbreaking addition to a rapidly evolving scientific discourse. Existing scholarship has leveraged bioinformatics to elucidate associations between immunological markers and ocular diseases; however, there has been a striking absence of focused attention on the role of GPR146 in NSOI pathogenesis (35–37). Emblematic contributions to the field include work by Liu et al. (35), who employed Weighted Gene Co-expression Network Analysis to isolate central hub genes in NSOI, and Hu et al., who utilized cutting-edge computational techniques to reveal 11 cornerstone genes in thyroid eye disease, such as ATP6V1A, PTGES3, and PSMD12. Furthermore, Huang et al. conducted a comprehensive bioinformatics scrutiny augmented by *in vivo* validation, identifying six critical genes like CD44 and CDC42 in the realm of diabetic retinopathy. This study represents a significant advancement in the field of NSOI research through the implementation of a novel GPR146 analytical framework, exploring new dimensions previously untouched by existing research. By harnessing a comprehensive dataset of GPR146 extracted from the GEO, this investigation provides a robust platform for intricate data analysis, thereby elevating the robustness and breadth of our methodological approach. This approach not only enriches the understanding of NSOI pathophysiology but also sets a new benchmark for future explorations into this critical area of study. Despite these advancements, the study is not devoid of limitations. While it utilizes refined bioinformatic techniques to process and analyze data, and validates these findings through comparative analysis with other gene expression profiles, the nature of our inquiry remains exploratory. The preliminary results, although promising, underscore the need for further empirical research to validate these findings comprehensively. It is imperative that subsequent studies build on this groundwork with rigorous empirical testing to solidify the theoretical constructs proposed here, and to further illuminate the role of GPR146 in NSOI.

Continued investigations must leverage this foundational work through rigorous empirical validation to consolidate the theoretical frameworks delineated herein and to deepen our understanding of the involvement of GPR146 in NSOI. NSOI comprises a diverse spectrum of inflammatory conditions within the orbit, each exhibiting varying degrees of activity and responsiveness to anti-inflammatory agents. Thus, while the findings presented herein offer initial insights, they necessitate expansion and robust correlation with clinical manifestations in NSOI. To achieve this, future studies should prioritize several key avenues of inquiry. Firstly, comprehensive clinical studies are warranted to elucidate the precise relationship between GPR146 expression levels and the clinical activity of NSOI. Longitudinal analyses, encompassing a diverse patient cohort, should be conducted to discern any temporal patterns in GPR146 expression and disease progression. Moreover, investigations into the impact of anti-inflammatory medications on GPR146 expression dynamics could provide invaluable insights into its functional role within the context of NSOI treatment. Furthermore, mechanistic studies at the cellular and molecular levels are essential to unravel the intricacies of GPR146-mediated immune regulation in NSOI. Employing advanced experimental techniques, such as cell culture models and gene editing technologies, could elucidate the downstream signaling pathways modulated by GPR146 activation and shed light on its interaction with other immune mediators implicated in NSOI pathogenesis. Additionally, translational research efforts should aim to harness the diagnostic and therapeutic potential of GPR146 in NSOI management. Development of GPR146-targeted diagnostic assays, alongside exploration of novel therapeutic modalities targeting GPR146 signaling pathways, could revolutionize clinical management strategies for NSOI.

## 5 Conclusion

In navigating the intricate maze of oncological heterogeneity, our research undertakes a rigorous examination of the pivotal role played by GPR146 within the broader arena of Immune Inflammation, thereby establishing its extensive prognostic ramifications. Utilizing avant-garde predictive modeling techniques, we dissect the transcriptional dynamics of GPR146 with precision, uncovering pronounced disparities in gene expression profiles between NSOI and corresponding normal tissue samples. This analytical foray elevates GPR146 to an essential prognostic linchpin in NSOI, exposing a convoluted tapestry of genetic aberrations—including mutations, duplications, and amplifications-that define this elusive immune-inflammatory disorder. Importantly, our scrutiny reveals a significant correlation between the expression levels of GPR146 and the extent of immune cell infiltration within the immune microenvironment. This finding not only enhances the prognostic precision of GPR146 but also heralds its prospective utility as a sensitive indicator for assessing the efficacy of immunotherapeutic interventions across the diverse landscape of NSOI pathologies.

## Data availability statement

The original contributions presented in the study are publicly available. This data can be found here: Gene Expression Omnibus, https://www.ncbi.nlm.nih.gov/geo/, GSE58331 and GSE105149.

## Author contributions

ZW: Writing – original draft, Data curation, Conceptualization. LL: Writing – original draft, Data curation, Formal analysis, Project administration. TX: Writing – original draft, Data curation, Formal analysis, Project administration. YH: Writing – original draft, Methodology, Data curation. XP: Writing – original draft, Methodology, Data curation. ZZ: Writing – original draft, Methodology, Data curation. XY: Writing – review and editing, Data curation, Conceptualization. QP: Writing – review and editing, Methodology, Data curation.

## Footnotes

1
https://www.gsea-msigdb.org/gsea/index.jsp
