# Supplementary material for: Elucidating the multifaceted roles of GPR146 in non-specific orbital inflammation: a concerted analytical approach through the prisms of bioinformatics and machine learning
Source: Front Med (Lausanne). 2024 Jun 5;11:1309510. doi: 10.3389/fmed.2024.1309510 (PMC11188444; doi:10.3389/fmed.2024.1309510)
Supplement: Supplementary file 1 [file Data_Sheet_1.doc]

**Elucidating the Multifaceted Roles of GPR146 in Nonspecific Orbital Inflammation: A Concerted Analytical Approach through the Prisms of Bioinformatics and Machine Learning**

**Supplementary appendix to the manuscript**

Contents of supplementary appendix

[Appendix 1 3](#__RefHeading___Toc27618)

[The clinical characteristics of patients 3](#__RefHeading___Toc6156)

[Table S1. The clinical characteristics of patients. 3](#__RefHeading___Toc16314)

[Appendix 2 4](#__RefHeading___Toc32413)

[DEGs linked to NSOI 4](#__RefHeading___Toc12511)

[Table S2. 314 DEGs linked to NSOI. 4](#__RefHeading___Toc23369)

[Appendix 3 12](#__RefHeading___Toc11948)

[Table S3a. LASSO genes. 12](#__RefHeading___Toc14863)

[Table S3b. SVM-RFE genes. 12](#__RefHeading___Toc20205)

[Table S3c. InterGenes. 13](#__RefHeading___Toc21556)

[Appendix 4 13](#__RefHeading___Toc32240)

[DEG Identification of GPR146 13](#__RefHeading___Toc1144)

[Table S4. 133 DEG Identification of GPR146 13](#__RefHeading___Toc5212)

[Appendix 5 16](#__RefHeading___Toc11818)

[Table S5a. Analysis of GO. 16](#__RefHeading___Toc10368)

[Table S5b. Analysis of KEGG. 47](#__RefHeading___Toc13746)

[Appendix 6 49](#__RefHeading___Toc24362)

[Table 6a. GO of GSEA analysis. 49](#__RefHeading___Toc24199)

[Table 6b. KEGG of GSEA analysis. 92](#__RefHeading___Toc24661)

[Appendix 7 96](#__RefHeading___Toc16807)

[Table 7. Immune Correlation Analysis. 96](#__RefHeading___Toc30254)

[Appendix 8 97](#__RefHeading___Toc26896)

[Table 8a. Gene-miRNA. 97](#__RefHeading___Toc20792)

[Table 8b. Gene-miRNA. 98](#__RefHeading___Toc16332)

# Appendix 1

**The clinical characteristics of patients**

**Table S1.** **The clinical characteristics of patients.**

| GSE58331 | | GSE105149 | |
| --- | --- | --- | --- |
| Variables | Number of samples | Variables | Number of samples |
| Gender |  | Gender |  |
| Male/Female | 19/56 | Male/Female | 9/18 |
| Diagnosis |  | Diagnosis |  |
| NSOI/Normal | 75/29 | NSOI/Normal | 27/7 |
| Tissue |  | Tissue |  |
| Anterior Orbit/  Lacrimal gland | 33/42 | Anterior Orbit/  Lacrimal gland | 0/27 |

# Appendix 2

## DEGs linked to NSOI

**Table S2. 314 DEGs linked to NSOI.**

| id | logFC | AveExpr | t | P.Value | adj.P.Val |
| --- | --- | --- | --- | --- | --- |
| HLF | -1.581770516 | 4.486953656 | -12.4363579 | 6.18E-26 | 1.34E-21 |
| MGST1 | -1.73101078 | 4.846522249 | -10.96290951 | 1.11E-21 | 7.99E-18 |
| C2orf40 | -2.196342714 | 4.519571821 | -10.79956686 | 3.24E-21 | 1.75E-17 |
| ADH1B | -2.095662677 | 4.158273041 | -10.74506249 | 4.64E-21 | 2.01E-17 |
| PGM1 | -1.196129698 | 5.644288109 | -10.66251442 | 7.97E-21 | 2.88E-17 |
| PGRMC2 | -1.258252644 | 4.200521305 | -10.61694307 | 1.07E-20 | 2.91E-17 |
| GSTM3 | -1.207615154 | 4.74005161 | -10.58382151 | 1.33E-20 | 3.21E-17 |
| NTRK2 | -1.228056221 | 4.525433831 | -10.52344673 | 1.98E-20 | 4.29E-17 |
| TGFBR3 | -1.886004737 | 5.252480398 | -10.44841687 | 3.23E-20 | 6.36E-17 |
| PPP1R1A | -2.187117989 | 5.727381115 | -10.11887501 | 2.75E-19 | 4.50E-16 |
| RNASE4 | -1.718212377 | 5.221705294 | -10.1073842 | 2.97E-19 | 4.50E-16 |
| APCDD1 | -1.512789702 | 5.715749609 | -10.09983731 | 3.11E-19 | 4.50E-16 |
| CAB39L | -1.030217143 | 4.949170449 | -10.08187051 | 3.50E-19 | 4.73E-16 |
| SLC24A3 | -1.125513094 | 4.313704729 | -10.01079712 | 5.54E-19 | 7.05E-16 |
| WLS | -1.149021225 | 5.203113407 | -9.907575363 | 1.08E-18 | 1.23E-15 |
| TUSC1 | -1.287879465 | 5.651620298 | -9.767610369 | 2.65E-18 | 2.87E-15 |
| STXBP1 | -1.412261334 | 4.671545908 | -9.680158969 | 4.64E-18 | 4.37E-15 |
| FAXDC2 | -1.056469312 | 4.96290536 | -9.62374166 | 6.66E-18 | 5.54E-15 |
| C1orf198 | -1.118940653 | 6.317302602 | -9.609476425 | 7.29E-18 | 5.54E-15 |
| NGFRAP1 | -1.325144028 | 6.64221447 | -9.607476998 | 7.38E-18 | 5.54E-15 |
| SEMA3G | -1.553875288 | 5.80514183 | -9.606545263 | 7.43E-18 | 5.54E-15 |
| TCIRG1 | 1.013294599 | 7.003690562 | 9.601458778 | 7.67E-18 | 5.54E-15 |
| PLIN1 | -3.510729005 | 6.206463925 | -9.564801157 | 9.70E-18 | 6.77E-15 |
| TIMP4 | -1.776944016 | 3.777321203 | -9.540191143 | 1.13E-17 | 7.44E-15 |
| GPAM | -1.964251439 | 3.992002302 | -9.52302724 | 1.27E-17 | 7.80E-15 |
| CDR1 | -2.144905233 | 6.179538535 | -9.519141461 | 1.30E-17 | 7.80E-15 |
| NECAB1 | -1.034647256 | 2.169721244 | -9.502402166 | 1.44E-17 | 8.09E-15 |
| LEPR | -2.206002435 | 3.940087912 | -9.496928317 | 1.49E-17 | 8.09E-15 |
| CAV2 | -1.337869546 | 4.75307031 | -9.47127428 | 1.76E-17 | 9.28E-15 |
| GHR | -2.038120615 | 3.529538566 | -9.433388079 | 2.24E-17 | 1.03E-14 |
| MEST | -2.032412974 | 3.995469591 | -9.413087554 | 2.55E-17 | 1.15E-14 |
| NDNF | -1.929794836 | 4.293116528 | -9.351750842 | 3.76E-17 | 1.66E-14 |
| HADH | -1.089676098 | 4.925785437 | -9.320925836 | 4.56E-17 | 1.94E-14 |
| IGFBP6 | -1.895419766 | 6.719933425 | -9.308710627 | 4.93E-17 | 2.05E-14 |
| OLFML2A | -1.419877286 | 5.287243312 | -9.288056282 | 5.62E-17 | 2.30E-14 |
| PRDX6 | -1.024201576 | 6.82029148 | -9.257464531 | 6.82E-17 | 2.59E-14 |
| ACACB | -1.225419329 | 6.038983591 | -9.242358839 | 7.50E-17 | 2.80E-14 |
| MTURN | -1.222611145 | 5.378674298 | -9.233041454 | 7.95E-17 | 2.92E-14 |
| CPE | -1.257137939 | 4.715130972 | -9.215894478 | 8.86E-17 | 3.14E-14 |
| TRHDE-AS1 | -1.518368089 | 3.078249473 | -9.190156933 | 1.04E-16 | 3.58E-14 |
| IGSF10 | -1.151645472 | 4.12122447 | -9.179030936 | 1.12E-16 | 3.78E-14 |
| LIMD2 | 1.096609514 | 7.559429116 | 9.162129238 | 1.24E-16 | 4.14E-14 |
| NPR3 | -1.532985416 | 3.501358447 | -9.113191883 | 1.69E-16 | 5.46E-14 |
| LOC284825 | -1.372371338 | 3.039392288 | -9.088822552 | 1.97E-16 | 6.27E-14 |
| PPAP2B | -1.546183631 | 6.72891155 | -9.078431504 | 2.10E-16 | 6.60E-14 |
| ENPP6 | -1.20312907 | 2.732761455 | -8.99736236 | 3.49E-16 | 1.03E-13 |
| NFIA | -1.027794466 | 4.963336742 | -8.980603043 | 3.88E-16 | 1.12E-13 |
| PBX1 | -1.44655793 | 6.592959362 | -8.95283287 | 4.61E-16 | 1.23E-13 |
| EFHD1 | -1.347095261 | 6.250872915 | -8.947018061 | 4.78E-16 | 1.24E-13 |
| TMEM100 | -1.245035632 | 2.621077458 | -8.93205661 | 5.25E-16 | 1.31E-13 |
| SORBS1 | -1.16278045 | 5.510134543 | -8.931606383 | 5.27E-16 | 1.31E-13 |
| MAOA | -1.958420887 | 5.489570158 | -8.921310044 | 5.61E-16 | 1.38E-13 |
| ADIPOQ | -3.045245553 | 3.959666935 | -8.881446745 | 7.20E-16 | 1.73E-13 |
| CORO2B | -1.167529165 | 4.368560636 | -8.879327674 | 7.29E-16 | 1.73E-13 |
| FTO | -1.022722841 | 7.177143322 | -8.877744447 | 7.36E-16 | 1.73E-13 |
| RUNX1-IT1 | 1.129771198 | 5.125247074 | 8.864406406 | 8.00E-16 | 1.84E-13 |
| ADIRF | -1.745871197 | 8.12664587 | -8.844058477 | 9.08E-16 | 2.05E-13 |
| ITGB2-AS1 | 1.227307212 | 5.904472474 | 8.841717787 | 9.21E-16 | 2.06E-13 |
| FHL1 | -2.092149128 | 6.045253917 | -8.816132924 | 1.08E-15 | 2.36E-13 |
| RNF11 | -1.535841555 | 3.935163673 | -8.800732561 | 1.19E-15 | 2.57E-13 |
| CHRDL1 | -1.698377913 | 5.787419576 | -8.684949938 | 2.43E-15 | 5.11E-13 |
| FABP4 | -2.353748214 | 5.206550928 | -8.682245195 | 2.47E-15 | 5.15E-13 |
| PLSCR4 | -1.274757896 | 2.985046822 | -8.655960692 | 2.91E-15 | 5.78E-13 |
| TNMD | -1.04518328 | 2.791647023 | -8.650148707 | 3.01E-15 | 5.93E-13 |
| SH3D19 | -1.22465157 | 5.719367165 | -8.627295814 | 3.47E-15 | 6.71E-13 |
| PHYH | -1.291005106 | 4.511931621 | -8.615465443 | 3.73E-15 | 7.09E-13 |
| PCDH18 | -1.299308935 | 5.42226592 | -8.559750062 | 5.26E-15 | 9.73E-13 |
| TBC1D10C | 1.473425607 | 6.577682583 | 8.558349206 | 5.30E-15 | 9.73E-13 |
| EPHX1 | -1.206806765 | 7.467876504 | -8.552855759 | 5.48E-15 | 9.89E-13 |
| PPAP2A | -1.136882346 | 5.91326828 | -8.544351478 | 5.78E-15 | 1.03E-12 |
| PCOLCE2 | -1.515874871 | 3.030533398 | -8.542001332 | 5.86E-15 | 1.03E-12 |
| CIDEC | -2.41117819 | 5.944620287 | -8.535646536 | 6.09E-15 | 1.06E-12 |
| KLB | -1.119367337 | 2.353027435 | -8.532625108 | 6.21E-15 | 1.08E-12 |
| LYVE1 | -2.244951922 | 5.215787478 | -8.524917355 | 6.51E-15 | 1.10E-12 |
| ASPA | -1.055274718 | 3.669429502 | -8.523573255 | 6.56E-15 | 1.10E-12 |
| MIAT | 1.019026479 | 5.24153807 | 8.519312101 | 6.73E-15 | 1.12E-12 |
| HCAR1 | -1.119209236 | 3.777418405 | -8.469317001 | 9.14E-15 | 1.45E-12 |
| PARVG | 1.094550292 | 5.516760352 | 8.460115639 | 9.67E-15 | 1.51E-12 |
| MAMDC2 | -1.730469482 | 3.118799264 | -8.445980461 | 1.05E-14 | 1.63E-12 |
| HSPB6 | -2.539368644 | 7.497621453 | -8.431807843 | 1.15E-14 | 1.75E-12 |
| CETN2 | -1.087280636 | 5.78650944 | -8.423713089 | 1.21E-14 | 1.82E-12 |
| PPL | -1.710548579 | 6.388411778 | -8.395003875 | 1.44E-14 | 2.09E-12 |
| BNIP3L | -1.051369981 | 6.860025447 | -8.387225216 | 1.51E-14 | 2.18E-12 |
| VIT | -1.675552155 | 4.902205109 | -8.368419053 | 1.69E-14 | 2.43E-12 |
| THRSP | -1.636316122 | 4.64274161 | -8.34165597 | 1.99E-14 | 2.71E-12 |
| PCYOX1 | -1.299089014 | 5.422457266 | -8.341566259 | 1.99E-14 | 2.71E-12 |
| HLA-F | 1.078545985 | 7.814018991 | 8.321710672 | 2.25E-14 | 2.97E-12 |
| YAP1 | -1.111039667 | 5.665259802 | -8.270311849 | 3.07E-14 | 3.93E-12 |
| ACSL1 | -1.319124186 | 6.906560476 | -8.26815685 | 3.11E-14 | 3.96E-12 |
| CNTFR | -1.158708488 | 6.058542253 | -8.199593737 | 4.70E-14 | 5.66E-12 |
| CAP2 | -1.065065275 | 3.657663217 | -8.197426883 | 4.77E-14 | 5.70E-12 |
| ATXN1L | -1.042632367 | 5.41128383 | -8.187837241 | 5.05E-14 | 5.94E-12 |
| IGHM | 1.893953592 | 8.464685567 | 8.16003212 | 5.97E-14 | 6.91E-12 |
| MN1 | -1.570698055 | 5.292246061 | -8.154754024 | 6.16E-14 | 7.10E-12 |
| GSTM5 | -1.25620581 | 4.944432756 | -8.152929983 | 6.23E-14 | 7.14E-12 |
| ACADL | -1.104516836 | 3.410072407 | -8.151645618 | 6.28E-14 | 7.15E-12 |
| TMEM132C | -1.267317386 | 4.067071738 | -8.100494497 | 8.54E-14 | 9.29E-12 |
| SCARA5 | -1.193473518 | 5.890702792 | -8.064299792 | 1.06E-13 | 1.11E-11 |
| PTPRCAP | 1.133661289 | 7.237678373 | 8.048820964 | 1.16E-13 | 1.21E-11 |
| NAPSB | 1.030717746 | 4.947260779 | 8.043680529 | 1.20E-13 | 1.24E-11 |
| OMD | -1.322607942 | 3.923039324 | -8.029980482 | 1.30E-13 | 1.34E-11 |
| MFAP4 | -1.601286777 | 6.91438899 | -8.02214008 | 1.36E-13 | 1.39E-11 |
| ADAM28 | 1.102305993 | 4.784065539 | 8.018397614 | 1.40E-13 | 1.40E-11 |
| RBP4 | -1.174535717 | 4.410902601 | -8.006856523 | 1.50E-13 | 1.48E-11 |
| CALB2 | -1.253426532 | 3.963233847 | -8.003234146 | 1.53E-13 | 1.50E-11 |
| GYG2 | -1.254679436 | 4.610246054 | -7.997454507 | 1.58E-13 | 1.54E-11 |
| PDK4 | -1.426209986 | 4.540221122 | -7.994998707 | 1.60E-13 | 1.55E-11 |
| LRRN4CL | -1.726727881 | 4.424048701 | -7.980499892 | 1.75E-13 | 1.65E-11 |
| AKR1C1 | -1.041371399 | 5.59018636 | -7.972903639 | 1.83E-13 | 1.71E-11 |
| EHBP1 | -1.14554198 | 5.982775699 | -7.953103321 | 2.06E-13 | 1.89E-11 |
| APOD | -1.882244524 | 9.838764755 | -7.927534736 | 2.40E-13 | 2.16E-11 |
| TSKU | -1.150911186 | 5.787482441 | -7.917882808 | 2.54E-13 | 2.27E-11 |
| TRBC1 | 1.815085432 | 6.583157486 | 7.91284724 | 2.62E-13 | 2.32E-11 |
| CAV1 | -1.813912368 | 6.854425452 | -7.90816649 | 2.69E-13 | 2.37E-11 |
| NDFIP1 | -1.112314401 | 6.026818655 | -7.895332306 | 2.90E-13 | 2.52E-11 |
| CRYAB | -1.961025916 | 8.096120564 | -7.893923502 | 2.93E-13 | 2.52E-11 |
| RP11-736K20.5 | -1.151628229 | 5.633634195 | -7.86614755 | 3.45E-13 | 2.92E-11 |
| IGLL3P | 1.348670984 | 9.057507242 | 7.861481713 | 3.55E-13 | 2.99E-11 |
| BC022047 | -1.207117032 | 4.802307286 | -7.846896828 | 3.87E-13 | 3.25E-11 |
| AMOTL2 | -1.25204271 | 5.288509815 | -7.832080887 | 4.22E-13 | 3.48E-11 |
| DENND1C | 1.280791781 | 6.090037587 | 7.829675803 | 4.28E-13 | 3.51E-11 |
| CORO1A | 1.490258951 | 7.766740844 | 7.823698118 | 4.44E-13 | 3.61E-11 |
| ITGAL | 1.261781787 | 5.975292808 | 7.81434834 | 4.69E-13 | 3.79E-11 |
| IL21R | 1.223039408 | 5.004894929 | 7.799184731 | 5.13E-13 | 4.10E-11 |
| LINC00657 | -1.10323622 | 5.357381967 | -7.794978056 | 5.26E-13 | 4.17E-11 |
| SGCG | -1.598499332 | 3.15631333 | -7.782610437 | 5.65E-13 | 4.45E-11 |
| CCL19 | 1.818238625 | 7.880550521 | 7.778598006 | 5.79E-13 | 4.51E-11 |
| TCF7L1 | -1.136132121 | 6.152244124 | -7.771954361 | 6.02E-13 | 4.67E-11 |
| IRF8 | 1.233530672 | 6.102711564 | 7.771393236 | 6.04E-13 | 4.67E-11 |
| FZD4 | -1.034166834 | 5.741148556 | -7.758446742 | 6.52E-13 | 4.99E-11 |
| DNMBP | -1.051034251 | 5.196394381 | -7.75455089 | 6.67E-13 | 5.09E-11 |
| MRAP | -1.018747691 | 3.573749337 | -7.742089943 | 7.18E-13 | 5.36E-11 |
| TSPAN6 | -1.021555172 | 4.345915851 | -7.73172439 | 7.63E-13 | 5.64E-11 |
| PDGFD | -1.357957301 | 4.912871545 | -7.720007191 | 8.17E-13 | 6.02E-11 |
| PAM | -1.112423739 | 5.885799585 | -7.709756918 | 8.68E-13 | 6.34E-11 |
| FMO2 | -1.379755451 | 5.885950942 | -7.703198359 | 9.02E-13 | 6.55E-11 |
| OLFML1 | -1.313114257 | 3.40833212 | -7.70078897 | 9.15E-13 | 6.62E-11 |
| HLA-B | 1.163607639 | 10.93628287 | 7.681355193 | 1.02E-12 | 7.28E-11 |
| MMP9 | 1.965916767 | 6.857354614 | 7.666651644 | 1.12E-12 | 7.70E-11 |
| IL7R | 1.732257154 | 5.609671444 | 7.664092123 | 1.13E-12 | 7.80E-11 |
| TMEM200B | -1.061462554 | 4.505638146 | -7.661038192 | 1.15E-12 | 7.86E-11 |
| AOC3 | -1.977719355 | 4.353420703 | -7.651111705 | 1.22E-12 | 8.25E-11 |
| CD74 | 1.119709874 | 8.834651006 | 7.632957925 | 1.36E-12 | 9.04E-11 |
| CRNDE | -1.293840002 | 4.568632288 | -7.631385291 | 1.37E-12 | 9.09E-11 |
| IL32 | 1.283633762 | 7.748202392 | 7.630424906 | 1.38E-12 | 9.11E-11 |
| ZNF423 | -1.299917264 | 4.837136643 | -7.620270671 | 1.46E-12 | 9.64E-11 |
| TUBB2A | -1.522843019 | 4.519781287 | -7.590925442 | 1.74E-12 | 1.12E-10 |
| LAPTM4A | -1.020146223 | 7.062747291 | -7.585002271 | 1.80E-12 | 1.15E-10 |
| CIITA | 1.434128152 | 5.82955887 | 7.584999067 | 1.80E-12 | 1.15E-10 |
| LCK | 1.207272363 | 5.133001308 | 7.556984982 | 2.12E-12 | 1.33E-10 |
| CA3 | -1.397029769 | 2.871505982 | -7.540561679 | 2.33E-12 | 1.45E-10 |
| CHI3L1 | 1.911130703 | 5.577084066 | 7.540506624 | 2.33E-12 | 1.45E-10 |
| CYBRD1 | -1.32368824 | 6.412982308 | -7.525924649 | 2.54E-12 | 1.57E-10 |
| AQPEP | -1.082296344 | 2.474334542 | -7.522886587 | 2.58E-12 | 1.59E-10 |
| MYOC | -1.426256726 | 4.267750326 | -7.5181086 | 2.65E-12 | 1.62E-10 |
| ALDH1A1 | -1.157776409 | 5.690677596 | -7.500332159 | 2.94E-12 | 1.78E-10 |
| PRELP | -1.368875484 | 6.347271132 | -7.486383941 | 3.19E-12 | 1.89E-10 |
| TRAC | 1.214385275 | 7.322602675 | 7.485462438 | 3.21E-12 | 1.90E-10 |
| MARC1 | -1.092510242 | 4.381581801 | -7.476964151 | 3.37E-12 | 1.97E-10 |
| BNIP3 | -1.235396841 | 5.675988924 | -7.461065531 | 3.69E-12 | 2.15E-10 |
| ITGB2 | 1.165071216 | 6.893631197 | 7.456119994 | 3.80E-12 | 2.20E-10 |
| EBF1 | -1.318238618 | 4.626904283 | -7.449743627 | 3.94E-12 | 2.28E-10 |
| SIGLEC10 | 1.224329637 | 5.535788687 | 7.432731334 | 4.35E-12 | 2.48E-10 |
| CD36 | -1.607165225 | 4.534679053 | -7.422677356 | 4.61E-12 | 2.62E-10 |
| DDR2 | -1.102453345 | 6.035613186 | -7.407888293 | 5.02E-12 | 2.81E-10 |
| ZNF667-AS1 | -1.008788072 | 5.637265115 | -7.393656611 | 5.44E-12 | 3.02E-10 |
| CD34 | -1.256004889 | 6.513814211 | -7.388224299 | 5.62E-12 | 3.10E-10 |
| RHOH | 1.247725263 | 4.641017917 | 7.386803594 | 5.66E-12 | 3.10E-10 |
| USP54 | -1.17571173 | 6.102203696 | -7.38439834 | 5.74E-12 | 3.14E-10 |
| GBE1 | -1.027826069 | 3.210398081 | -7.380737672 | 5.86E-12 | 3.20E-10 |
| IL2RG | 1.749958396 | 7.818285751 | 7.3621074 | 6.53E-12 | 3.51E-10 |
| CFH | -1.350738672 | 5.556135523 | -7.326546868 | 8.00E-12 | 4.18E-10 |
| SLC19A3 | -1.019896884 | 2.904428349 | -7.315742541 | 8.51E-12 | 4.40E-10 |
| TIMP3 | -1.151600079 | 8.030378699 | -7.307362679 | 8.93E-12 | 4.57E-10 |
| MAP1B | -1.006337056 | 5.53842632 | -7.304561661 | 9.07E-12 | 4.63E-10 |
| AOX1 | -1.32271604 | 5.252556404 | -7.296763057 | 9.49E-12 | 4.80E-10 |
| SMIM19 | -1.135827832 | 6.72484084 | -7.291701762 | 9.77E-12 | 4.92E-10 |
| SRPX | -1.158450028 | 6.219664492 | -7.261717257 | 1.16E-11 | 5.66E-10 |
| CD3D | 1.245146874 | 6.29149497 | 7.255452129 | 1.20E-11 | 5.86E-10 |
| PALMD | -1.118219182 | 5.186982933 | -7.252568101 | 1.22E-11 | 5.91E-10 |
| PLA2G2D | 1.941342796 | 5.961330087 | 7.216571198 | 1.50E-11 | 7.11E-10 |
| AKR1C3 | -1.426982657 | 4.677849082 | -7.21184206 | 1.54E-11 | 7.28E-10 |
| SLC2A10 | -1.062148436 | 3.718929416 | -7.188258164 | 1.76E-11 | 8.11E-10 |
| PCK1 | -1.475504303 | 2.760272338 | -7.171284609 | 1.94E-11 | 8.83E-10 |
| TMEM47 | -1.095040723 | 5.043745766 | -7.170583658 | 1.94E-11 | 8.85E-10 |
| SPOCK2 | 1.057674206 | 6.376214336 | 7.163844744 | 2.02E-11 | 9.13E-10 |
| SYNM | -1.223555572 | 5.660397548 | -7.161471189 | 2.05E-11 | 9.22E-10 |
| SLAMF8 | 1.501661586 | 5.432453358 | 7.136917355 | 2.35E-11 | 1.04E-09 |
| SH2D2A | 1.02217402 | 4.290105568 | 7.126504762 | 2.49E-11 | 1.09E-09 |
| CXCR4 | 1.975273786 | 7.0885297 | 7.117572984 | 2.62E-11 | 1.14E-09 |
| OSR2 | -1.16102895 | 6.19367835 | -7.113665127 | 2.68E-11 | 1.16E-09 |
| NTN4 | -1.127100992 | 4.982780396 | -7.080033241 | 3.24E-11 | 1.37E-09 |
| APOC1 | 1.21309651 | 6.387892148 | 7.076888294 | 3.30E-11 | 1.39E-09 |
| PLAT | -1.421291584 | 4.790006992 | -7.026460628 | 4.38E-11 | 1.79E-09 |
| OLFM2 | -1.141597923 | 4.3231814 | -7.020144185 | 4.54E-11 | 1.85E-09 |
| BOK | -1.194484161 | 5.216390864 | -7.016088349 | 4.64E-11 | 1.88E-09 |
| ADAMTS5 | -1.013975928 | 3.0334763 | -7.014385822 | 4.69E-11 | 1.90E-09 |
| SLAMF7 | 1.596148424 | 6.514155877 | 6.975891792 | 5.81E-11 | 2.29E-09 |
| ZFPM2 | -1.016389112 | 2.986151041 | -6.973933453 | 5.87E-11 | 2.30E-09 |
| LPL | -1.925281129 | 5.328549029 | -6.951966106 | 6.64E-11 | 2.59E-09 |
| CD52 | 1.595727299 | 7.748757371 | 6.951044468 | 6.67E-11 | 2.59E-09 |
| CD3G | 1.478065164 | 5.395383945 | 6.944285197 | 6.93E-11 | 2.67E-09 |
| OGN | -1.595707784 | 4.206741741 | -6.937697978 | 7.19E-11 | 2.75E-09 |
| RAC2 | 1.129469682 | 7.247281587 | 6.935011038 | 7.30E-11 | 2.78E-09 |
| PIM2 | 1.004702341 | 6.3433007 | 6.925928745 | 7.67E-11 | 2.91E-09 |
| IGKC | 1.149788899 | 6.840816581 | 6.876534517 | 1.01E-10 | 3.74E-09 |
| TCEAL8 | -1.081951075 | 4.592707896 | -6.864460496 | 1.08E-10 | 3.97E-09 |
| CAPG | 1.179274116 | 7.163397946 | 6.846296465 | 1.19E-10 | 4.33E-09 |
| ARHGAP9 | 1.030845182 | 6.105324891 | 6.845978484 | 1.20E-10 | 4.33E-09 |
| EPDR1 | -1.24642265 | 3.867164416 | -6.838662065 | 1.24E-10 | 4.49E-09 |
| ZBTB16 | -1.305367643 | 5.409064229 | -6.83702477 | 1.26E-10 | 4.53E-09 |
| NLRC5 | 1.016800132 | 5.898174776 | 6.807648334 | 1.48E-10 | 5.22E-09 |
| OSR1 | -1.000504653 | 5.636313275 | -6.804187172 | 1.51E-10 | 5.31E-09 |
| RHOBTB3 | -1.117969406 | 5.210508454 | -6.787559726 | 1.65E-10 | 5.77E-09 |
| IGLJ3 | 1.02644592 | 6.81973377 | 6.778231179 | 1.74E-10 | 6.04E-09 |
| P2RX5 | 1.060760276 | 5.12710244 | 6.75317515 | 1.99E-10 | 6.82E-09 |
| CD3E | 1.561531912 | 5.541127794 | 6.742006543 | 2.12E-10 | 7.19E-09 |
| CCR7 | 1.338763659 | 5.055349796 | 6.741152332 | 2.13E-10 | 7.21E-09 |
| SCP2 | -1.077088785 | 5.793161385 | -6.740570441 | 2.13E-10 | 7.22E-09 |
| LYRM5 | -1.13419933 | 4.539770399 | -6.733795972 | 2.22E-10 | 7.45E-09 |
| BCL11B | 1.0951051 | 4.572459544 | 6.733694669 | 2.22E-10 | 7.45E-09 |
| HIST1H3B | 1.650285849 | 6.23072074 | 6.705357548 | 2.59E-10 | 8.48E-09 |
| HLA-DRA | 1.490874383 | 10.26462261 | 6.651994987 | 3.46E-10 | 1.10E-08 |
| IGLC1 | 1.153810093 | 8.250421542 | 6.61978314 | 4.12E-10 | 1.28E-08 |
| GPX3 | -1.05125436 | 8.821861482 | -6.599824899 | 4.59E-10 | 1.41E-08 |
| AIM2 | 1.505274751 | 5.359298996 | 6.575542935 | 5.24E-10 | 1.59E-08 |
| LRRC15 | 1.239644866 | 4.45867228 | 6.567156037 | 5.48E-10 | 1.66E-08 |
| CFD | -1.481985765 | 7.323610326 | -6.543136337 | 6.24E-10 | 1.86E-08 |
| ANG | -1.238370577 | 6.004427997 | -6.519212903 | 7.10E-10 | 2.07E-08 |
| LGALS2 | 1.002819521 | 6.071646918 | 6.477349645 | 8.89E-10 | 2.53E-08 |
| IKZF3 | 1.260458112 | 5.386650118 | 6.469288868 | 9.28E-10 | 2.61E-08 |
| HLA-DMB | 1.096883331 | 7.822380059 | 6.44710624 | 1.04E-09 | 2.89E-08 |
| PLIN4 | -1.476723008 | 5.929041061 | -6.438505304 | 1.09E-09 | 3.01E-08 |
| IGFBP5 | -1.012988145 | 8.32584525 | -6.413168754 | 1.25E-09 | 3.36E-08 |
| PFN2 | -1.305808632 | 7.191459458 | -6.408058139 | 1.29E-09 | 3.42E-08 |
| LY86 | 1.046716811 | 5.00331236 | 6.40602675 | 1.30E-09 | 3.44E-08 |
| ADAMDEC1 | 1.843955589 | 3.328738653 | 6.378087762 | 1.51E-09 | 3.93E-08 |
| SGCE | -1.002458275 | 6.624569504 | -6.373257622 | 1.55E-09 | 4.02E-08 |
| C6 | -1.075281143 | 4.109097165 | -6.351233594 | 1.74E-09 | 4.46E-08 |
| RAMP2 | -1.013776794 | 6.509320046 | -6.316218865 | 2.09E-09 | 5.17E-08 |
| FAIM3 | 1.259183341 | 6.207656375 | 6.310017643 | 2.16E-09 | 5.31E-08 |
| TAP1 | 1.035927974 | 7.103372959 | 6.302633084 | 2.25E-09 | 5.49E-08 |
| HLA-DPB1 | 1.088401152 | 6.262672368 | 6.265027357 | 2.74E-09 | 6.54E-08 |
| LAPTM5 | 1.395936968 | 8.426366691 | 6.255967641 | 2.87E-09 | 6.83E-08 |
| HIST1H3F | 1.46423071 | 4.70721114 | 6.224954467 | 3.38E-09 | 7.86E-08 |
| MXI1 | -1.011232551 | 5.819021654 | -6.2094364 | 3.67E-09 | 8.39E-08 |
| CD53 | 1.005734718 | 5.352609466 | 6.207567156 | 3.70E-09 | 8.45E-08 |
| PRKAR2B | -1.277940389 | 3.100586588 | -6.193114434 | 3.99E-09 | 8.97E-08 |
| YWHAG | -1.001278301 | 6.190991046 | -6.178280862 | 4.32E-09 | 9.61E-08 |
| GPR174 | 1.317450106 | 4.278934985 | 6.171956195 | 4.46E-09 | 9.90E-08 |
| CD19 | 1.115698073 | 5.32371994 | 6.158886774 | 4.77E-09 | 1.05E-07 |
| CXCL13 | 2.100461667 | 4.55167995 | 6.139910294 | 5.27E-09 | 1.13E-07 |
| G0S2 | -1.709121439 | 6.59574315 | -6.083459492 | 7.06E-09 | 1.46E-07 |
| KLHL6 | 1.06375426 | 4.479001529 | 6.077159222 | 7.29E-09 | 1.50E-07 |
| AC079767.4 | 1.032956019 | 3.602282621 | 6.071573416 | 7.50E-09 | 1.55E-07 |
| CXCL9 | 2.182766208 | 6.053932717 | 6.049156624 | 8.42E-09 | 1.70E-07 |
| SLAMF6 | 1.00606887 | 5.37489056 | 6.045041689 | 8.60E-09 | 1.73E-07 |
| ITM2A | -1.087307223 | 6.006359724 | -6.038047074 | 8.92E-09 | 1.78E-07 |
| SERINC1 | -1.030373963 | 5.089049494 | -5.961587453 | 1.32E-08 | 2.52E-07 |
| NLRC3 | 1.192798109 | 4.711440758 | 5.93934475 | 1.48E-08 | 2.78E-07 |
| CCNG1 | -1.037486926 | 5.992720497 | -5.926777815 | 1.57E-08 | 2.93E-07 |
| GZMK | 1.117171872 | 6.167266707 | 5.90482329 | 1.76E-08 | 3.24E-07 |
| UAP1 | -1.07203366 | 5.597160481 | -5.863476701 | 2.17E-08 | 3.90E-07 |
| CD2 | 1.197652592 | 4.33768524 | 5.862259812 | 2.18E-08 | 3.92E-07 |
| CD22 | 1.085579562 | 5.465080104 | 5.852446389 | 2.29E-08 | 4.10E-07 |
| FCGR1B | 1.510880221 | 4.936964755 | 5.828906725 | 2.58E-08 | 4.54E-07 |
| TOB1 | -1.061302278 | 3.755601796 | -5.825942223 | 2.62E-08 | 4.59E-07 |
| SASH3 | 1.075270983 | 5.819402899 | 5.782346731 | 3.26E-08 | 5.59E-07 |
| IGLV1-44 | 1.161620861 | 7.60313081 | 5.761726551 | 3.61E-08 | 6.13E-07 |
| CCL4 | 1.094390248 | 5.643877075 | 5.747569268 | 3.88E-08 | 6.54E-07 |
| GBP5 | 1.131562932 | 5.107788168 | 5.735730064 | 4.11E-08 | 6.89E-07 |
| BCL2A1 | 1.307378359 | 3.996057213 | 5.632881725 | 6.84E-08 | 1.09E-06 |
| PTPRC | 1.107499382 | 4.84825036 | 5.631931166 | 6.87E-08 | 1.10E-06 |
| SMIM3 | -1.002676384 | 4.890902125 | -5.631884628 | 6.88E-08 | 1.10E-06 |
| CXCL10 | 1.737636753 | 5.835419282 | 5.511953194 | 1.24E-07 | 1.83E-06 |
| KCNA3 | 1.114739091 | 5.112487954 | 5.499728776 | 1.31E-07 | 1.92E-06 |
| PLAC8 | 1.182853097 | 5.277176525 | 5.447246744 | 1.69E-07 | 2.39E-06 |
| CPA3 | -1.245141593 | 4.39787271 | -5.43013705 | 1.83E-07 | 2.57E-06 |
| SPP1 | 1.37301198 | 3.822137766 | 5.429056322 | 1.84E-07 | 2.58E-06 |
| MS4A1 | 1.663717969 | 4.667887221 | 5.415956785 | 1.96E-07 | 2.72E-06 |
| CLMP | -1.029124833 | 5.444183565 | -5.400631519 | 2.11E-07 | 2.89E-06 |
| IGLL5 | 1.322481113 | 8.465721166 | 5.394985517 | 2.17E-07 | 2.95E-06 |
| CHIT1 | 1.30940756 | 5.146673618 | 5.378751611 | 2.34E-07 | 3.15E-06 |
| HIST1H3C | 1.019628143 | 5.73248254 | 5.323446474 | 3.05E-07 | 3.94E-06 |
| HBB | -1.080979986 | 6.406081225 | -5.321388823 | 3.08E-07 | 3.98E-06 |
| SELL | 1.437493857 | 5.710514646 | 5.273483799 | 3.86E-07 | 4.80E-06 |
| RASGRP1 | 1.017947496 | 3.969639249 | 5.256653112 | 4.18E-07 | 5.16E-06 |
| MIR100HG | -1.242104796 | 4.886247778 | -5.175602739 | 6.11E-07 | 7.18E-06 |
| SFRP1 | -1.230196944 | 7.018231023 | -5.127128204 | 7.65E-07 | 8.71E-06 |
| ANKRD22 | 1.051363787 | 3.983796073 | 5.119110825 | 7.94E-07 | 8.97E-06 |
| C15orf48 | 1.281464983 | 3.22296987 | 5.075988112 | 9.68E-07 | 1.06E-05 |
| TCL1A | 1.257197154 | 5.399322988 | 5.010341921 | 1.31E-06 | 1.38E-05 |
| FGR | 1.08695019 | 5.498696616 | 4.969528169 | 1.57E-06 | 1.63E-05 |
| PLA2G7 | 1.12575966 | 5.037218284 | 4.957730743 | 1.66E-06 | 1.70E-05 |
| LCP1 | 1.025456824 | 6.403611786 | 4.923869065 | 1.93E-06 | 1.96E-05 |
| MYO5C | -1.043270702 | 5.043093603 | -4.91515382 | 2.01E-06 | 2.02E-05 |
| TCEAL2 | -1.189281706 | 5.049764006 | -4.851217179 | 2.67E-06 | 2.60E-05 |
| HLA-DPA1 | 1.008384359 | 7.742546736 | 4.737146008 | 4.42E-06 | 4.03E-05 |
| IDO1 | 1.219714862 | 4.418865866 | 4.621533421 | 7.31E-06 | 6.19E-05 |
| VCAN | 1.090763313 | 5.834654181 | 4.613687722 | 7.56E-06 | 6.36E-05 |
| IRX3 | -1.027393767 | 5.052762569 | -4.447355332 | 1.53E-05 | 0.00011679 |
| MMP7 | 1.092485954 | 5.343884158 | 4.420635906 | 1.71E-05 | 0.000128681 |
| MMP10 | 1.053870613 | 3.573200734 | 4.298668576 | 2.83E-05 | 0.000199138 |
| CYTIP | 1.060044386 | 5.519318204 | 4.275401124 | 3.11E-05 | 0.000215791 |
| FDCSP | 1.956398989 | 6.414984501 | 4.125723389 | 5.67E-05 | 0.000364805 |
| IGHD | 1.056196393 | 7.183395927 | 4.016060013 | 8.73E-05 | 0.000530533 |
| FNDC1 | 1.0681358 | 4.100637785 | 3.965248027 | 0.000106221 | 0.000628104 |
| CCL18 | 1.247785171 | 6.113480614 | 3.851379283 | 0.000163884 | 0.000904832 |
| OLFM4 | 1.125572769 | 3.714594958 | 3.412097176 | 0.000798527 | 0.003445472 |
| LYZ | 1.424552745 | 10.4266248 | 3.394247398 | 0.000849001 | 0.003622517 |
| HLA-DQA1 | 1.526563848 | 7.029904979 | 3.196540199 | 0.001646922 | 0.006291893 |
| IGJ | 1.298367998 | 7.610171178 | 3.091887458 | 0.002310462 | 0.00830715 |
| LTF | 1.826715569 | 10.28926435 | 3.07840849 | 0.00241194 | 0.008612821 |
| SCGB2A2 | -1.013207333 | 3.893518563 | -2.608050163 | 0.009882211 | 0.027107854 |

# Appendix 3

**LASSO and** **SVM-RFE genes**

**Table S3a. LASSO genes**.

| HLF | IGSF10 | F8 | PALMD | C6 | GPR34 |
| --- | --- | --- | --- | --- | --- |
| PGM1 | PLSCR4 | IRF8 | SMIM19 | RHOBTB3 | FBP1 |
| GPR146 | ENPP6 | MAP1B | TNS1 | CCL4 | SMR3B |
| GSTM3 | NECAB1 | IGLL3P | CD3E | IGK | CPA3 |
| TCIRG1 | ATXN1L | PLA2G16 | LRRC15 | CD79A | HIST1H2BM |
| TIMP4 | AKR1C1 | SPOCK2 | GBE1 | TCEAL8 | IDO1 |
| CORO2B | MARC1 | APOC1 | MGLL | SUCLA2 | FGR |
| RUNX1-IT1 | COX7A1 | RBPMS2 | TMEM47 | ALDH2 | IGHD |
| LYVE1 | TMEM200B | UGDH | ADAMDEC1 | RCAN2 | FDCSP |
| PARVG | CALB2 | ZBTB16 | HLA-DPB1 | SPP1 | OLFM4 |
| CCL18 |  |  |  |  |  |

**Table S3b. SVM-RFE genes**.

| IGK | TUSC1 | OLFML1 | MIR100HG | MAP1B | IGJ |
| --- | --- | --- | --- | --- | --- |
| IRF8 | AKR1C1 | GPR146 | SASH3 | TNMD | DENND1C |
| HLF | RHOBTB3 | PALMD | CCL4 | CORO2B | ENPP6 |
| PGM1 | TNS1 | PLA2G16 | IGSF10 |  |  |

**Table S3c. InterGenes**.

| HLF | IGK | CCL4 | TNS1 | MAP1B |
| --- | --- | --- | --- | --- |
| PGM1 | CORO2B | PLA2G16 | RHOBTB3 | AKR1C1 |
| GPR146 | IGSF10 | PALMD | ENPP6 | IRF8 |

# Appendix 4

## DEG Identification of GPR146

**Table S4. 133 DEG Identification of** GPR146

| id | logFC | AveExpr | t | P.Value |
| --- | --- | --- | --- | --- |
| HIST1H3B | -1.84744916 | 6.636219548 | -8.814073386 | 5.45E-15 |
| HIST1H3F | -1.749747778 | 5.066993543 | -8.6030176 | 1.79E-14 |
| IL21R | -1.131332867 | 5.305413184 | -8.285674459 | 1.06E-13 |
| TAP1 | -1.162933503 | 7.357915262 | -8.239199112 | 1.37E-13 |
| IL32 | -1.178358679 | 8.063609545 | -8.208936854 | 1.62E-13 |
| LCK | -1.08293292 | 5.429645374 | -8.137184896 | 2.41E-13 |
| HIST1H3C | -1.368412048 | 5.983019741 | -7.956183478 | 6.53E-13 |
| MMP9 | -1.801811967 | 7.340408448 | -7.871575236 | 1.04E-12 |
| TRBC1 | -1.517793578 | 7.029149906 | -7.868940295 | 1.05E-12 |
| IL2RB | -1.073026424 | 5.508774404 | -7.744766701 | 2.07E-12 |
| ADIRF | 1.341583409 | 7.697660376 | 7.690302579 | 2.79E-12 |
| ITGAL | -1.078615461 | 6.285330619 | -7.632363453 | 3.81E-12 |
| RAC2 | -1.069608191 | 7.524808423 | -7.565038191 | 5.49E-12 |
| ITGB2 | -1.064907817 | 7.179905839 | -7.544777706 | 6.12E-12 |
| HIST1H2BM | -1.201510986 | 5.693603463 | -7.419053669 | 1.20E-11 |
| AQP1 | 1.084445477 | 7.10520768 | 7.411446999 | 1.25E-11 |
| IL2RG | -1.420319693 | 8.248275528 | -7.253633031 | 2.90E-11 |
| CD2 | -1.289019526 | 4.631965591 | -7.118815926 | 5.92E-11 |
| BCL2A1 | -1.521507454 | 4.317298752 | -7.085386126 | 7.06E-11 |
| HIST1H2BO | -1.001380472 | 3.881203271 | -7.075063097 | 7.46E-11 |
| PLIN1 | 2.046768117 | 5.343827656 | 7.066947238 | 7.78E-11 |
| TBC1D10C | -1.062345036 | 6.939724303 | -7.05902718 | 8.11E-11 |
| CORO1A | -1.194434124 | 8.132918758 | -7.058083806 | 8.15E-11 |
| CD3D | -1.039548025 | 6.597445345 | -7.056380354 | 8.23E-11 |
| MAOA | 1.42543703 | 5.008358168 | 7.021562704 | 9.87E-11 |
| CAPG | -1.130122724 | 7.453162444 | -7.021229182 | 9.89E-11 |
| SLC7A7 | -1.003842424 | 5.133590326 | -7.016439188 | 1.01E-10 |
| TGFBR3 | 1.105650264 | 4.789062092 | 6.992955549 | 1.15E-10 |
| KLHDC7B | -1.080080638 | 4.912581366 | -6.937981488 | 1.53E-10 |
| SASH3 | -1.13094441 | 6.083612341 | -6.864215449 | 2.24E-10 |
| SCO2 | -1.007783598 | 6.180327065 | -6.848532595 | 2.43E-10 |
| IDO1 | -1.64881183 | 4.718567232 | -6.846050866 | 2.46E-10 |
| CD3G | -1.263865996 | 5.758565671 | -6.840380893 | 2.54E-10 |
| CCL4 | -1.17288646 | 5.912784393 | -6.830209428 | 2.67E-10 |
| AIM2 | -1.384088154 | 5.729166506 | -6.826448951 | 2.73E-10 |
| IL7R | -1.357509402 | 6.035311774 | -6.786817666 | 3.34E-10 |
| SLAMF8 | -1.326966592 | 5.801433062 | -6.76770663 | 3.69E-10 |
| GZMA | -1.002371195 | 5.043128785 | -6.764402256 | 3.75E-10 |
| CIITA | -1.129320126 | 6.181944645 | -6.727508567 | 4.54E-10 |
| RNASE4 | 1.076337856 | 4.799515967 | 6.726028336 | 4.57E-10 |
| PPP1R1A | 1.316612864 | 5.189974981 | 6.682695713 | 5.71E-10 |
| CCL19 | -1.374105214 | 8.327317726 | -6.64386165 | 6.97E-10 |
| PLA2G2D | -1.694514213 | 6.438345745 | -6.632213594 | 7.39E-10 |
| GZMB | -1.255365754 | 3.983156806 | -6.62693594 | 7.60E-10 |
| LAPTM5 | -1.384187152 | 8.769368346 | -6.625970365 | 7.63E-10 |
| SIGLEC10 | -1.015055655 | 5.836623969 | -6.589030035 | 9.21E-10 |
| GPR174 | -1.234913407 | 4.602651297 | -6.58798865 | 9.26E-10 |
| HLA-DMB | -1.02207625 | 8.091899964 | -6.579000861 | 9.70E-10 |
| C2orf40 | 1.194843599 | 3.979899039 | 6.576997385 | 9.80E-10 |
| CDR1 | 1.394289151 | 5.652504678 | 6.569352777 | 1.02E-09 |
| GBP5 | -1.201202048 | 5.385829345 | -6.56809265 | 1.02E-09 |
| CXCL10 | -1.834036743 | 6.262381456 | -6.562345034 | 1.06E-09 |
| ANKRD22 | -1.271591999 | 4.242131175 | -6.461092418 | 1.76E-09 |
| PLEK | -1.0515408 | 6.787503446 | -6.400591871 | 2.39E-09 |
| PBX1 | 1.001750791 | 6.237519413 | 6.379122225 | 2.66E-09 |
| CXCL9 | -2.10053884 | 6.590269557 | -6.336842289 | 3.29E-09 |
| HCK | -1.107619578 | 5.730166405 | -6.27273833 | 4.52E-09 |
| CCR7 | -1.124963689 | 5.384303153 | -6.270888657 | 4.56E-09 |
| CD52 | -1.286746101 | 8.140850365 | -6.242134133 | 5.26E-09 |
| ADH1B | 1.049412257 | 3.643338784 | 6.197246315 | 6.57E-09 |
| GZMK | -1.053832465 | 6.441771795 | -6.192059389 | 6.74E-09 |
| CD3E | -1.291131896 | 5.924818492 | -6.178202626 | 7.22E-09 |
| MYOC | 1.054266718 | 3.917298673 | 6.140892465 | 8.67E-09 |
| RASGRP1 | -1.072846539 | 4.219763491 | -6.13312288 | 9.01E-09 |
| C15orf48 | -1.501861124 | 3.537844123 | -6.080554882 | 1.17E-08 |
| ADAMDEC1 | -1.707258664 | 3.781824883 | -5.996583035 | 1.76E-08 |
| HCLS1 | -1.202797461 | 7.040751333 | -5.987806031 | 1.83E-08 |
| STAT1 | -1.195903582 | 6.950376879 | -5.860749432 | 3.38E-08 |
| IKZF3 | -1.007443158 | 5.696362683 | -5.831767714 | 3.88E-08 |
| FABP4 | 1.228897751 | 4.628201367 | 5.812068604 | 4.27E-08 |
| PLAC8 | -1.134955154 | 5.567820429 | -5.807513712 | 4.36E-08 |
| LCP1 | -1.149072872 | 6.655581177 | -5.802772036 | 4.46E-08 |
| SLAMF7 | -1.104832657 | 6.906352346 | -5.783612672 | 4.89E-08 |
| CXCL13 | -1.896557928 | 5.067793388 | -5.735640476 | 6.14E-08 |
| CECR1 | -1.033719461 | 7.722428643 | -5.712840902 | 6.84E-08 |
| HLA-DRA | -1.209157381 | 10.63095174 | -5.607023988 | 1.12E-07 |
| CXCR4 | -1.39255648 | 7.573882687 | -5.562645132 | 1.38E-07 |
| PLA2G7 | -1.232753545 | 5.313833515 | -5.545203523 | 1.50E-07 |
| CIDEC | 1.237505713 | 5.352159361 | 5.537190835 | 1.56E-07 |
| HSPB6 | 1.478424161 | 6.873662301 | 5.505606528 | 1.80E-07 |
| ADIPOQ | 1.456641103 | 3.211406599 | 5.504697669 | 1.81E-07 |
| IGHM | -1.022939332 | 8.930057021 | -5.495720151 | 1.89E-07 |
| FCGR1B | -1.40339483 | 5.30820961 | -5.468726191 | 2.14E-07 |
| PTPRC | -1.012280978 | 5.12037878 | -5.355734487 | 3.59E-07 |
| APOD | 1.254290444 | 9.376270386 | 5.348436404 | 3.71E-07 |
| PFN2 | 1.06027371 | 6.870603623 | 5.317768955 | 4.27E-07 |
| SELL | -1.367338731 | 6.063727422 | -5.315269924 | 4.31E-07 |
| CHI3L1 | -1.336621115 | 6.046676181 | -5.309886448 | 4.42E-07 |
| GBP1 | -1.091308496 | 4.86072426 | -5.300304896 | 4.62E-07 |
| CCL2 | -1.121022757 | 7.75010096 | -5.300198397 | 4.62E-07 |
| CAV1 | 1.025465745 | 6.40872127 | 5.123795928 | 1.02E-06 |
| FCER1G | -1.240315735 | 7.571183795 | -5.110520438 | 1.08E-06 |
| SCGB2A2 | 1.53974018 | 3.644559047 | 5.004301072 | 1.72E-06 |
| HLA-DPA1 | -1.056672235 | 7.990321178 | -4.993735078 | 1.81E-06 |
| CRYAB | 1.24345001 | 7.614268481 | 4.972975627 | 1.98E-06 |
| AOC3 | 1.009956438 | 3.867466804 | 4.847568772 | 3.40E-06 |
| FHL1 | 1.005465784 | 5.531182989 | 4.83391036 | 3.60E-06 |
| SNX10 | -1.293833627 | 5.159589764 | -4.815539245 | 3.90E-06 |
| FGR | -1.048601298 | 5.765775806 | -4.762077774 | 4.89E-06 |
| SPP1 | -1.221630118 | 4.159506423 | -4.583897446 | 1.03E-05 |
| MMP12 | -1.07975971 | 3.119920723 | -4.26665664 | 3.72E-05 |
| CCL18 | -1.389119275 | 6.420079256 | -4.247376211 | 4.01E-05 |
| TPD52L1 | 1.025320023 | 5.54343024 | 4.146154451 | 5.95E-05 |
| LINC00948 | 1.021203835 | 3.453403485 | 4.12907253 | 6.36E-05 |
| FN1 | -1.027126045 | 7.28403011 | -4.100121717 | 7.11E-05 |
| CHIT1 | -1.03176161 | 5.468413762 | -4.031349239 | 9.24E-05 |
| FDCSP | -1.736338783 | 6.895699682 | -3.909348307 | 0.000146135 |
| C8orf4 | 1.013101416 | 4.168743599 | 3.539841887 | 0.000550585 |
| MS4A1 | -1.024623248 | 5.076686494 | -3.41794406 | 0.000835421 |
| SCGB1D1 | 2.116327844 | 5.680889576 | 3.276151203 | 0.001339021 |
| SCGB2A1 | 1.90766874 | 6.120845492 | 2.922366855 | 0.004076946 |
| LPO | 1.013859842 | 5.768748278 | 2.83090861 | 0.005355244 |
| STATH | 1.165446039 | 4.301394305 | 2.655601521 | 0.008874187 |

# Appendix 5

**Analysis of enrichment**

**Table S5a. Analysis of GO**.

| ONTOLOGY | ID | Description | GeneRatio | pvalue | qvalue |
| --- | --- | --- | --- | --- | --- |
| BP | GO:0007159 | leukocyte cell-cell adhesion | 25/125 | 4.17E-17 | 8.14E-14 |
| BP | GO:0050863 | regulation of T cell activation | 22/125 | 7.59E-15 | 7.41E-12 |
| BP | GO:0050900 | leukocyte migration | 22/125 | 1.78E-14 | 1.16E-11 |
| BP | GO:1903037 | regulation of leukocyte cell-cell adhesion | 21/125 | 8.66E-14 | 4.23E-11 |
| BP | GO:0022407 | regulation of cell-cell adhesion | 23/125 | 1.84E-13 | 5.41E-11 |
| BP | GO:0019221 | cytokine-mediated signaling pathway | 23/125 | 1.92E-13 | 5.41E-11 |
| BP | GO:0050867 | positive regulation of cell activation | 21/125 | 1.94E-13 | 5.41E-11 |
| BP | GO:1903039 | positive regulation of leukocyte cell-cell adhesion | 18/125 | 3.31E-13 | 8.08E-11 |
| BP | GO:0022409 | positive regulation of cell-cell adhesion | 19/125 | 4.77E-13 | 1.03E-10 |
| BP | GO:0030595 | leukocyte chemotaxis | 17/125 | 5.30E-13 | 1.03E-10 |
| BP | GO:0002696 | positive regulation of leukocyte activation | 20/125 | 8.41E-13 | 1.46E-10 |
| BP | GO:0050870 | positive regulation of T cell activation | 17/125 | 8.99E-13 | 1.46E-10 |
| BP | GO:0045785 | positive regulation of cell adhesion | 22/125 | 1.10E-12 | 1.65E-10 |
| BP | GO:0002443 | leukocyte mediated immunity | 20/125 | 2.61E-12 | 3.64E-10 |
| BP | GO:1990266 | neutrophil migration | 13/125 | 3.30E-12 | 4.29E-10 |
| BP | GO:0030593 | neutrophil chemotaxis | 12/125 | 7.11E-12 | 8.67E-10 |
| BP | GO:0002449 | lymphocyte mediated immunity | 17/125 | 1.77E-11 | 2.04E-09 |
| BP | GO:0060326 | cell chemotaxis | 17/125 | 3.29E-11 | 3.57E-09 |
| BP | GO:0097530 | granulocyte migration | 13/125 | 4.15E-11 | 4.26E-09 |
| BP | GO:0051251 | positive regulation of lymphocyte activation | 17/125 | 6.87E-11 | 6.70E-09 |
| BP | GO:0071621 | granulocyte chemotaxis | 12/125 | 7.95E-11 | 7.39E-09 |
| BP | GO:0071674 | mononuclear cell migration | 14/125 | 8.51E-11 | 7.55E-09 |
| BP | GO:0070098 | chemokine-mediated signaling pathway | 10/125 | 5.26E-10 | 4.46E-08 |
| BP | GO:0072676 | lymphocyte migration | 11/125 | 6.00E-10 | 4.88E-08 |
| BP | GO:0097529 | myeloid leukocyte migration | 14/125 | 8.74E-10 | 6.82E-08 |
| BP | GO:1990868 | response to chemokine | 10/125 | 1.22E-09 | 8.81E-08 |
| BP | GO:1990869 | cellular response to chemokine | 10/125 | 1.22E-09 | 8.81E-08 |
| BP | GO:0034341 | response to type II interferon | 11/125 | 2.43E-09 | 1.69E-07 |
| BP | GO:1903131 | mononuclear cell differentiation | 18/125 | 2.87E-09 | 1.93E-07 |
| BP | GO:0030098 | lymphocyte differentiation | 17/125 | 3.41E-09 | 2.22E-07 |
| BP | GO:0002683 | negative regulation of immune system process | 18/125 | 4.38E-09 | 2.76E-07 |
| BP | GO:0001819 | positive regulation of cytokine production | 18/125 | 4.67E-09 | 2.85E-07 |
| BP | GO:0071675 | regulation of mononuclear cell migration | 10/125 | 9.47E-09 | 5.60E-07 |
| BP | GO:0002688 | regulation of leukocyte chemotaxis | 10/125 | 1.29E-08 | 7.43E-07 |
| BP | GO:0030217 | T cell differentiation | 14/125 | 1.45E-08 | 8.10E-07 |
| BP | GO:0042098 | T cell proliferation | 12/125 | 2.10E-08 | 1.14E-06 |
| BP | GO:0030101 | natural killer cell activation | 9/125 | 2.36E-08 | 1.25E-06 |
| BP | GO:0042742 | defense response to bacterium | 14/125 | 2.58E-08 | 1.32E-06 |
| BP | GO:0046631 | alpha-beta T cell activation | 11/125 | 2.72E-08 | 1.36E-06 |
| BP | GO:0002685 | regulation of leukocyte migration | 12/125 | 4.24E-08 | 2.05E-06 |
| BP | GO:0042129 | regulation of T cell proliferation | 11/125 | 4.31E-08 | 2.05E-06 |
| BP | GO:0050920 | regulation of chemotaxis | 12/125 | 4.90E-08 | 2.28E-06 |
| BP | GO:0071356 | cellular response to tumor necrosis factor | 12/125 | 6.20E-08 | 2.82E-06 |
| BP | GO:0071346 | cellular response to type II interferon | 9/125 | 8.66E-08 | 3.84E-06 |
| BP | GO:0002366 | leukocyte activation involved in immune response | 13/125 | 9.97E-08 | 4.17E-06 |
| BP | GO:0032609 | type II interferon production | 9/125 | 1.00E-07 | 4.17E-06 |
| BP | GO:0032649 | regulation of type II interferon production | 9/125 | 1.00E-07 | 4.17E-06 |
| BP | GO:0002263 | cell activation involved in immune response | 13/125 | 1.16E-07 | 4.74E-06 |
| BP | GO:0050727 | regulation of inflammatory response | 15/125 | 1.22E-07 | 4.87E-06 |
| BP | GO:0046651 | lymphocyte proliferation | 13/125 | 1.46E-07 | 5.72E-06 |
| BP | GO:0034612 | response to tumor necrosis factor | 12/125 | 1.64E-07 | 6.29E-06 |
| BP | GO:0002460 | adaptive immune response based on somatic recombination of immune receptors built from immunoglobulin superfamily domains | 13/125 | 1.76E-07 | 6.62E-06 |
| BP | GO:0032943 | mononuclear cell proliferation | 13/125 | 1.90E-07 | 7.00E-06 |
| BP | GO:0048247 | lymphocyte chemotaxis | 7/125 | 2.35E-07 | 8.50E-06 |
| BP | GO:0002690 | positive regulation of leukocyte chemotaxis | 8/125 | 2.67E-07 | 9.47E-06 |
| BP | GO:0002768 | immune response-regulating cell surface receptor signaling pathway | 13/125 | 3.36E-07 | 1.17E-05 |
| BP | GO:0002764 | immune response-regulating signaling pathway | 15/125 | 3.56E-07 | 1.21E-05 |
| BP | GO:2000401 | regulation of lymphocyte migration | 7/125 | 3.59E-07 | 1.21E-05 |
| BP | GO:0001909 | leukocyte mediated cytotoxicity | 9/125 | 4.09E-07 | 1.35E-05 |
| BP | GO:0002548 | monocyte chemotaxis | 7/125 | 4.84E-07 | 1.55E-05 |
| BP | GO:0072678 | T cell migration | 7/125 | 4.84E-07 | 1.55E-05 |
| BP | GO:0050921 | positive regulation of chemotaxis | 9/125 | 5.20E-07 | 1.63E-05 |
| BP | GO:0071677 | positive regulation of mononuclear cell migration | 7/125 | 5.33E-07 | 1.63E-05 |
| BP | GO:0031295 | T cell costimulation | 6/125 | 5.34E-07 | 1.63E-05 |
| BP | GO:0070661 | leukocyte proliferation | 13/125 | 6.35E-07 | 1.88E-05 |
| BP | GO:0050670 | regulation of lymphocyte proliferation | 11/125 | 6.37E-07 | 1.88E-05 |
| BP | GO:0002687 | positive regulation of leukocyte migration | 9/125 | 6.56E-07 | 1.91E-05 |
| BP | GO:0031294 | lymphocyte costimulation | 6/125 | 6.97E-07 | 2.00E-05 |
| BP | GO:1901623 | regulation of lymphocyte chemotaxis | 5/125 | 7.40E-07 | 2.09E-05 |
| BP | GO:0032944 | regulation of mononuclear cell proliferation | 11/125 | 7.50E-07 | 2.09E-05 |
| BP | GO:0042267 | natural killer cell mediated cytotoxicity | 7/125 | 7.74E-07 | 2.13E-05 |
| BP | GO:0001906 | cell killing | 10/125 | 8.66E-07 | 2.35E-05 |
| BP | GO:0002429 | immune response-activating cell surface receptor signaling pathway | 12/125 | 9.20E-07 | 2.46E-05 |
| BP | GO:0002757 | immune response-activating signaling pathway | 14/125 | 9.77E-07 | 2.58E-05 |
| BP | GO:0019882 | antigen processing and presentation | 8/125 | 1.00E-06 | 2.59E-05 |
| BP | GO:0002228 | natural killer cell mediated immunity | 7/125 | 1.01E-06 | 2.59E-05 |
| BP | GO:0002253 | activation of immune response | 15/125 | 1.18E-06 | 2.99E-05 |
| BP | GO:0043299 | leukocyte degranulation | 7/125 | 1.20E-06 | 3.00E-05 |
| BP | GO:1902105 | regulation of leukocyte differentiation | 12/125 | 1.63E-06 | 4.03E-05 |
| BP | GO:0033077 | T cell differentiation in thymus | 7/125 | 1.80E-06 | 4.39E-05 |
| BP | GO:0019886 | antigen processing and presentation of exogenous peptide antigen via MHC class II | 5/125 | 1.86E-06 | 4.49E-05 |
| BP | GO:0070663 | regulation of leukocyte proliferation | 11/125 | 1.95E-06 | 4.65E-05 |
| BP | GO:2000107 | negative regulation of leukocyte apoptotic process | 6/125 | 2.01E-06 | 4.72E-05 |
| BP | GO:2000106 | regulation of leukocyte apoptotic process | 7/125 | 2.45E-06 | 5.69E-05 |
| BP | GO:0070371 | ERK1 and ERK2 cascade | 12/125 | 3.25E-06 | 7.43E-05 |
| BP | GO:0002399 | MHC class II protein complex assembly | 4/125 | 3.31E-06 | 7.43E-05 |
| BP | GO:0002503 | peptide antigen assembly with MHC class II protein complex | 4/125 | 3.31E-06 | 7.43E-05 |
| BP | GO:0002495 | antigen processing and presentation of peptide antigen via MHC class II | 5/125 | 3.48E-06 | 7.72E-05 |
| BP | GO:0032496 | response to lipopolysaccharide | 12/125 | 3.67E-06 | 8.04E-05 |
| BP | GO:0006909 | phagocytosis | 10/125 | 4.40E-06 | 9.55E-05 |
| BP | GO:1903706 | regulation of hemopoiesis | 13/125 | 4.45E-06 | 9.55E-05 |
| BP | GO:0002504 | antigen processing and presentation of peptide or polysaccharide antigen via MHC class II | 5/125 | 4.62E-06 | 9.81E-05 |
| BP | GO:0050852 | T cell receptor signaling pathway | 8/125 | 4.70E-06 | 9.86E-05 |
| BP | GO:0032535 | regulation of cellular component size | 12/125 | 4.77E-06 | 9.90E-05 |
| BP | GO:0070555 | response to interleukin-1 | 8/125 | 4.96E-06 | 0.000101809 |
| BP | GO:0050764 | regulation of phagocytosis | 7/125 | 5.30E-06 | 0.000107811 |
| BP | GO:0031589 | cell-substrate adhesion | 12/125 | 5.50E-06 | 0.000110692 |
| BP | GO:0016064 | immunoglobulin mediated immune response | 8/125 | 6.10E-06 | 0.000121406 |
| BP | GO:0002237 | response to molecule of bacterial origin | 12/125 | 6.69E-06 | 0.000131915 |
| BP | GO:0042102 | positive regulation of T cell proliferation | 7/125 | 6.86E-06 | 0.000132729 |
| BP | GO:0002478 | antigen processing and presentation of exogenous peptide antigen | 5/125 | 6.87E-06 | 0.000132729 |
| BP | GO:0019724 | B cell mediated immunity | 8/125 | 7.09E-06 | 0.000135675 |
| BP | GO:0032941 | secretion by tissue | 5/125 | 7.78E-06 | 0.000146017 |
| BP | GO:2000403 | positive regulation of lymphocyte migration | 5/125 | 7.78E-06 | 0.000146017 |
| BP | GO:0048002 | antigen processing and presentation of peptide antigen | 6/125 | 8.15E-06 | 0.000151447 |
| BP | GO:0007162 | negative regulation of cell adhesion | 11/125 | 8.36E-06 | 0.00015399 |
| BP | GO:0002396 | MHC protein complex assembly | 4/125 | 8.64E-06 | 0.000156115 |
| BP | GO:0002501 | peptide antigen assembly with MHC protein complex | 4/125 | 8.64E-06 | 0.000156115 |
| BP | GO:0002695 | negative regulation of leukocyte activation | 9/125 | 9.02E-06 | 0.000161509 |
| BP | GO:0070372 | regulation of ERK1 and ERK2 cascade | 11/125 | 9.15E-06 | 0.000162375 |
| BP | GO:0071347 | cellular response to interleukin-1 | 7/125 | 9.91E-06 | 0.000174201 |
| BP | GO:0007229 | integrin-mediated signaling pathway | 7/125 | 1.05E-05 | 0.000181517 |
| BP | GO:0014066 | regulation of phosphatidylinositol 3-kinase signaling | 7/125 | 1.05E-05 | 0.000181517 |
| BP | GO:0002697 | regulation of immune effector process | 12/125 | 1.08E-05 | 0.000185716 |
| BP | GO:2000516 | positive regulation of CD4-positive, alpha-beta T cell activation | 5/125 | 1.11E-05 | 0.000188308 |
| BP | GO:0002285 | lymphocyte activation involved in immune response | 9/125 | 1.14E-05 | 0.000191976 |
| BP | GO:0006816 | calcium ion transport | 13/125 | 1.20E-05 | 0.000199379 |
| BP | GO:0035710 | CD4-positive, alpha-beta T cell activation | 7/125 | 1.25E-05 | 0.000206761 |
| BP | GO:0045061 | thymic T cell selection | 4/125 | 1.29E-05 | 0.000210539 |
| BP | GO:0002699 | positive regulation of immune effector process | 10/125 | 1.30E-05 | 0.000210539 |
| BP | GO:0032729 | positive regulation of type II interferon production | 6/125 | 1.31E-05 | 0.000210539 |
| BP | GO:0045619 | regulation of lymphocyte differentiation | 9/125 | 1.33E-05 | 0.000212573 |
| BP | GO:0071887 | leukocyte apoptotic process | 7/125 | 1.40E-05 | 0.000222055 |
| BP | GO:0072243 | metanephric nephron epithelium development | 4/125 | 1.55E-05 | 0.000244082 |
| BP | GO:0002286 | T cell activation involved in immune response | 7/125 | 1.56E-05 | 0.000244082 |
| BP | GO:0070665 | positive regulation of leukocyte proliferation | 8/125 | 1.64E-05 | 0.000254138 |
| BP | GO:2000404 | regulation of T cell migration | 5/125 | 1.71E-05 | 0.000263143 |
| BP | GO:0051250 | negative regulation of lymphocyte activation | 8/125 | 1.79E-05 | 0.00027283 |
| BP | GO:0034695 | response to prostaglandin E | 4/125 | 1.86E-05 | 0.000280751 |
| BP | GO:0050848 | regulation of calcium-mediated signaling | 6/125 | 1.88E-05 | 0.000281612 |
| BP | GO:0019884 | antigen processing and presentation of exogenous antigen | 5/125 | 1.90E-05 | 0.000282579 |
| BP | GO:0010810 | regulation of cell-substrate adhesion | 9/125 | 1.92E-05 | 0.000283463 |
| BP | GO:0009615 | response to virus | 12/125 | 1.98E-05 | 0.000290729 |
| BP | GO:0014068 | positive regulation of phosphatidylinositol 3-kinase signaling | 6/125 | 2.01E-05 | 0.000292889 |
| BP | GO:0043254 | regulation of protein-containing complex assembly | 12/125 | 2.03E-05 | 0.000293419 |
| BP | GO:0050866 | negative regulation of cell activation | 9/125 | 2.13E-05 | 0.000305951 |
| BP | GO:0045055 | regulated exocytosis | 9/125 | 2.37E-05 | 0.000337188 |
| BP | GO:0002407 | dendritic cell chemotaxis | 4/125 | 2.58E-05 | 0.000365418 |
| BP | GO:0090066 | regulation of anatomical structure size | 13/125 | 2.72E-05 | 0.00038126 |
| BP | GO:0051209 | release of sequestered calcium ion into cytosol | 7/125 | 2.77E-05 | 0.000386703 |
| BP | GO:0051651 | maintenance of location in cell | 9/125 | 2.81E-05 | 0.000388592 |
| BP | GO:0051283 | negative regulation of sequestering of calcium ion | 7/125 | 2.91E-05 | 0.000400557 |
| BP | GO:0007589 | body fluid secretion | 6/125 | 3.00E-05 | 0.000408915 |
| BP | GO:0072207 | metanephric epithelium development | 4/125 | 3.02E-05 | 0.000408966 |
| BP | GO:0051282 | regulation of sequestering of calcium ion | 7/125 | 3.21E-05 | 0.000432439 |
| BP | GO:0031529 | ruffle organization | 5/125 | 3.35E-05 | 0.000447911 |
| BP | GO:0007160 | cell-matrix adhesion | 9/125 | 3.43E-05 | 0.000448951 |
| BP | GO:0002291 | T cell activation via T cell receptor contact with antigen bound to MHC molecule on antigen presenting cell | 3/125 | 3.43E-05 | 0.000448951 |
| BP | GO:0002361 | CD4-positive, CD25-positive, alpha-beta regulatory T cell differentiation | 3/125 | 3.43E-05 | 0.000448951 |
| BP | GO:0051208 | sequestering of calcium ion | 7/125 | 3.89E-05 | 0.000505528 |
| BP | GO:0042060 | wound healing | 12/125 | 4.05E-05 | 0.000522636 |
| BP | GO:0008360 | regulation of cell shape | 7/125 | 4.07E-05 | 0.000522636 |
| BP | GO:0031349 | positive regulation of defense response | 12/125 | 4.23E-05 | 0.000540052 |
| BP | GO:0007015 | actin filament organization | 12/125 | 4.33E-05 | 0.000548414 |
| BP | GO:0033089 | positive regulation of T cell differentiation in thymus | 3/125 | 4.69E-05 | 0.0005905 |
| BP | GO:0032102 | negative regulation of response to external stimulus | 12/125 | 4.72E-05 | 0.000590528 |
| BP | GO:0014065 | phosphatidylinositol 3-kinase signaling | 7/125 | 5.34E-05 | 0.000663685 |
| BP | GO:0050671 | positive regulation of lymphocyte proliferation | 7/125 | 5.58E-05 | 0.000689117 |
| BP | GO:0034694 | response to prostaglandin | 4/125 | 6.02E-05 | 0.00073947 |
| BP | GO:0097048 | dendritic cell apoptotic process | 3/125 | 6.22E-05 | 0.000754273 |
| BP | GO:2000668 | regulation of dendritic cell apoptotic process | 3/125 | 6.22E-05 | 0.000754273 |
| BP | GO:0032946 | positive regulation of mononuclear cell proliferation | 7/125 | 6.35E-05 | 0.000764982 |
| BP | GO:0050851 | antigen receptor-mediated signaling pathway | 8/125 | 6.43E-05 | 0.000764982 |
| BP | GO:0097553 | calcium ion transmembrane import into cytosol | 8/125 | 6.43E-05 | 0.000764982 |
| BP | GO:0008064 | regulation of actin polymerization or depolymerization | 7/125 | 6.63E-05 | 0.000784031 |
| BP | GO:0036336 | dendritic cell migration | 4/125 | 6.82E-05 | 0.00079701 |
| BP | GO:0038094 | Fc-gamma receptor signaling pathway | 4/125 | 6.82E-05 | 0.00079701 |
| BP | GO:0032615 | interleukin-12 production | 5/125 | 7.00E-05 | 0.000808191 |
| BP | GO:0032655 | regulation of interleukin-12 production | 5/125 | 7.00E-05 | 0.000808191 |
| BP | GO:0001894 | tissue homeostasis | 9/125 | 7.19E-05 | 0.000820183 |
| BP | GO:0060249 | anatomical structure homeostasis | 9/125 | 7.19E-05 | 0.000820183 |
| BP | GO:0055074 | calcium ion homeostasis | 10/125 | 7.26E-05 | 0.000824244 |
| BP | GO:0030832 | regulation of actin filament length | 7/125 | 7.52E-05 | 0.000848041 |
| BP | GO:0043372 | positive regulation of CD4-positive, alpha-beta T cell differentiation | 4/125 | 7.69E-05 | 0.000857524 |
| BP | GO:2000406 | positive regulation of T cell migration | 4/125 | 7.69E-05 | 0.000857524 |
| BP | GO:0051924 | regulation of calcium ion transport | 9/125 | 7.84E-05 | 0.000869184 |
| BP | GO:0050729 | positive regulation of inflammatory response | 7/125 | 8.16E-05 | 0.000895696 |
| BP | GO:1901654 | response to ketone | 8/125 | 8.17E-05 | 0.000895696 |
| BP | GO:0110053 | regulation of actin filament organization | 9/125 | 8.30E-05 | 0.000904963 |
| BP | GO:0052548 | regulation of endopeptidase activity | 10/125 | 9.09E-05 | 0.000985997 |
| BP | GO:0043547 | positive regulation of GTPase activity | 9/125 | 9.29E-05 | 0.001001972 |
| BP | GO:0045059 | positive thymic T cell selection | 3/125 | 0.000101948 | 0.001093183 |
| BP | GO:0033280 | response to vitamin D | 4/125 | 0.000107817 | 0.001149802 |
| BP | GO:0046634 | regulation of alpha-beta T cell activation | 6/125 | 0.000119973 | 0.001272486 |
| BP | GO:0046635 | positive regulation of alpha-beta T cell activation | 5/125 | 0.00012305 | 0.001298065 |
| BP | GO:0002468 | dendritic cell antigen processing and presentation | 3/125 | 0.00012681 | 0.001330539 |
| BP | GO:0045582 | positive regulation of T cell differentiation | 6/125 | 0.000132034 | 0.001377338 |
| BP | GO:0045730 | respiratory burst | 4/125 | 0.000132888 | 0.001377338 |
| BP | GO:0010959 | regulation of metal ion transport | 11/125 | 0.000133388 | 0.001377338 |
| BP | GO:0071222 | cellular response to lipopolysaccharide | 8/125 | 0.000136536 | 0.00140242 |
| BP | GO:0052547 | regulation of peptidase activity | 11/125 | 0.000139003 | 0.001420291 |
| BP | GO:0002573 | myeloid leukocyte differentiation | 8/125 | 0.000149606 | 0.001520664 |
| BP | GO:0010819 | regulation of T cell chemotaxis | 3/125 | 0.00015531 | 0.00157046 |
| BP | GO:0019932 | second-messenger-mediated signaling | 9/125 | 0.000158797 | 0.001597447 |
| BP | GO:0072210 | metanephric nephron development | 4/125 | 0.000161929 | 0.001618855 |
| BP | GO:0048015 | phosphatidylinositol-mediated signaling | 7/125 | 0.000162584 | 0.001618855 |
| BP | GO:0050878 | regulation of body fluid levels | 10/125 | 0.000163491 | 0.001619627 |
| BP | GO:0031214 | biomineral tissue development | 7/125 | 0.000168507 | 0.001650838 |
| BP | GO:0043270 | positive regulation of monoatomic ion transport | 8/125 | 0.000168622 | 0.001650838 |
| BP | GO:2000514 | regulation of CD4-positive, alpha-beta T cell activation | 5/125 | 0.00016918 | 0.001650838 |
| BP | GO:0050691 | regulation of defense response to virus by host | 4/125 | 0.00017805 | 0.001728745 |
| BP | GO:0002437 | inflammatory response to antigenic stimulus | 5/125 | 0.000179804 | 0.001737137 |
| BP | GO:2000116 | regulation of cysteine-type endopeptidase activity | 8/125 | 0.000184155 | 0.001770414 |
| BP | GO:0048017 | inositol lipid-mediated signaling | 7/125 | 0.000187334 | 0.001792147 |
| BP | GO:0032735 | positive regulation of interleukin-12 production | 4/125 | 0.000195297 | 0.001859206 |
| BP | GO:0001952 | regulation of cell-matrix adhesion | 6/125 | 0.000198734 | 0.001880291 |
| BP | GO:0018108 | peptidyl-tyrosine phosphorylation | 10/125 | 0.000199439 | 0.001880291 |
| BP | GO:0071219 | cellular response to molecule of bacterial origin | 8/125 | 0.000200847 | 0.001884468 |
| BP | GO:0018212 | peptidyl-tyrosine modification | 10/125 | 0.000208271 | 0.00194477 |
| BP | GO:0050856 | regulation of T cell receptor signaling pathway | 4/125 | 0.000213717 | 0.001979628 |
| BP | GO:0048872 | homeostasis of number of cells | 9/125 | 0.000214406 | 0.001979628 |
| BP | GO:0045580 | regulation of T cell differentiation | 7/125 | 0.000215047 | 0.001979628 |
| BP | GO:0002274 | myeloid leukocyte activation | 8/125 | 0.000218763 | 0.002004384 |
| BP | GO:1902903 | regulation of supramolecular fiber organization | 10/125 | 0.000226925 | 0.00206945 |
| BP | GO:1902622 | regulation of neutrophil migration | 4/125 | 0.000233356 | 0.002118198 |
| BP | GO:0051928 | positive regulation of calcium ion transport | 6/125 | 0.000235806 | 0.002130528 |
| BP | GO:0030833 | regulation of actin filament polymerization | 6/125 | 0.000245874 | 0.00217216 |
| BP | GO:0045621 | positive regulation of lymphocyte differentiation | 6/125 | 0.000245874 | 0.00217216 |
| BP | GO:0050864 | regulation of B cell activation | 6/125 | 0.000245874 | 0.00217216 |
| BP | GO:1902107 | positive regulation of leukocyte differentiation | 7/125 | 0.000245979 | 0.00217216 |
| BP | GO:1903708 | positive regulation of hemopoiesis | 7/125 | 0.000245979 | 0.00217216 |
| BP | GO:0022604 | regulation of cell morphogenesis | 8/125 | 0.000251529 | 0.002211166 |
| BP | GO:0002703 | regulation of leukocyte mediated immunity | 8/125 | 0.000258542 | 0.00226262 |
| BP | GO:0019722 | calcium-mediated signaling | 7/125 | 0.000271453 | 0.002365007 |
| BP | GO:0002861 | regulation of inflammatory response to antigenic stimulus | 4/125 | 0.000276478 | 0.002398084 |
| BP | GO:0032970 | regulation of actin filament-based process | 10/125 | 0.000285608 | 0.002466313 |
| BP | GO:0002831 | regulation of response to biotic stimulus | 11/125 | 0.00029095 | 0.00250137 |
| BP | GO:0051495 | positive regulation of cytoskeleton organization | 7/125 | 0.000299009 | 0.002559381 |
| BP | GO:0002544 | chronic inflammatory response | 3/125 | 0.00031002 | 0.002619167 |
| BP | GO:0072234 | metanephric nephron tubule development | 3/125 | 0.00031002 | 0.002619167 |
| BP | GO:0140131 | positive regulation of lymphocyte chemotaxis | 3/125 | 0.00031002 | 0.002619167 |
| BP | GO:0008154 | actin polymerization or depolymerization | 7/125 | 0.000318596 | 0.002680026 |
| BP | GO:0043367 | CD4-positive, alpha-beta T cell differentiation | 5/125 | 0.000333357 | 0.00279216 |
| BP | GO:0031334 | positive regulation of protein-containing complex assembly | 7/125 | 0.000349898 | 0.002918177 |
| BP | GO:0007586 | digestion | 6/125 | 0.000352538 | 0.002927681 |
| BP | GO:0071731 | response to nitric oxide | 3/125 | 0.00035992 | 0.002976323 |
| BP | GO:0045058 | T cell selection | 4/125 | 0.000379429 | 0.003124412 |
| BP | GO:0046638 | positive regulation of alpha-beta T cell differentiation | 4/125 | 0.000408928 | 0.003353174 |
| BP | GO:0071216 | cellular response to biotic stimulus | 8/125 | 0.000414812 | 0.003387192 |
| BP | GO:0072073 | kidney epithelium development | 6/125 | 0.00045815 | 0.003725486 |
| BP | GO:0030857 | negative regulation of epithelial cell differentiation | 4/125 | 0.000472781 | 0.00379587 |
| BP | GO:0038093 | Fc receptor signaling pathway | 4/125 | 0.000472781 | 0.00379587 |
| BP | GO:0019835 | cytolysis | 3/125 | 0.000474586 | 0.00379587 |
| BP | GO:0072170 | metanephric tubule development | 3/125 | 0.000474586 | 0.00379587 |
| BP | GO:0043370 | regulation of CD4-positive, alpha-beta T cell differentiation | 4/125 | 0.000507234 | 0.004040439 |
| BP | GO:0002275 | myeloid cell activation involved in immune response | 5/125 | 0.000519851 | 0.004107407 |
| BP | GO:0120162 | positive regulation of cold-induced thermogenesis | 5/125 | 0.000519851 | 0.004107407 |
| BP | GO:0019216 | regulation of lipid metabolic process | 9/125 | 0.000528819 | 0.004161419 |
| BP | GO:0071676 | negative regulation of mononuclear cell migration | 3/125 | 0.000539733 | 0.004208572 |
| BP | GO:0051346 | negative regulation of hydrolase activity | 9/125 | 0.000539957 | 0.004208572 |
| BP | GO:0001954 | positive regulation of cell-matrix adhesion | 4/125 | 0.000543437 | 0.004208572 |
| BP | GO:0045806 | negative regulation of endocytosis | 4/125 | 0.000543437 | 0.004208572 |
| BP | GO:0032956 | regulation of actin cytoskeleton organization | 9/125 | 0.000551286 | 0.004248517 |
| BP | GO:0030099 | myeloid cell differentiation | 10/125 | 0.000552949 | 0.004248517 |
| BP | GO:0042113 | B cell activation | 8/125 | 0.000556384 | 0.004258148 |
| BP | GO:0051235 | maintenance of location | 9/125 | 0.000562811 | 0.004290505 |
| BP | GO:0032693 | negative regulation of interleukin-10 production | 3/125 | 0.000610336 | 0.00460319 |
| BP | GO:0090023 | positive regulation of neutrophil chemotaxis | 3/125 | 0.000610336 | 0.00460319 |
| BP | GO:0006887 | exocytosis | 9/125 | 0.000610903 | 0.00460319 |
| BP | GO:0009612 | response to mechanical stimulus | 7/125 | 0.000627159 | 0.004689463 |
| BP | GO:0043491 | protein kinase B signaling | 7/125 | 0.000627159 | 0.004689463 |
| BP | GO:0007584 | response to nutrient | 6/125 | 0.000650086 | 0.004841875 |
| BP | GO:0019233 | sensory perception of pain | 5/125 | 0.000652504 | 0.004841875 |
| BP | GO:0010720 | positive regulation of cell development | 10/125 | 0.000674653 | 0.004987269 |
| BP | GO:0033622 | integrin activation | 3/125 | 0.000686575 | 0.005037236 |
| BP | GO:0046629 | gamma-delta T cell activation | 3/125 | 0.000686575 | 0.005037236 |
| BP | GO:0010951 | negative regulation of endopeptidase activity | 6/125 | 0.000742298 | 0.005425666 |
| BP | GO:0034113 | heterotypic cell-cell adhesion | 4/125 | 0.000752448 | 0.005479334 |
| BP | GO:0070374 | positive regulation of ERK1 and ERK2 cascade | 7/125 | 0.000757558 | 0.005496039 |
| BP | GO:0010818 | T cell chemotaxis | 3/125 | 0.000768624 | 0.005514821 |
| BP | GO:0032753 | positive regulation of interleukin-4 production | 3/125 | 0.000768624 | 0.005514821 |
| BP | GO:0033081 | regulation of T cell differentiation in thymus | 3/125 | 0.000768624 | 0.005514821 |
| BP | GO:0022600 | digestive system process | 5/125 | 0.000775692 | 0.005524904 |
| BP | GO:0062014 | negative regulation of small molecule metabolic process | 5/125 | 0.000775692 | 0.005524904 |
| BP | GO:0043087 | regulation of GTPase activity | 9/125 | 0.000791021 | 0.005600467 |
| BP | GO:0030041 | actin filament polymerization | 6/125 | 0.00079204 | 0.005600467 |
| BP | GO:0033157 | regulation of intracellular protein transport | 7/125 | 0.000798505 | 0.005617461 |
| BP | GO:0032233 | positive regulation of actin filament bundle assembly | 4/125 | 0.0008002 | 0.005617461 |
| BP | GO:0032623 | interleukin-2 production | 4/125 | 0.000850054 | 0.00592481 |
| BP | GO:0032663 | regulation of interleukin-2 production | 4/125 | 0.000850054 | 0.00592481 |
| BP | GO:0070269 | pyroptosis | 3/125 | 0.000856657 | 0.005928486 |
| BP | GO:0071624 | positive regulation of granulocyte chemotaxis | 3/125 | 0.000856657 | 0.005928486 |
| BP | GO:0001818 | negative regulation of cytokine production | 9/125 | 0.000904651 | 0.006238509 |
| BP | GO:0045088 | regulation of innate immune response | 9/125 | 0.00093945 | 0.006455669 |
| BP | GO:0048245 | eosinophil chemotaxis | 3/125 | 0.000950838 | 0.006488237 |
| BP | GO:0090025 | regulation of monocyte chemotaxis | 3/125 | 0.000950838 | 0.006488237 |
| BP | GO:0050854 | regulation of antigen receptor-mediated signaling pathway | 4/125 | 0.000956264 | 0.006502527 |
| BP | GO:0010812 | negative regulation of cell-substrate adhesion | 4/125 | 0.001012723 | 0.0068152 |
| BP | GO:0032613 | interleukin-10 production | 4/125 | 0.001012723 | 0.0068152 |
| BP | GO:0032653 | regulation of interleukin-10 production | 4/125 | 0.001012723 | 0.0068152 |
| BP | GO:0006874 | intracellular calcium ion homeostasis | 8/125 | 0.001019863 | 0.00681624 |
| BP | GO:0051607 | defense response to virus | 8/125 | 0.001019863 | 0.00681624 |
| BP | GO:0002526 | acute inflammatory response | 5/125 | 0.001032004 | 0.00687385 |
| BP | GO:0140546 | defense response to symbiont | 8/125 | 0.001041632 | 0.006914375 |
| BP | GO:1902905 | positive regulation of supramolecular fiber organization | 6/125 | 0.00104855 | 0.006936704 |
| BP | GO:0034764 | positive regulation of transmembrane transport | 7/125 | 0.001056068 | 0.006962838 |
| BP | GO:0051403 | stress-activated MAPK cascade | 7/125 | 0.001109214 | 0.007288612 |
| BP | GO:0072009 | nephron epithelium development | 5/125 | 0.001115586 | 0.007305886 |
| BP | GO:0002230 | positive regulation of defense response to virus by host | 3/125 | 0.001158297 | 0.007535029 |
| BP | GO:0048873 | homeostasis of number of cells within a tissue | 3/125 | 0.001158297 | 0.007535029 |
| BP | GO:0034767 | positive regulation of monoatomic ion transmembrane transport | 6/125 | 0.001181163 | 0.00765825 |
| BP | GO:0051897 | positive regulation of protein kinase B signaling | 5/125 | 0.001204084 | 0.007781013 |
| BP | GO:0030278 | regulation of ossification | 5/125 | 0.001250233 | 0.008011765 |
| BP | GO:0046632 | alpha-beta T cell differentiation | 5/125 | 0.001250233 | 0.008011765 |
| BP | GO:0050680 | negative regulation of epithelial cell proliferation | 6/125 | 0.001252108 | 0.008011765 |
| BP | GO:0050922 | negative regulation of chemotaxis | 4/125 | 0.001262087 | 0.008049222 |
| BP | GO:0002431 | Fc receptor mediated stimulatory signaling pathway | 3/125 | 0.001271889 | 0.008059063 |
| BP | GO:0043552 | positive regulation of phosphatidylinositol 3-kinase activity | 3/125 | 0.001271889 | 0.008059063 |
| BP | GO:0008361 | regulation of cell size | 6/125 | 0.001288782 | 0.008139674 |
| BP | GO:0031098 | stress-activated protein kinase signaling cascade | 7/125 | 0.001343169 | 0.008449125 |
| BP | GO:0030282 | bone mineralization | 5/125 | 0.001346437 | 0.008449125 |
| BP | GO:0072677 | eosinophil migration | 3/125 | 0.001392258 | 0.008680833 |
| BP | GO:0090183 | regulation of kidney development | 3/125 | 0.001392258 | 0.008680833 |
| BP | GO:0002260 | lymphocyte homeostasis | 4/125 | 0.001401593 | 0.008683551 |
| BP | GO:0042130 | negative regulation of T cell proliferation | 4/125 | 0.001401593 | 0.008683551 |
| BP | GO:0046637 | regulation of alpha-beta T cell differentiation | 4/125 | 0.001475227 | 0.00908209 |
| BP | GO:0050766 | positive regulation of phagocytosis | 4/125 | 0.001475227 | 0.00908209 |
| BP | GO:0006959 | humoral immune response | 7/125 | 0.00150793 | 0.0092097 |
| BP | GO:0045577 | regulation of B cell differentiation | 3/125 | 0.001519551 | 0.0092097 |
| BP | GO:0050869 | negative regulation of B cell activation | 3/125 | 0.001519551 | 0.0092097 |
| BP | GO:0090022 | regulation of neutrophil chemotaxis | 3/125 | 0.001519551 | 0.0092097 |
| BP | GO:1902624 | positive regulation of neutrophil migration | 3/125 | 0.001519551 | 0.0092097 |
| BP | GO:0010811 | positive regulation of cell-substrate adhesion | 5/125 | 0.001555103 | 0.009389516 |
| BP | GO:0051051 | negative regulation of transport | 10/125 | 0.001564225 | 0.009389516 |
| BP | GO:0032640 | tumor necrosis factor production | 6/125 | 0.001569029 | 0.009389516 |
| BP | GO:0032680 | regulation of tumor necrosis factor production | 6/125 | 0.001569029 | 0.009389516 |
| BP | GO:0045926 | negative regulation of growth | 7/125 | 0.001578087 | 0.009389516 |
| BP | GO:0097305 | response to alcohol | 7/125 | 0.001578087 | 0.009389516 |
| BP | GO:0043405 | regulation of MAP kinase activity | 6/125 | 0.001612572 | 0.009565535 |
| BP | GO:0002381 | immunoglobulin production involved in immunoglobulin-mediated immune response | 4/125 | 0.001630511 | 0.009642636 |
| BP | GO:0032633 | interleukin-4 production | 3/125 | 0.001653912 | 0.00969291 |
| BP | GO:0032673 | regulation of interleukin-4 production | 3/125 | 0.001653912 | 0.00969291 |
| BP | GO:0051354 | negative regulation of oxidoreductase activity | 3/125 | 0.001653912 | 0.00969291 |
| BP | GO:0050730 | regulation of peptidyl-tyrosine phosphorylation | 7/125 | 0.001688068 | 0.009863465 |
| BP | GO:0006898 | receptor-mediated endocytosis | 7/125 | 0.001726024 | 0.010055142 |
| BP | GO:0032874 | positive regulation of stress-activated MAPK cascade | 5/125 | 0.001786647 | 0.010339384 |
| BP | GO:0090218 | positive regulation of lipid kinase activity | 3/125 | 0.00179548 | 0.010339384 |
| BP | GO:0071706 | tumor necrosis factor superfamily cytokine production | 6/125 | 0.001796008 | 0.010339384 |
| BP | GO:1903555 | regulation of tumor necrosis factor superfamily cytokine production | 6/125 | 0.001796008 | 0.010339384 |
| BP | GO:0032386 | regulation of intracellular transport | 8/125 | 0.001819055 | 0.010441263 |
| BP | GO:0030308 | negative regulation of cell growth | 6/125 | 0.001844246 | 0.010493268 |
| BP | GO:0032271 | regulation of protein polymerization | 6/125 | 0.001844246 | 0.010493268 |
| BP | GO:0050777 | negative regulation of immune response | 6/125 | 0.001844246 | 0.010493268 |
| BP | GO:0016049 | cell growth | 10/125 | 0.001873527 | 0.010598073 |
| BP | GO:0043410 | positive regulation of MAPK cascade | 10/125 | 0.001873527 | 0.010598073 |
| BP | GO:0050868 | negative regulation of T cell activation | 5/125 | 0.001911452 | 0.010750288 |
| BP | GO:0070304 | positive regulation of stress-activated protein kinase signaling cascade | 5/125 | 0.001911452 | 0.010750288 |
| BP | GO:0001915 | negative regulation of T cell mediated cytotoxicity | 2/125 | 0.001943456 | 0.010776176 |
| BP | GO:0035747 | natural killer cell chemotaxis | 2/125 | 0.001943456 | 0.010776176 |
| BP | GO:0048865 | stem cell fate commitment | 2/125 | 0.001943456 | 0.010776176 |
| BP | GO:0060054 | positive regulation of epithelial cell proliferation involved in wound healing | 2/125 | 0.001943456 | 0.010776176 |
| BP | GO:0032872 | regulation of stress-activated MAPK cascade | 6/125 | 0.001943664 | 0.010776176 |
| BP | GO:0050688 | regulation of defense response to virus | 4/125 | 0.001974525 | 0.010916267 |
| BP | GO:0048545 | response to steroid hormone | 8/125 | 0.002071424 | 0.011355468 |
| BP | GO:0071214 | cellular response to abiotic stimulus | 8/125 | 0.002071424 | 0.011355468 |
| BP | GO:0104004 | cellular response to environmental stimulus | 8/125 | 0.002071424 | 0.011355468 |
| BP | GO:0070302 | regulation of stress-activated protein kinase signaling cascade | 6/125 | 0.002100341 | 0.011452051 |
| BP | GO:0043368 | positive T cell selection | 3/125 | 0.002100778 | 0.011452051 |
| BP | GO:0001558 | regulation of cell growth | 9/125 | 0.002126136 | 0.011558003 |
| BP | GO:0010565 | regulation of cellular ketone metabolic process | 5/125 | 0.002180048 | 0.011818154 |
| BP | GO:0051279 | regulation of release of sequestered calcium ion into cytosol | 4/125 | 0.002263359 | 0.012142515 |
| BP | GO:0001580 | detection of chemical stimulus involved in sensory perception of bitter taste | 3/125 | 0.002264769 | 0.012142515 |
| BP | GO:0030279 | negative regulation of ossification | 3/125 | 0.002264769 | 0.012142515 |
| BP | GO:0045066 | regulatory T cell differentiation | 3/125 | 0.002264769 | 0.012142515 |
| BP | GO:1904062 | regulation of monoatomic cation transmembrane transport | 8/125 | 0.002350636 | 0.012440203 |
| BP | GO:0002604 | regulation of dendritic cell antigen processing and presentation | 2/125 | 0.002364914 | 0.012440203 |
| BP | GO:0033625 | positive regulation of integrin activation | 2/125 | 0.002364914 | 0.012440203 |
| BP | GO:0033632 | regulation of cell-cell adhesion mediated by integrin | 2/125 | 0.002364914 | 0.012440203 |
| BP | GO:0043320 | natural killer cell degranulation | 2/125 | 0.002364914 | 0.012440203 |
| BP | GO:0045060 | negative thymic T cell selection | 2/125 | 0.002364914 | 0.012440203 |
| BP | GO:0071492 | cellular response to UV-A | 2/125 | 0.002364914 | 0.012440203 |
| BP | GO:0040013 | negative regulation of locomotion | 9/125 | 0.002376128 | 0.012465596 |
| BP | GO:0001953 | negative regulation of cell-matrix adhesion | 3/125 | 0.002436489 | 0.012713905 |
| BP | GO:0050850 | positive regulation of calcium-mediated signaling | 3/125 | 0.002436489 | 0.012713905 |
| BP | GO:0001503 | ossification | 9/125 | 0.002451695 | 0.012759137 |
| BP | GO:0046328 | regulation of JNK cascade | 5/125 | 0.002475187 | 0.012832754 |
| BP | GO:0045765 | regulation of angiogenesis | 8/125 | 0.002478992 | 0.012832754 |
| BP | GO:2001235 | positive regulation of apoptotic signaling pathway | 5/125 | 0.002553293 | 0.013182414 |
| BP | GO:0043281 | regulation of cysteine-type endopeptidase activity involved in apoptotic process | 6/125 | 0.002564904 | 0.013207419 |
| BP | GO:0051281 | positive regulation of release of sequestered calcium ion into cytosol | 3/125 | 0.002616059 | 0.013435385 |
| BP | GO:0022408 | negative regulation of cell-cell adhesion | 6/125 | 0.002627992 | 0.013461243 |
| BP | GO:1901342 | regulation of vasculature development | 8/125 | 0.002752179 | 0.014060457 |
| BP | GO:0043551 | regulation of phosphatidylinositol 3-kinase activity | 3/125 | 0.002803599 | 0.014066558 |
| BP | GO:0033627 | cell adhesion mediated by integrin | 4/125 | 0.002807352 | 0.014066558 |
| BP | GO:0048145 | regulation of fibroblast proliferation | 4/125 | 0.002807352 | 0.014066558 |
| BP | GO:0002645 | positive regulation of tolerance induction | 2/125 | 0.002825451 | 0.014066558 |
| BP | GO:0031953 | negative regulation of protein autophosphorylation | 2/125 | 0.002825451 | 0.014066558 |
| BP | GO:0043383 | negative T cell selection | 2/125 | 0.002825451 | 0.014066558 |
| BP | GO:0071472 | cellular response to salt stress | 2/125 | 0.002825451 | 0.014066558 |
| BP | GO:0071635 | negative regulation of transforming growth factor beta production | 2/125 | 0.002825451 | 0.014066558 |
| BP | GO:0072683 | T cell extravasation | 2/125 | 0.002825451 | 0.014066558 |
| BP | GO:2000508 | regulation of dendritic cell chemotaxis | 2/125 | 0.002825451 | 0.014066558 |
| BP | GO:0033273 | response to vitamin | 4/125 | 0.002925973 | 0.014493065 |
| BP | GO:0050672 | negative regulation of lymphocyte proliferation | 4/125 | 0.002925973 | 0.014493065 |
| BP | GO:0070588 | calcium ion transmembrane transport | 8/125 | 0.002997368 | 0.014743619 |
| BP | GO:0032689 | negative regulation of type II interferon production | 3/125 | 0.002999221 | 0.014743619 |
| BP | GO:0097178 | ruffle assembly | 3/125 | 0.002999221 | 0.014743619 |
| BP | GO:0032945 | negative regulation of mononuclear cell proliferation | 4/125 | 0.003047979 | 0.014945655 |
| BP | GO:0031348 | negative regulation of defense response | 7/125 | 0.00308671 | 0.01509764 |
| BP | GO:0120161 | regulation of cold-induced thermogenesis | 5/125 | 0.003151038 | 0.015373747 |
| BP | GO:0043029 | T cell homeostasis | 3/125 | 0.003203039 | 0.015549708 |
| BP | GO:0050913 | sensory perception of bitter taste | 3/125 | 0.003203039 | 0.015549708 |
| BP | GO:0106106 | cold-induced thermogenesis | 5/125 | 0.003243995 | 0.015709462 |
| BP | GO:2000117 | negative regulation of cysteine-type endopeptidase activity | 4/125 | 0.00330233 | 0.01582461 |
| BP | GO:0002664 | regulation of T cell tolerance induction | 2/125 | 0.003324534 | 0.01582461 |
| BP | GO:0010642 | negative regulation of platelet-derived growth factor receptor signaling pathway | 2/125 | 0.003324534 | 0.01582461 |
| BP | GO:0030157 | pancreatic juice secretion | 2/125 | 0.003324534 | 0.01582461 |
| BP | GO:0046541 | saliva secretion | 2/125 | 0.003324534 | 0.01582461 |
| BP | GO:0070486 | leukocyte aggregation | 2/125 | 0.003324534 | 0.01582461 |
| BP | GO:0071801 | regulation of podosome assembly | 2/125 | 0.003324534 | 0.01582461 |
| BP | GO:0042180 | cellular ketone metabolic process | 6/125 | 0.003401419 | 0.016137898 |
| BP | GO:0050912 | detection of chemical stimulus involved in sensory perception of taste | 3/125 | 0.003415159 | 0.016137898 |
| BP | GO:0097028 | dendritic cell differentiation | 3/125 | 0.003415159 | 0.016137898 |
| BP | GO:0001656 | metanephros development | 4/125 | 0.003434768 | 0.016157278 |
| BP | GO:1903038 | negative regulation of leukocyte cell-cell adhesion | 5/125 | 0.003435818 | 0.016157278 |
| BP | GO:1904427 | positive regulation of calcium ion transmembrane transport | 4/125 | 0.003570777 | 0.016751569 |
| BP | GO:0072006 | nephron development | 5/125 | 0.003635672 | 0.017015112 |
| BP | GO:0001666 | response to hypoxia | 7/125 | 0.003663891 | 0.017106154 |
| BP | GO:0030336 | negative regulation of cell migration | 8/125 | 0.003836396 | 0.017746619 |
| BP | GO:0001910 | regulation of leukocyte mediated cytotoxicity | 4/125 | 0.003853685 | 0.017746619 |
| BP | GO:0014033 | neural crest cell differentiation | 4/125 | 0.003853685 | 0.017746619 |
| BP | GO:0019217 | regulation of fatty acid metabolic process | 4/125 | 0.003853685 | 0.017746619 |
| BP | GO:0046330 | positive regulation of JNK cascade | 4/125 | 0.003853685 | 0.017746619 |
| BP | GO:0070141 | response to UV-A | 2/125 | 0.003861631 | 0.017746619 |
| BP | GO:0030521 | androgen receptor signaling pathway | 3/125 | 0.003864723 | 0.017746619 |
| BP | GO:0007044 | cell-substrate junction assembly | 4/125 | 0.004000675 | 0.018242134 |
| BP | GO:0070664 | negative regulation of leukocyte proliferation | 4/125 | 0.004000675 | 0.018242134 |
| BP | GO:0072080 | nephron tubule development | 4/125 | 0.004000675 | 0.018242134 |
| BP | GO:0035850 | epithelial cell differentiation involved in kidney development | 3/125 | 0.004102368 | 0.018575626 |
| BP | GO:0035987 | endodermal cell differentiation | 3/125 | 0.004102368 | 0.018575626 |
| BP | GO:0043300 | regulation of leukocyte degranulation | 3/125 | 0.004102368 | 0.018575626 |
| BP | GO:0002709 | regulation of T cell mediated immunity | 4/125 | 0.004151415 | 0.018754202 |
| BP | GO:0046777 | protein autophosphorylation | 6/125 | 0.004333092 | 0.019368305 |
| BP | GO:0030838 | positive regulation of actin filament polymerization | 3/125 | 0.004348715 | 0.019368305 |
| BP | GO:0071470 | cellular response to osmotic stress | 3/125 | 0.004348715 | 0.019368305 |
| BP | GO:0001768 | establishment of T cell polarity | 2/125 | 0.004436219 | 0.019368305 |
| BP | GO:0001771 | immunological synapse formation | 2/125 | 0.004436219 | 0.019368305 |
| BP | GO:0002517 | T cell tolerance induction | 2/125 | 0.004436219 | 0.019368305 |
| BP | GO:0002679 | respiratory burst involved in defense response | 2/125 | 0.004436219 | 0.019368305 |
| BP | GO:0002864 | regulation of acute inflammatory response to antigenic stimulus | 2/125 | 0.004436219 | 0.019368305 |
| BP | GO:0010820 | positive regulation of T cell chemotaxis | 2/125 | 0.004436219 | 0.019368305 |
| BP | GO:0014029 | neural crest formation | 2/125 | 0.004436219 | 0.019368305 |
| BP | GO:0046348 | amino sugar catabolic process | 2/125 | 0.004436219 | 0.019368305 |
| BP | GO:0071474 | cellular hyperosmotic response | 2/125 | 0.004436219 | 0.019368305 |
| BP | GO:0098760 | response to interleukin-7 | 2/125 | 0.004436219 | 0.019368305 |
| BP | GO:0098761 | cellular response to interleukin-7 | 2/125 | 0.004436219 | 0.019368305 |
| BP | GO:2000402 | negative regulation of lymphocyte migration | 2/125 | 0.004436219 | 0.019368305 |
| BP | GO:0001914 | regulation of T cell mediated cytotoxicity | 3/125 | 0.004603858 | 0.020031231 |
| BP | GO:0061326 | renal tubule development | 4/125 | 0.004626578 | 0.020031231 |
| BP | GO:0036293 | response to decreased oxygen levels | 7/125 | 0.00463849 | 0.020031231 |
| BP | GO:0051017 | actin filament bundle assembly | 5/125 | 0.00463938 | 0.020031231 |
| BP | GO:1990845 | adaptive thermogenesis | 5/125 | 0.00463938 | 0.020031231 |
| BP | GO:2000146 | negative regulation of cell motility | 8/125 | 0.004846953 | 0.020881263 |
| BP | GO:0001822 | kidney development | 7/125 | 0.004974529 | 0.021322807 |
| BP | GO:0034765 | regulation of monoatomic ion transmembrane transport | 9/125 | 0.004983158 | 0.021322807 |
| BP | GO:0060560 | developmental growth involved in morphogenesis | 6/125 | 0.005016719 | 0.021322807 |
| BP | GO:0001767 | establishment of lymphocyte polarity | 2/125 | 0.005047778 | 0.021322807 |
| BP | GO:0030852 | regulation of granulocyte differentiation | 2/125 | 0.005047778 | 0.021322807 |
| BP | GO:0034638 | phosphatidylcholine catabolic process | 2/125 | 0.005047778 | 0.021322807 |
| BP | GO:0050862 | positive regulation of T cell receptor signaling pathway | 2/125 | 0.005047778 | 0.021322807 |
| BP | GO:1902931 | negative regulation of alcohol biosynthetic process | 2/125 | 0.005047778 | 0.021322807 |
| BP | GO:2000696 | regulation of epithelial cell differentiation involved in kidney development | 2/125 | 0.005047778 | 0.021322807 |
| BP | GO:0001776 | leukocyte homeostasis | 4/125 | 0.005137063 | 0.021401988 |
| BP | GO:0048144 | fibroblast proliferation | 4/125 | 0.005137063 | 0.021401988 |
| BP | GO:0150115 | cell-substrate junction organization | 4/125 | 0.005137063 | 0.021401988 |
| BP | GO:0002762 | negative regulation of myeloid leukocyte differentiation | 3/125 | 0.005140882 | 0.021401988 |
| BP | GO:0051496 | positive regulation of stress fiber assembly | 3/125 | 0.005140882 | 0.021401988 |
| BP | GO:0001959 | regulation of cytokine-mediated signaling pathway | 5/125 | 0.005143288 | 0.021401988 |
| BP | GO:0061572 | actin filament bundle organization | 5/125 | 0.005143288 | 0.021401988 |
| BP | GO:0010466 | negative regulation of peptidase activity | 6/125 | 0.005225833 | 0.021699204 |
| BP | GO:0030856 | regulation of epithelial cell differentiation | 5/125 | 0.005275091 | 0.021857233 |
| BP | GO:1902904 | negative regulation of supramolecular fiber organization | 5/125 | 0.00540927 | 0.0223276 |
| BP | GO:0050873 | brown fat cell differentiation | 3/125 | 0.005422933 | 0.0223276 |
| BP | GO:0071622 | regulation of granulocyte chemotaxis | 3/125 | 0.005422933 | 0.0223276 |
| BP | GO:0032760 | positive regulation of tumor necrosis factor production | 4/125 | 0.005497576 | 0.022587271 |
| BP | GO:0051047 | positive regulation of secretion | 7/125 | 0.005606375 | 0.022985891 |
| BP | GO:0033623 | regulation of integrin activation | 2/125 | 0.005695793 | 0.023136003 |
| BP | GO:0033631 | cell-cell adhesion mediated by integrin | 2/125 | 0.005695793 | 0.023136003 |
| BP | GO:0072224 | metanephric glomerulus development | 2/125 | 0.005695793 | 0.023136003 |
| BP | GO:0009409 | response to cold | 3/125 | 0.005714119 | 0.023136003 |
| BP | GO:0043303 | mast cell degranulation | 3/125 | 0.005714119 | 0.023136003 |
| BP | GO:0043550 | regulation of lipid kinase activity | 3/125 | 0.005714119 | 0.023136003 |
| BP | GO:0007254 | JNK cascade | 5/125 | 0.005826288 | 0.023541328 |
| BP | GO:0072001 | renal system development | 7/125 | 0.00589487 | 0.023769225 |
| BP | GO:0002686 | negative regulation of leukocyte migration | 3/125 | 0.006014516 | 0.024201654 |
| BP | GO:0032231 | regulation of actin filament bundle assembly | 4/125 | 0.00606946 | 0.02437249 |
| BP | GO:0007259 | receptor signaling pathway via JAK-STAT | 5/125 | 0.006265498 | 0.024851487 |
| BP | GO:0033209 | tumor necrosis factor-mediated signaling pathway | 4/125 | 0.006268547 | 0.024851487 |
| BP | GO:0045833 | negative regulation of lipid metabolic process | 4/125 | 0.006268547 | 0.024851487 |
| BP | GO:1903557 | positive regulation of tumor necrosis factor superfamily cytokine production | 4/125 | 0.006268547 | 0.024851487 |
| BP | GO:0001667 | ameboidal-type cell migration | 9/125 | 0.006305666 | 0.024851487 |
| BP | GO:0002279 | mast cell activation involved in immune response | 3/125 | 0.006324199 | 0.024851487 |
| BP | GO:0045444 | fat cell differentiation | 6/125 | 0.006368615 | 0.024851487 |
| BP | GO:0003159 | morphogenesis of an endothelium | 2/125 | 0.006379755 | 0.024851487 |
| BP | GO:0032769 | negative regulation of monooxygenase activity | 2/125 | 0.006379755 | 0.024851487 |
| BP | GO:0050965 | detection of temperature stimulus involved in sensory perception of pain | 2/125 | 0.006379755 | 0.024851487 |
| BP | GO:0051770 | positive regulation of nitric-oxide synthase biosynthetic process | 2/125 | 0.006379755 | 0.024851487 |
| BP | GO:0060263 | regulation of respiratory burst | 2/125 | 0.006379755 | 0.024851487 |
| BP | GO:0061154 | endothelial tube morphogenesis | 2/125 | 0.006379755 | 0.024851487 |
| BP | GO:0072077 | renal vesicle morphogenesis | 2/125 | 0.006379755 | 0.024851487 |
| BP | GO:0090594 | inflammatory response to wounding | 2/125 | 0.006379755 | 0.024851487 |
| BP | GO:0002444 | myeloid leukocyte mediated immunity | 4/125 | 0.00647193 | 0.025110303 |
| BP | GO:0031341 | regulation of cell killing | 4/125 | 0.00647193 | 0.025110303 |
| BP | GO:0001706 | endoderm formation | 3/125 | 0.006643242 | 0.025622155 |
| BP | GO:0002448 | mast cell mediated immunity | 3/125 | 0.006643242 | 0.025622155 |
| BP | GO:0007566 | embryo implantation | 3/125 | 0.006643242 | 0.025622155 |
| BP | GO:0051341 | regulation of oxidoreductase activity | 4/125 | 0.006679648 | 0.025711757 |
| BP | GO:0002440 | production of molecular mediator of immune response | 7/125 | 0.006718579 | 0.025759995 |
| BP | GO:0048638 | regulation of developmental growth | 7/125 | 0.006718579 | 0.025759995 |
| BP | GO:0060759 | regulation of response to cytokine stimulus | 5/125 | 0.006886669 | 0.026269092 |
| BP | GO:0032611 | interleukin-1 beta production | 4/125 | 0.00689174 | 0.026269092 |
| BP | GO:0032651 | regulation of interleukin-1 beta production | 4/125 | 0.00689174 | 0.026269092 |
| BP | GO:0030225 | macrophage differentiation | 3/125 | 0.006971712 | 0.026470517 |
| BP | GO:0061005 | cell differentiation involved in kidney development | 3/125 | 0.006971712 | 0.026470517 |
| BP | GO:0062012 | regulation of small molecule metabolic process | 7/125 | 0.007048585 | 0.026643405 |
| BP | GO:0002283 | neutrophil activation involved in immune response | 2/125 | 0.00709916 | 0.026643405 |
| BP | GO:0002523 | leukocyte migration involved in inflammatory response | 2/125 | 0.00709916 | 0.026643405 |
| BP | GO:0032695 | negative regulation of interleukin-12 production | 2/125 | 0.00709916 | 0.026643405 |
| BP | GO:0071800 | podosome assembly | 2/125 | 0.00709916 | 0.026643405 |
| BP | GO:0072087 | renal vesicle development | 2/125 | 0.00709916 | 0.026643405 |
| BP | GO:0001659 | temperature homeostasis | 5/125 | 0.007212888 | 0.026966513 |
| BP | GO:0007204 | positive regulation of cytosolic calcium ion concentration | 5/125 | 0.007212888 | 0.026966513 |
| BP | GO:0070482 | response to oxygen levels | 7/125 | 0.007275189 | 0.027120551 |
| BP | GO:0001913 | T cell mediated cytotoxicity | 3/125 | 0.007309676 | 0.027120551 |
| BP | GO:0001961 | positive regulation of cytokine-mediated signaling pathway | 3/125 | 0.007309676 | 0.027120551 |
| BP | GO:0070228 | regulation of lymphocyte apoptotic process | 3/125 | 0.007309676 | 0.027120551 |
| BP | GO:0050678 | regulation of epithelial cell proliferation | 8/125 | 0.007368366 | 0.027286429 |
| BP | GO:0048762 | mesenchymal cell differentiation | 6/125 | 0.007543902 | 0.027852468 |
| BP | GO:0048771 | tissue remodeling | 5/125 | 0.007549761 | 0.027852468 |
| BP | GO:0002886 | regulation of myeloid leukocyte mediated immunity | 3/125 | 0.007657199 | 0.028142425 |
| BP | GO:0043407 | negative regulation of MAP kinase activity | 3/125 | 0.007657199 | 0.028142425 |
| BP | GO:0034440 | lipid oxidation | 4/125 | 0.007784606 | 0.028556906 |
| BP | GO:0002577 | regulation of antigen processing and presentation | 2/125 | 0.007853509 | 0.028648116 |
| BP | GO:0002643 | regulation of tolerance induction | 2/125 | 0.007853509 | 0.028648116 |
| BP | GO:2000251 | positive regulation of actin cytoskeleton reorganization | 2/125 | 0.007853509 | 0.028648116 |
| BP | GO:0009266 | response to temperature stimulus | 5/125 | 0.007897461 | 0.028701153 |
| BP | GO:0043409 | negative regulation of MAPK cascade | 5/125 | 0.007897461 | 0.028701153 |
| BP | GO:0045927 | positive regulation of growth | 6/125 | 0.007968378 | 0.028905054 |
| BP | GO:0009308 | amine metabolic process | 4/125 | 0.008019134 | 0.02898143 |
| BP | GO:0090630 | activation of GTPase activity | 4/125 | 0.008019134 | 0.02898143 |
| BP | GO:0002706 | regulation of lymphocyte mediated immunity | 5/125 | 0.008075424 | 0.029077173 |
| BP | GO:0097696 | receptor signaling pathway via STAT | 5/125 | 0.008075424 | 0.029077173 |
| BP | GO:0050830 | defense response to Gram-positive bacterium | 4/125 | 0.008258258 | 0.029680742 |
| BP | GO:0022617 | extracellular matrix disassembly | 3/125 | 0.008381162 | 0.030011924 |
| BP | GO:0032387 | negative regulation of intracellular transport | 3/125 | 0.008381162 | 0.030011924 |
| BP | GO:0051896 | regulation of protein kinase B signaling | 5/125 | 0.008439682 | 0.030110979 |
| BP | GO:1903169 | regulation of calcium ion transmembrane transport | 5/125 | 0.008439682 | 0.030110979 |
| BP | GO:0007565 | female pregnancy | 5/125 | 0.008626018 | 0.030334793 |
| BP | GO:0050728 | negative regulation of inflammatory response | 5/125 | 0.008626018 | 0.030334793 |
| BP | GO:0030502 | negative regulation of bone mineralization | 2/125 | 0.008642307 | 0.030334793 |
| BP | GO:0038083 | peptidyl-tyrosine autophosphorylation | 2/125 | 0.008642307 | 0.030334793 |
| BP | GO:0050849 | negative regulation of calcium-mediated signaling | 2/125 | 0.008642307 | 0.030334793 |
| BP | GO:0071605 | monocyte chemotactic protein-1 production | 2/125 | 0.008642307 | 0.030334793 |
| BP | GO:0071637 | regulation of monocyte chemotactic protein-1 production | 2/125 | 0.008642307 | 0.030334793 |
| BP | GO:0090026 | positive regulation of monocyte chemotaxis | 2/125 | 0.008642307 | 0.030334793 |
| BP | GO:1901739 | regulation of myoblast fusion | 2/125 | 0.008642307 | 0.030334793 |
| BP | GO:0032731 | positive regulation of interleukin-1 beta production | 3/125 | 0.008757717 | 0.030574913 |
| BP | GO:0032757 | positive regulation of interleukin-8 production | 3/125 | 0.008757717 | 0.030574913 |
| BP | GO:2001244 | positive regulation of intrinsic apoptotic signaling pathway | 3/125 | 0.008757717 | 0.030574913 |
| BP | GO:0071466 | cellular response to xenobiotic stimulus | 5/125 | 0.009007211 | 0.031389792 |
| BP | GO:0051893 | regulation of focal adhesion assembly | 3/125 | 0.009144061 | 0.031753304 |
| BP | GO:0090109 | regulation of cell-substrate junction assembly | 3/125 | 0.009144061 | 0.031753304 |
| BP | GO:0002822 | regulation of adaptive immune response based on somatic recombination of immune receptors built from immunoglobulin superfamily domains | 5/125 | 0.009202109 | 0.03189812 |
| BP | GO:0071478 | cellular response to radiation | 5/125 | 0.009399901 | 0.032406711 |
| BP | GO:0032928 | regulation of superoxide anion generation | 2/125 | 0.009465067 | 0.032406711 |
| BP | GO:0033630 | positive regulation of cell adhesion mediated by integrin | 2/125 | 0.009465067 | 0.032406711 |
| BP | GO:0050961 | detection of temperature stimulus involved in sensory perception | 2/125 | 0.009465067 | 0.032406711 |
| BP | GO:0051767 | nitric-oxide synthase biosynthetic process | 2/125 | 0.009465067 | 0.032406711 |
| BP | GO:0051769 | regulation of nitric-oxide synthase biosynthetic process | 2/125 | 0.009465067 | 0.032406711 |
| BP | GO:2000479 | regulation of cAMP-dependent protein kinase activity | 2/125 | 0.009465067 | 0.032406711 |
| BP | GO:0002761 | regulation of myeloid leukocyte differentiation | 4/125 | 0.009524071 | 0.032436482 |
| BP | GO:0071901 | negative regulation of protein serine/threonine kinase activity | 4/125 | 0.009524071 | 0.032436482 |
| BP | GO:0031663 | lipopolysaccharide-mediated signaling pathway | 3/125 | 0.009540245 | 0.032436482 |
| BP | GO:1905330 | regulation of morphogenesis of an epithelium | 3/125 | 0.009540245 | 0.032436482 |
| BP | GO:0009410 | response to xenobiotic stimulus | 8/125 | 0.00973756 | 0.033049769 |
| BP | GO:0001655 | urogenital system development | 3/125 | 0.009946317 | 0.033641288 |
| BP | GO:0060760 | positive regulation of response to cytokine stimulus | 3/125 | 0.009946317 | 0.033641288 |
| BP | GO:0002456 | T cell mediated immunity | 4/125 | 0.01006379 | 0.033862553 |
| BP | GO:0002832 | negative regulation of response to biotic stimulus | 4/125 | 0.01006379 | 0.033862553 |
| BP | GO:0031623 | receptor internalization | 4/125 | 0.01006379 | 0.033862553 |
| BP | GO:0120032 | regulation of plasma membrane bounded cell projection assembly | 5/125 | 0.01022042 | 0.034314886 |
| BP | GO:0002689 | negative regulation of leukocyte chemotaxis | 2/125 | 0.010321303 | 0.034314886 |
| BP | GO:0010560 | positive regulation of glycoprotein biosynthetic process | 2/125 | 0.010321303 | 0.034314886 |
| BP | GO:0032727 | positive regulation of interferon-alpha production | 2/125 | 0.010321303 | 0.034314886 |
| BP | GO:0045591 | positive regulation of regulatory T cell differentiation | 2/125 | 0.010321303 | 0.034314886 |
| BP | GO:0071305 | cellular response to vitamin D | 2/125 | 0.010321303 | 0.034314886 |
| BP | GO:1900120 | regulation of receptor binding | 2/125 | 0.010321303 | 0.034314886 |
| BP | GO:0019730 | antimicrobial humoral response | 4/125 | 0.010340938 | 0.034321695 |
| BP | GO:0050909 | sensory perception of taste | 3/125 | 0.010362325 | 0.034334287 |
| BP | GO:0007369 | gastrulation | 5/125 | 0.010432987 | 0.034509827 |
| CC | GO:0009897 | external side of plasma membrane | 19/129 | 5.27E-11 | 7.43E-09 |
| CC | GO:0030666 | endocytic vesicle membrane | 11/129 | 7.50E-08 | 5.29E-06 |
| CC | GO:0001772 | immunological synapse | 6/129 | 3.70E-07 | 1.74E-05 |
| CC | GO:0042105 | alpha-beta T cell receptor complex | 4/129 | 8.65E-07 | 3.05E-05 |
| CC | GO:0030139 | endocytic vesicle | 12/129 | 3.35E-06 | 9.45E-05 |
| CC | GO:0042613 | MHC class II protein complex | 4/129 | 4.05E-06 | 9.53E-05 |
| CC | GO:0030669 | clathrin-coated endocytic vesicle membrane | 6/129 | 8.79E-06 | 0.00017704 |
| CC | GO:0042611 | MHC protein complex | 4/129 | 2.07E-05 | 0.000364731 |
| CC | GO:0045334 | clathrin-coated endocytic vesicle | 6/129 | 3.32E-05 | 0.000520673 |

**Table S5b. Analysis of KEGG**.

| ID | Description | GeneRatio | pvalue | qvalue |
| --- | --- | --- | --- | --- |
| hsa04659 | Th17 cell differentiation | 12/86 | 6.01E-10 | 7.78E-08 |
| hsa04658 | Th1 and Th2 cell differentiation | 11/86 | 1.49E-09 | 9.62E-08 |
| hsa04061 | Viral protein interaction with cytokine and cytokine receptor | 11/86 | 3.67E-09 | 1.58E-07 |
| hsa04062 | Chemokine signaling pathway | 13/86 | 4.91E-08 | 1.59E-06 |
| hsa05340 | Primary immunodeficiency | 7/86 | 7.81E-08 | 2.02E-06 |
| hsa05416 | Viral myocarditis | 8/86 | 1.23E-07 | 2.65E-06 |
| hsa04640 | Hematopoietic cell lineage | 9/86 | 5.56E-07 | 1.03E-05 |
| hsa04060 | Cytokine-cytokine receptor interaction | 14/86 | 1.26E-06 | 2.04E-05 |
| hsa05166 | Human T-cell leukemia virus 1 infection | 12/86 | 1.90E-06 | 2.73E-05 |
| hsa05321 | Inflammatory bowel disease | 7/86 | 3.46E-06 | 4.48E-05 |
| hsa05150 | Staphylococcus aureus infection | 8/86 | 4.75E-06 | 5.31E-05 |
| hsa05169 | Epstein-Barr virus infection | 11/86 | 4.92E-06 | 5.31E-05 |
| hsa05152 | Tuberculosis | 10/86 | 1.15E-05 | 0.000114534 |
| hsa05310 | Asthma | 5/86 | 1.25E-05 | 0.000115364 |
| hsa04514 | Cell adhesion molecules | 9/86 | 2.66E-05 | 0.000229865 |
| hsa05330 | Allograft rejection | 5/86 | 3.49E-05 | 0.000282209 |
| hsa05323 | Rheumatoid arthritis | 7/86 | 3.76E-05 | 0.000286131 |
| hsa05332 | Graft-versus-host disease | 5/86 | 5.73E-05 | 0.000411961 |
| hsa04940 | Type I diabetes mellitus | 5/86 | 6.43E-05 | 0.000438171 |
| hsa05140 | Leishmaniasis | 6/86 | 0.00011434 | 0.000723187 |
| hsa04672 | Intestinal immune network for IgA production | 5/86 | 0.000121509 | 0.000723187 |
| hsa04612 | Antigen processing and presentation | 6/86 | 0.000122883 | 0.000723187 |
| hsa05320 | Autoimmune thyroid disease | 5/86 | 0.000177203 | 0.000997528 |
| hsa05235 | PD-L1 expression and PD-1 checkpoint pathway in cancer | 6/86 | 0.00025464 | 0.001373714 |
| hsa05164 | Influenza A | 8/86 | 0.000299582 | 0.001551522 |
| hsa04650 | Natural killer cell mediated cytotoxicity | 7/86 | 0.00034122 | 0.001699195 |
| hsa04145 | Phagosome | 7/86 | 0.000796822 | 0.003821017 |
| hsa05145 | Toxoplasmosis | 6/86 | 0.0008326 | 0.003849993 |
| hsa04970 | Salivary secretion | 5/86 | 0.002346386 | 0.010475696 |
| hsa05162 | Measles | 6/86 | 0.002549089 | 0.011001334 |
| hsa05142 | Chagas disease | 5/86 | 0.0035056 | 0.014641385 |
| hsa04620 | Toll-like receptor signaling pathway | 5/86 | 0.003810711 | 0.015418336 |
| hsa04670 | Leukocyte transendothelial migration | 5/86 | 0.00583806 | 0.022905307 |
| hsa04630 | JAK-STAT signaling pathway | 6/86 | 0.006303056 | 0.024002349 |
| hsa04660 | T cell receptor signaling pathway | 5/86 | 0.007217496 | 0.026699308 |

# Appendix 6

**GSEA analysis**

**Table 6a. GO of GSEA analysis**.

| ID | enrichmentScore | NES | pvalue | qvalue |
| --- | --- | --- | --- | --- |
| GOBP_ADAPTIVE_IMMUNE_RESPONSE | -0.763122496 | -3.268578421 | 1.00E-10 | 2.75E-09 |
| GOBP_ADAPTIVE_IMMUNE_RESPONSE_BASED_ON_SOMATIC_RECOMBINATION_OF_IMMUNE_RECEPTORS_BUILT_FROM_IMMUNOGLOBULIN_SUPERFAMILY_DOMAINS | -0.754191328 | -3.08742522 | 1.00E-10 | 2.75E-09 |
| GOBP_LYMPHOCYTE_MEDIATED_IMMUNITY | -0.750478641 | -3.068136395 | 1.00E-10 | 2.75E-09 |
| GOBP_LEUKOCYTE_MEDIATED_IMMUNITY | -0.727518388 | -3.059041384 | 1.00E-10 | 2.75E-09 |
| GOBP_IMMUNE_RESPONSE_REGULATING_CELL_SURFACE_RECEPTOR_SIGNALING_PATHWAY | -0.74118031 | -3.050859668 | 1.00E-10 | 2.75E-09 |
| GOBP_ANTIGEN_RECEPTOR_MEDIATED_SIGNALING_PATHWAY | -0.784118562 | -3.024284664 | 1.00E-10 | 2.75E-09 |
| GOBP_IMMUNE_RESPONSE_REGULATING_SIGNALING_PATHWAY | -0.710682151 | -3.020890074 | 1.00E-10 | 2.75E-09 |
| GOBP_POSITIVE_REGULATION_OF_CELL_ACTIVATION | -0.700461088 | -2.948102642 | 1.00E-10 | 2.75E-09 |
| GOBP_ACTIVATION_OF_IMMUNE_RESPONSE | -0.688035176 | -2.946615988 | 1.00E-10 | 2.75E-09 |
| GOBP_REGULATION_OF_LYMPHOCYTE_ACTIVATION | -0.683580403 | -2.942820482 | 1.00E-10 | 2.75E-09 |
| GOBP_POSITIVE_REGULATION_OF_LEUKOCYTE_CELL_CELL_ADHESION | -0.725157215 | -2.934511296 | 1.00E-10 | 2.75E-09 |
| GOBP_POSITIVE_REGULATION_OF_LYMPHOCYTE_ACTIVATION | -0.709093404 | -2.922725719 | 1.00E-10 | 2.75E-09 |
| GOBP_REGULATION_OF_ADAPTIVE_IMMUNE_RESPONSE | -0.751854162 | -2.918377724 | 1.00E-10 | 2.75E-09 |
| GOBP_ANTIGEN_PROCESSING_AND_PRESENTATION | -0.815822969 | -2.90751676 | 1.00E-10 | 2.75E-09 |
| GOBP_LEUKOCYTE_MEDIATED_CYTOTOXICITY | -0.784596999 | -2.904882501 | 1.00E-10 | 2.75E-09 |
| GOBP_REGULATION_OF_T_CELL_ACTIVATION | -0.690859019 | -2.899306029 | 1.00E-10 | 2.75E-09 |
| GOBP_ALPHA_BETA_T_CELL_ACTIVATION | -0.759671508 | -2.897379065 | 1.00E-10 | 2.75E-09 |
| GOBP_LEUKOCYTE_CELL_CELL_ADHESION | -0.682840391 | -2.8797723 | 1.00E-10 | 2.75E-09 |
| GOBP_CELL_ACTIVATION_INVOLVED_IN_IMMUNE_RESPONSE | -0.696314959 | -2.856097319 | 1.00E-10 | 2.75E-09 |
| GOBP_REGULATION_OF_LEUKOCYTE_MEDIATED_IMMUNITY | -0.716745065 | -2.854408888 | 1.00E-10 | 2.75E-09 |
| GOBP_T_CELL_RECEPTOR_SIGNALING_PATHWAY | -0.762737768 | -2.833712498 | 1.00E-10 | 2.75E-09 |
| GOBP_POSITIVE_REGULATION_OF_ADAPTIVE_IMMUNE_RESPONSE | -0.78143405 | -2.831440398 | 1.00E-10 | 2.75E-09 |
| GOBP_LEUKOCYTE_PROLIFERATION | -0.680361517 | -2.830153503 | 1.00E-10 | 2.75E-09 |
| GOBP_TYPE_II_INTERFERON_PRODUCTION | -0.783805326 | -2.826330323 | 1.00E-10 | 2.75E-09 |
| GOBP_POSITIVE_REGULATION_OF_CELL_CELL_ADHESION | -0.685478164 | -2.824439105 | 1.00E-10 | 2.75E-09 |
| GOBP_B_CELL_RECEPTOR_SIGNALING_PATHWAY | -0.821052995 | -2.818345911 | 1.00E-10 | 2.75E-09 |
| GOBP_POSITIVE_REGULATION_OF_TYPE_II_INTERFERON_PRODUCTION | -0.802772825 | -2.808167268 | 1.00E-10 | 2.75E-09 |
| GOBP_REGULATION_OF_IMMUNE_EFFECTOR_PROCESS | -0.667250627 | -2.792959719 | 1.00E-10 | 2.75E-09 |
| GOBP_T_CELL_DIFFERENTIATION | -0.680015901 | -2.792383184 | 1.00E-10 | 2.75E-09 |
| GOBP_ANTIGEN_PROCESSING_AND_PRESENTATION_OF_PEPTIDE_ANTIGEN | -0.819105134 | -2.783453655 | 1.00E-10 | 2.75E-09 |
| GOBP_REGULATION_OF_LYMPHOCYTE_MEDIATED_IMMUNITY | -0.726605983 | -2.778388707 | 1.00E-10 | 2.75E-09 |
| GOBP_B_CELL_ACTIVATION | -0.687278948 | -2.778361455 | 1.00E-10 | 2.75E-09 |
| GOBP_LYMPHOCYTE_ACTIVATION_INVOLVED_IN_IMMUNE_RESPONSE | -0.710268109 | -2.773882159 | 1.00E-10 | 2.75E-09 |
| GOBP_REGULATION_OF_INNATE_IMMUNE_RESPONSE | -0.659942474 | -2.765651863 | 1.00E-10 | 2.75E-09 |
| GOBP_POSITIVE_REGULATION_OF_ALPHA_BETA_T_CELL_ACTIVATION | -0.795619048 | -2.76435848 | 1.00E-10 | 2.75E-09 |
| GOBP_MONONUCLEAR_CELL_DIFFERENTIATION | -0.643403762 | -2.759290178 | 1.00E-10 | 2.75E-09 |
| GOBP_NATURAL_KILLER_CELL_MEDIATED_IMMUNITY | -0.787251581 | -2.752694533 | 1.00E-10 | 2.75E-09 |
| GOBP_T_CELL_MEDIATED_IMMUNITY | -0.751703905 | -2.750529596 | 1.00E-10 | 2.75E-09 |
| GOBP_REGULATION_OF_LEUKOCYTE_PROLIFERATION | -0.682669934 | -2.749923518 | 1.00E-10 | 2.75E-09 |
| GOBP_REGULATION_OF_T_CELL_MEDIATED_IMMUNITY | -0.771225549 | -2.743653265 | 1.00E-10 | 2.75E-09 |
| GOBP_REGULATION_OF_ANTIGEN_RECEPTOR_MEDIATED_SIGNALING_PATHWAY | -0.806971992 | -2.742223248 | 1.00E-10 | 2.75E-09 |
| GOBP_REGULATION_OF_B_CELL_ACTIVATION | -0.748439924 | -2.741989111 | 1.00E-10 | 2.75E-09 |
| GOBP_POSITIVE_REGULATION_OF_LEUKOCYTE_MEDIATED_IMMUNITY | -0.737971116 | -2.741699785 | 1.00E-10 | 2.75E-09 |
| GOBP_ALPHA_BETA_T_CELL_DIFFERENTIATION | -0.754459568 | -2.741477924 | 1.00E-10 | 2.75E-09 |
| GOBP_POSITIVE_REGULATION_OF_CYTOKINE_PRODUCTION | -0.637792084 | -2.736888804 | 1.00E-10 | 2.75E-09 |
| GOBP_REGULATION_OF_ALPHA_BETA_T_CELL_DIFFERENTIATION | -0.784152196 | -2.73462916 | 1.00E-10 | 2.75E-09 |
| GOBP_B_CELL_MEDIATED_IMMUNITY | -0.737012567 | -2.73361405 | 1.00E-10 | 2.75E-09 |
| GOBP_POSITIVE_REGULATION_OF_IMMUNE_EFFECTOR_PROCESS | -0.681871591 | -2.733322176 | 1.00E-10 | 2.75E-09 |
| GOBP_T_CELL_PROLIFERATION | -0.696375689 | -2.730535612 | 1.00E-10 | 2.75E-09 |
| GOBP_REGULATION_OF_LEUKOCYTE_MEDIATED_CYTOTOXICITY | -0.772495463 | -2.725669835 | 1.00E-10 | 2.75E-09 |
| GOBP_CD4_POSITIVE_ALPHA_BETA_T_CELL_DIFFERENTIATION | -0.772803754 | -2.724579166 | 1.00E-10 | 2.75E-09 |
| GOBP_POSITIVE_REGULATION_OF_LYMPHOCYTE_MEDIATED_IMMUNITY | -0.752627014 | -2.719177804 | 1.00E-10 | 2.75E-09 |
| GOBP_T_CELL_ACTIVATION_INVOLVED_IN_IMMUNE_RESPONSE | -0.747330129 | -2.715571697 | 1.00E-10 | 2.75E-09 |
| GOBP_CELLULAR_DEFENSE_RESPONSE | -0.831943074 | -2.715428802 | 1.00E-10 | 2.75E-09 |
| GOBP_B_CELL_PROLIFERATION | -0.757762145 | -2.702908788 | 1.00E-10 | 2.75E-09 |
| GOBP_REGULATION_OF_ALPHA_BETA_T_CELL_ACTIVATION | -0.748864753 | -2.697647163 | 1.00E-10 | 2.75E-09 |
| GOBP_T_CELL_SELECTION | -0.816173197 | -2.694275167 | 1.00E-10 | 2.75E-09 |
| GOBP_REGULATION_OF_RESPONSE_TO_BIOTIC_STIMULUS | -0.630817895 | -2.694174052 | 1.00E-10 | 2.75E-09 |
| GOBP_MYELOID_LEUKOCYTE_ACTIVATION | -0.679502885 | -2.693143123 | 1.00E-10 | 2.75E-09 |
| GOBP_RESPONSE_TO_TYPE_II_INTERFERON | -0.730457808 | -2.693071888 | 1.00E-10 | 2.75E-09 |
| GOBP_ANTIGEN_PROCESSING_AND_PRESENTATION_OF_EXOGENOUS_ANTIGEN | -0.840456735 | -2.688328377 | 1.00E-10 | 2.75E-09 |
| GOBP_POSITIVE_REGULATION_OF_LYMPHOCYTE_DIFFERENTIATION | -0.725112011 | -2.685055781 | 1.00E-10 | 2.75E-09 |
| GOBP_REGULATION_OF_CD4_POSITIVE_ALPHA_BETA_T_CELL_DIFFERENTIATION | -0.813010877 | -2.683836009 | 1.00E-10 | 2.75E-09 |
| GOBP_NEUTROPHIL_CHEMOTAXIS | -0.750801226 | -2.683724024 | 1.00E-10 | 2.75E-09 |
| GOBP_POSITIVE_REGULATION_OF_B_CELL_ACTIVATION | -0.76481145 | -2.681005865 | 1.00E-10 | 2.75E-09 |
| GOBP_PRODUCTION_OF_MOLECULAR_MEDIATOR_OF_IMMUNE_RESPONSE | -0.674538179 | -2.678425014 | 1.00E-10 | 2.75E-09 |
| GOBP_POSITIVE_REGULATION_OF_CELL_KILLING | -0.782670828 | -2.677801923 | 1.00E-10 | 2.75E-09 |
| GOBP_NEGATIVE_REGULATION_OF_CELL_ACTIVATION | -0.680639439 | -2.671630992 | 1.00E-10 | 2.75E-09 |
| GOBP_NEUTROPHIL_MIGRATION | -0.730745784 | -2.671503721 | 1.00E-10 | 2.75E-09 |
| GOBP_REGULATION_OF_B_CELL_PROLIFERATION | -0.783335357 | -2.661902082 | 1.00E-10 | 2.75E-09 |
| GOBP_CD4_POSITIVE_ALPHA_BETA_T_CELL_ACTIVATION | -0.737198161 | -2.66130615 | 1.00E-10 | 2.75E-09 |
| GOBP_REGULATION_OF_T_CELL_DIFFERENTIATION | -0.692742126 | -2.65922583 | 1.00E-10 | 2.75E-09 |
| GOBP_POSITIVE_REGULATION_OF_LEUKOCYTE_PROLIFERATION | -0.699881122 | -2.656345705 | 1.00E-10 | 2.75E-09 |
| GOBP_POSITIVE_REGULATION_OF_RESPONSE_TO_BIOTIC_STIMULUS | -0.644565529 | -2.653293528 | 1.00E-10 | 2.75E-09 |
| GOBP_POSITIVE_REGULATION_OF_T_CELL_MEDIATED_IMMUNITY | -0.783493833 | -2.648226431 | 1.00E-10 | 2.75E-09 |
| GOBP_CELL_KILLING | -0.689131426 | -2.646097217 | 1.00E-10 | 2.75E-09 |
| GOBP_NEGATIVE_REGULATION_OF_IMMUNE_RESPONSE | -0.686152279 | -2.646016081 | 1.00E-10 | 2.75E-09 |
| GOBP_REGULATION_OF_LYMPHOCYTE_DIFFERENTIATION | -0.674719418 | -2.644631039 | 1.00E-10 | 2.75E-09 |
| GOBP_POSITIVE_REGULATION_OF_DEFENSE_RESPONSE | -0.623452272 | -2.640735918 | 1.00E-10 | 2.75E-09 |
| GOBP_POSITIVE_REGULATION_OF_ALPHA_BETA_T_CELL_DIFFERENTIATION | -0.80345908 | -2.638364893 | 1.00E-10 | 2.75E-09 |
| GOBP_POSITIVE_REGULATION_OF_T_CELL_PROLIFERATION | -0.738312105 | -2.633531236 | 1.00E-10 | 2.75E-09 |
| GOMF_ANTIGEN_BINDING | -0.788373041 | -2.633050071 | 1.00E-10 | 2.75E-09 |
| GOBP_REGULATION_OF_LEUKOCYTE_DIFFERENTIATION | -0.636253393 | -2.627162655 | 1.00E-10 | 2.75E-09 |
| GOBP_GRANULOCYTE_MIGRATION | -0.697946914 | -2.62657593 | 1.00E-10 | 2.75E-09 |
| GOBP_GRANULOCYTE_CHEMOTAXIS | -0.716427272 | -2.617395391 | 1.00E-10 | 2.75E-09 |
| GOBP_INTERLEUKIN_10_PRODUCTION | -0.783471637 | -2.616367447 | 1.00E-10 | 2.75E-09 |
| GOBP_REGULATION_OF_CELL_KILLING | -0.726421188 | -2.615861551 | 1.00E-10 | 2.75E-09 |
| GOBP_NEGATIVE_REGULATION_OF_IMMUNE_SYSTEM_PROCESS | -0.61070566 | -2.615332924 | 1.00E-10 | 2.75E-09 |
| GOBP_T_CELL_DIFFERENTIATION_INVOLVED_IN_IMMUNE_RESPONSE | -0.745237243 | -2.614653943 | 1.00E-10 | 2.75E-09 |
| GOBP_POSITIVE_REGULATION_OF_HEMOPOIESIS | -0.677306119 | -2.612317331 | 1.00E-10 | 2.75E-09 |
| GOBP_REGULATION_OF_CELL_CELL_ADHESION | -0.607934164 | -2.609999569 | 1.00E-10 | 2.75E-09 |
| GOBP_NEGATIVE_REGULATION_OF_INNATE_IMMUNE_RESPONSE | -0.746195532 | -2.60913844 | 1.00E-10 | 2.75E-09 |
| GOBP_NEGATIVE_REGULATION_OF_LYMPHOCYTE_ACTIVATION | -0.689697167 | -2.605551922 | 1.00E-10 | 2.75E-09 |
| GOBP_POSITIVE_T_CELL_SELECTION | -0.838655188 | -2.601813427 | 1.00E-10 | 2.75E-09 |
| GOBP_NEGATIVE_REGULATION_OF_LEUKOCYTE_MEDIATED_IMMUNITY | -0.752558534 | -2.597555285 | 1.00E-10 | 2.75E-09 |
| GOBP_REGULATION_OF_CD4_POSITIVE_ALPHA_BETA_T_CELL_ACTIVATION | -0.744570285 | -2.586990832 | 1.00E-10 | 2.75E-09 |
| GOBP_CELLULAR_RESPONSE_TO_TYPE_II_INTERFERON | -0.715364115 | -2.576044744 | 1.00E-10 | 2.75E-09 |
| GOBP_ANTIGEN_PROCESSING_AND_PRESENTATION_OF_EXOGENOUS_PEPTIDE_ANTIGEN | -0.856221966 | -2.575729772 | 1.00E-10 | 2.75E-09 |
| GOBP_T_HELPER_1_TYPE_IMMUNE_RESPONSE | -0.803901606 | -2.571401252 | 1.00E-10 | 2.75E-09 |
| GOBP_INTERLEUKIN_12_PRODUCTION | -0.753919737 | -2.569255892 | 1.00E-10 | 2.75E-09 |
| GOMF_IMMUNE_RECEPTOR_ACTIVITY | -0.692514523 | -2.568568727 | 1.00E-10 | 2.75E-09 |
| GOBP_IMMUNOGLOBULIN_PRODUCTION | -0.705006649 | -2.554514111 | 1.00E-10 | 2.75E-09 |
| GOBP_REGULATION_OF_T_CELL_RECEPTOR_SIGNALING_PATHWAY | -0.805708413 | -2.552515464 | 1.00E-10 | 2.75E-09 |
| GOBP_REGULATION_OF_PRODUCTION_OF_MOLECULAR_MEDIATOR_OF_IMMUNE_RESPONSE | -0.662409966 | -2.548860526 | 1.00E-10 | 2.75E-09 |
| GOMF_CHEMOKINE_RECEPTOR_BINDING | -0.772664337 | -2.547760776 | 1.00E-10 | 2.75E-09 |
| GOBP_PHAGOCYTOSIS | -0.641870894 | -2.543885686 | 1.00E-10 | 2.75E-09 |
| GOBP_CYTOKINE_PRODUCTION_INVOLVED_IN_IMMUNE_RESPONSE | -0.694704121 | -2.541963971 | 1.00E-10 | 2.75E-09 |
| GOBP_RESPONSE_TO_VIRUS | -0.601746734 | -2.539639074 | 1.00E-10 | 2.75E-09 |
| GOBP_IMMUNOGLOBULIN_PRODUCTION_INVOLVED_IN_IMMUNOGLOBULIN_MEDIATED_IMMUNE_RESPONSE | -0.743990021 | -2.53541677 | 1.00E-10 | 2.75E-09 |
| GOBP_MYELOID_CELL_ACTIVATION_INVOLVED_IN_IMMUNE_RESPONSE | -0.709063181 | -2.534342017 | 1.00E-10 | 2.75E-09 |
| GOBP_T_CELL_MEDIATED_CYTOTOXICITY | -0.767241326 | -2.530751438 | 1.00E-10 | 2.75E-09 |
| GOBP_CYTOKINE_MEDIATED_SIGNALING_PATHWAY | -0.589024384 | -2.528388754 | 1.00E-10 | 2.75E-09 |
| GOBP_RESPONSE_TO_MOLECULE_OF_BACTERIAL_ORIGIN | -0.607123431 | -2.524725297 | 1.00E-10 | 2.75E-09 |
| GOBP_INFLAMMATORY_RESPONSE_TO_ANTIGENIC_STIMULUS | -0.732989168 | -2.516058084 | 1.00E-10 | 2.75E-09 |
| GOBP_ANTIGEN_PROCESSING_AND_PRESENTATION_OF_PEPTIDE_OR_POLYSACCHARIDE_ANTIGEN_VIA_MHC_CLASS_II | -0.865631441 | -2.514584488 | 1.00E-10 | 2.75E-09 |
| GOBP_INTERFERON_MEDIATED_SIGNALING_PATHWAY | -0.700702326 | -2.512164993 | 1.00E-10 | 2.75E-09 |
| GOBP_CELLULAR_RESPONSE_TO_VIRUS | -0.71819021 | -2.511215365 | 1.00E-10 | 2.75E-09 |
| GOCC_EXTERNAL_SIDE_OF_PLASMA_MEMBRANE | -0.600808067 | -2.504719383 | 1.00E-10 | 2.75E-09 |
| GOBP_INNATE_IMMUNE_RESPONSE_ACTIVATING_SIGNALING_PATHWAY | -0.642926718 | -2.50236459 | 1.00E-10 | 2.75E-09 |
| GOBP_ACTIVATION_OF_INNATE_IMMUNE_RESPONSE | -0.633567037 | -2.497796625 | 1.00E-10 | 2.75E-09 |
| GOBP_INTERLEUKIN_6_PRODUCTION | -0.664759082 | -2.496317142 | 1.00E-10 | 2.75E-09 |
| GOBP_TYPE_I_INTERFERON_PRODUCTION | -0.695508267 | -2.4935432 | 1.00E-10 | 2.75E-09 |
| GOBP_LEUKOCYTE_CHEMOTAXIS | -0.628223649 | -2.489798436 | 1.00E-10 | 2.75E-09 |
| GOBP_LEUKOCYTE_DEGRANULATION | -0.70801529 | -2.484061267 | 1.00E-10 | 2.75E-09 |
| GOBP_POSITIVE_REGULATION_OF_PRODUCTION_OF_MOLECULAR_MEDIATOR_OF_IMMUNE_RESPONSE | -0.674759745 | -2.480054505 | 1.00E-10 | 2.75E-09 |
| GOBP_POSITIVE_REGULATION_OF_PHAGOCYTOSIS | -0.717379064 | -2.476128693 | 1.00E-10 | 2.75E-09 |
| GOBP_NEGATIVE_REGULATION_OF_LEUKOCYTE_PROLIFERATION | -0.695917567 | -2.475743325 | 1.00E-10 | 2.75E-09 |
| GOBP_NEGATIVE_REGULATION_OF_IMMUNE_EFFECTOR_PROCESS | -0.675694358 | -2.475478008 | 1.00E-10 | 2.75E-09 |
| GOBP_REGULATION_OF_PHAGOCYTOSIS | -0.689755836 | -2.473028812 | 1.00E-10 | 2.75E-09 |
| GOBP_POSITIVE_REGULATION_OF_INTERLEUKIN_4_PRODUCTION | -0.887797497 | -2.463659279 | 1.00E-10 | 2.75E-09 |
| GOBP_CELLULAR_RESPONSE_TO_BIOTIC_STIMULUS | -0.616550017 | -2.460588803 | 1.00E-10 | 2.75E-09 |
| GOBP_REGULATION_OF_HEMOPOIESIS | -0.583258306 | -2.459800468 | 1.00E-10 | 2.75E-09 |
| GOBP_REGULATION_OF_LEUKOCYTE_APOPTOTIC_PROCESS | -0.696135517 | -2.456241712 | 1.00E-10 | 2.75E-09 |
| GOBP_CELLULAR_RESPONSE_TO_MOLECULE_OF_BACTERIAL_ORIGIN | -0.625464995 | -2.452176165 | 1.00E-10 | 2.75E-09 |
| GOBP_RESPONSE_TO_CHEMOKINE | -0.687229212 | -2.449234641 | 1.00E-10 | 2.75E-09 |
| GOBP_LEUKOCYTE_APOPTOTIC_PROCESS | -0.669558389 | -2.438163741 | 1.00E-10 | 2.75E-09 |
| GOBP_LEUKOCYTE_HOMEOSTASIS | -0.681357858 | -2.435132545 | 1.00E-10 | 2.75E-09 |
| GOBP_TUMOR_NECROSIS_FACTOR_SUPERFAMILY_CYTOKINE_PRODUCTION | -0.642157514 | -2.433181559 | 1.00E-10 | 2.75E-09 |
| GOBP_MYELOID_LEUKOCYTE_MIGRATION | -0.613830563 | -2.432857307 | 1.00E-10 | 2.75E-09 |
| GOBP_POSITIVE_REGULATION_OF_CELL_ADHESION | -0.565350099 | -2.427176496 | 1.00E-10 | 2.75E-09 |
| GOCC_MHC_PROTEIN_COMPLEX | -0.920630521 | -2.422492959 | 1.00E-10 | 2.75E-09 |
| GOBP_LEUKOCYTE_MIGRATION | -0.572336957 | -2.415516725 | 1.00E-10 | 2.75E-09 |
| GOBP_DEFENSE_RESPONSE_TO_SYMBIONT | -0.589628994 | -2.409441747 | 1.00E-10 | 2.75E-09 |
| GOBP_NEGATIVE_REGULATION_OF_CYTOKINE_PRODUCTION | -0.590583535 | -2.407986458 | 1.00E-10 | 2.75E-09 |
| GOBP_MACROPHAGE_ACTIVATION | -0.67068389 | -2.404648858 | 1.00E-10 | 2.75E-09 |
| GOBP_REGULATION_OF_INFLAMMATORY_RESPONSE | -0.57341146 | -2.403526627 | 1.00E-10 | 2.75E-09 |
| GOBP_LYMPHOCYTE_MIGRATION | -0.663757259 | -2.396182422 | 1.00E-10 | 2.75E-09 |
| GOBP_MONONUCLEAR_CELL_MIGRATION | -0.616005934 | -2.382777607 | 1.00E-10 | 2.75E-09 |
| GOBP_NEGATIVE_REGULATION_OF_LEUKOCYTE_CELL_CELL_ADHESION | -0.642024255 | -2.381297963 | 1.00E-10 | 2.75E-09 |
| GOBP_INTERLEUKIN_1_PRODUCTION | -0.659311162 | -2.380131885 | 1.00E-10 | 2.75E-09 |
| GOBP_B_CELL_DIFFERENTIATION | -0.632321044 | -2.378135223 | 1.00E-10 | 2.75E-09 |
| GOBP_MYELOID_LEUKOCYTE_MEDIATED_IMMUNITY | -0.657333383 | -2.367922283 | 1.00E-10 | 2.75E-09 |
| GOBP_NEGATIVE_REGULATION_OF_DEFENSE_RESPONSE | -0.591477924 | -2.360528612 | 1.00E-10 | 2.75E-09 |
| GOBP_CELL_CHEMOTAXIS | -0.572607873 | -2.339933779 | 1.00E-10 | 2.75E-09 |
| GOMF_CYTOKINE_RECEPTOR_BINDING | -0.585629177 | -2.338928195 | 1.00E-10 | 2.75E-09 |
| GOBP_HUMORAL_IMMUNE_RESPONSE | -0.58901726 | -2.334675125 | 1.00E-10 | 2.75E-09 |
| GOBP_NEGATIVE_REGULATION_OF_RESPONSE_TO_BIOTIC_STIMULUS | -0.636524494 | -2.312937528 | 1.00E-10 | 2.75E-09 |
| GOBP_POSITIVE_REGULATION_OF_INFLAMMATORY_RESPONSE | -0.615748972 | -2.302645074 | 1.00E-10 | 2.75E-09 |
| GOBP_NIK_NF_KAPPAB_SIGNALING | -0.623818777 | -2.28542631 | 1.00E-10 | 2.75E-09 |
| GOBP_RESPONSE_TO_TUMOR_NECROSIS_FACTOR | -0.563770368 | -2.245193206 | 1.00E-10 | 2.75E-09 |
| GOCC_TERTIARY_GRANULE | -0.589348991 | -2.229644367 | 1.00E-10 | 2.75E-09 |
| GOBP_REGULATION_OF_LEUKOCYTE_MIGRATION | -0.558572167 | -2.211109904 | 1.00E-10 | 2.75E-09 |
| GOCC_FICOLIN_1_RICH_GRANULE | -0.573593575 | -2.207107526 | 1.00E-10 | 2.75E-09 |
| GOBP_KIDNEY_EPITHELIUM_DEVELOPMENT | 0.5868632 | 2.205838966 | 1.00E-10 | 2.75E-09 |
| GOMF_CYTOKINE_ACTIVITY | -0.551089972 | -2.180136768 | 1.00E-10 | 2.75E-09 |
| GOBP_MYELOID_LEUKOCYTE_DIFFERENTIATION | -0.547435363 | -2.173178172 | 1.00E-10 | 2.75E-09 |
| GOBP_NEGATIVE_REGULATION_OF_RESPONSE_TO_EXTERNAL_STIMULUS | -0.506543303 | -2.140830248 | 1.00E-10 | 2.75E-09 |
| GOBP_DEFENSE_RESPONSE_TO_BACTERIUM | -0.51474371 | -2.073484947 | 1.00E-10 | 2.75E-09 |
| GOCC_ENDOCYTIC_VESICLE | -0.494496236 | -2.056995023 | 1.00E-10 | 2.75E-09 |
| GOBP_POSITIVE_REGULATION_OF_CELL_DEVELOPMENT | -0.478946814 | -2.043837227 | 1.00E-10 | 2.75E-09 |
| GOCC_SECRETORY_GRANULE_MEMBRANE | -0.494134244 | -2.036027046 | 1.00E-10 | 2.75E-09 |
| GOCC_VESICLE_LUMEN | -0.492275581 | -2.0314105 | 1.00E-10 | 2.75E-09 |
| GOBP_MUSCLE_TISSUE_DEVELOPMENT | 0.475390739 | 1.989598387 | 1.00E-10 | 2.75E-09 |
| GOBP_MYELOID_CELL_DIFFERENTIATION | -0.463486048 | -1.954679747 | 1.00E-10 | 2.75E-09 |
| GOBP_CELL_JUNCTION_ASSEMBLY | 0.457358747 | 1.925974511 | 1.00E-10 | 2.75E-09 |
| GOBP_CELL_CELL_SIGNALING_BY_WNT | 0.454226204 | 1.911148393 | 1.00E-10 | 2.75E-09 |
| GOBP_REGULATION_OF_VESICLE_MEDIATED_TRANSPORT | -0.42697241 | -1.844802129 | 1.00E-10 | 2.75E-09 |
| GOBP_LYMPHOCYTE_HOMEOSTASIS | -0.71756577 | -2.509031956 | 1.06E-10 | 2.91E-09 |
| GOMF_CCR_CHEMOKINE_RECEPTOR_BINDING | -0.848548155 | -2.498128662 | 1.29E-10 | 3.50E-09 |
| GOBP_I_KAPPAB_KINASE_NF_KAPPAB_SIGNALING | -0.499128676 | -2.03387713 | 1.55E-10 | 4.20E-09 |
| GOBP_POSITIVE_REGULATION_OF_TUMOR_NECROSIS_FACTOR_SUPERFAMILY_CYTOKINE_PRODUCTION | -0.656704189 | -2.338490173 | 1.66E-10 | 4.48E-09 |
| GOBP_ANTIGEN_PROCESSING_AND_PRESENTATION_OF_EXOGENOUS_PEPTIDE_ANTIGEN_VIA_MHC_CLASS_II | -0.874564645 | -2.452002085 | 1.70E-10 | 4.56E-09 |
| GOBP_T_CELL_DIFFERENTIATION_IN_THYMUS | -0.681848676 | -2.404910292 | 1.96E-10 | 5.22E-09 |
| GOBP_RENAL_SYSTEM_DEVELOPMENT | 0.476639212 | 1.95977652 | 2.14E-10 | 5.66E-09 |
| GOBP_POSITIVE_REGULATION_OF_CD4_POSITIVE_ALPHA_BETA_T_CELL_ACTIVATION | -0.803846512 | -2.514615996 | 2.88E-10 | 7.60E-09 |
| GOBP_POSITIVE_REGULATION_OF_CD4_POSITIVE_ALPHA_BETA_T_CELL_DIFFERENTIATION | -0.838431427 | -2.479762372 | 2.95E-10 | 7.73E-09 |
| GOBP_HOMEOSTASIS_OF_NUMBER_OF_CELLS | -0.484358389 | -1.988944989 | 3.10E-10 | 8.09E-09 |
| GOBP_IMMUNE_SYSTEM_DEVELOPMENT | -0.546860162 | -2.115159459 | 3.40E-10 | 8.80E-09 |
| GOBP_REGULATION_OF_IMMUNOGLOBULIN_PRODUCTION | -0.704048428 | -2.43011624 | 3.70E-10 | 9.48E-09 |
| GOBP_REGULATION_OF_LEUKOCYTE_CHEMOTAXIS | -0.614221894 | -2.247474685 | 3.69E-10 | 9.48E-09 |
| GOBP_REGULATION_OF_VIRAL_PROCESS | -0.574503864 | -2.1587987 | 3.88E-10 | 9.88E-09 |
| GOCC_IMMUNOLOGICAL_SYNAPSE | -0.801479035 | -2.507209984 | 3.91E-10 | 9.92E-09 |
| GOCC_PHAGOCYTIC_VESICLE | -0.5971232 | -2.225534063 | 4.06E-10 | 1.02E-08 |
| GOCC_PLASMA_MEMBRANE_SIGNALING_RECEPTOR_COMPLEX | -0.547033964 | -2.117362741 | 4.12E-10 | 1.04E-08 |
| GOCC_APICAL_JUNCTION_COMPLEX | 0.589768474 | 2.189238733 | 6.03E-10 | 1.51E-08 |
| GOBP_DENDRITIC_CELL_DIFFERENTIATION | -0.776669466 | -2.484295121 | 8.31E-10 | 2.07E-08 |
| GOBP_NATURAL_KILLER_CELL_ACTIVATION | -0.655777438 | -2.351099836 | 9.58E-10 | 2.37E-08 |
| GOMF_MHC_PROTEIN_COMPLEX_BINDING | -0.849170989 | -2.432277039 | 9.69E-10 | 2.38E-08 |
| GOBP_NEGATIVE_REGULATION_OF_CELL_CELL_ADHESION | -0.546785239 | -2.115024472 | 1.02E-09 | 2.51E-08 |
| GOBP_VIRAL_PROCESS | -0.441815607 | -1.871442672 | 1.14E-09 | 2.79E-08 |
| GOBP_DNA_RECOMBINATION | -0.479172649 | -1.967214916 | 1.18E-09 | 2.87E-08 |
| GOBP_INTERLEUKIN_2_PRODUCTION | -0.718438111 | -2.413115757 | 1.21E-09 | 2.92E-08 |
| GOBP_POSITIVE_REGULATION_OF_CYTOKINE_PRODUCTION_INVOLVED_IN_IMMUNE_RESPONSE | -0.695167984 | -2.4368744 | 1.22E-09 | 2.93E-08 |
| GOBP_CHRONIC_INFLAMMATORY_RESPONSE | -0.896675731 | -2.339570529 | 1.24E-09 | 2.96E-08 |
| GOBP_REGULATION_OF_T_CELL_MEDIATED_CYTOTOXICITY | -0.781505075 | -2.475838349 | 1.27E-09 | 3.01E-08 |
| GOBP_GLIAL_CELL_ACTIVATION | -0.758788035 | -2.463707968 | 1.28E-09 | 3.03E-08 |
| GOBP_ANTIGEN_PROCESSING_AND_PRESENTATION_OF_ENDOGENOUS_ANTIGEN | -0.846072175 | -2.423401119 | 1.33E-09 | 3.13E-08 |
| GOBP_LYMPHOCYTE_COSTIMULATION | -0.763893298 | -2.462942112 | 1.35E-09 | 3.14E-08 |
| GOBP_NEGATIVE_REGULATION_OF_LYMPHOCYTE_MEDIATED_IMMUNITY | -0.735876907 | -2.438431601 | 1.35E-09 | 3.14E-08 |
| GOBP_PRODUCTION_OF_MOLECULAR_MEDIATOR_INVOLVED_IN_INFLAMMATORY_RESPONSE | -0.669628523 | -2.34955867 | 1.56E-09 | 3.61E-08 |
| GOCC_LUMENAL_SIDE_OF_MEMBRANE | -0.844029648 | -2.44036868 | 1.59E-09 | 3.68E-08 |
| GOBP_NEGATIVE_REGULATION_OF_ANTIGEN_RECEPTOR_MEDIATED_SIGNALING_PATHWAY | -0.817038162 | -2.420109201 | 1.62E-09 | 3.72E-08 |
| GOBP_MONOCYTE_CHEMOTAXIS | -0.714956946 | -2.401423095 | 1.69E-09 | 3.85E-08 |
| GOBP_TOLL_LIKE_RECEPTOR_SIGNALING_PATHWAY | -0.714623276 | -2.433304451 | 1.86E-09 | 4.23E-08 |
| GOCC_LUMENAL_SIDE_OF_ENDOPLASMIC_RETICULUM_MEMBRANE | -0.872556496 | -2.394197076 | 1.89E-09 | 4.29E-08 |
| GOBP_NEGATIVE_REGULATION_OF_INTERLEUKIN_6_PRODUCTION | -0.755350024 | -2.452545094 | 2.02E-09 | 4.56E-08 |
| GOBP_DENDRITIC_CELL_MIGRATION | -0.814711485 | -2.413217463 | 2.05E-09 | 4.59E-08 |
| GOBP_REGULATION_OF_MYELOID_LEUKOCYTE_MEDIATED_IMMUNITY | -0.721277002 | -2.410916672 | 2.20E-09 | 4.91E-08 |
| GOBP_INTERLEUKIN_4_PRODUCTION | -0.825727479 | -2.430944514 | 2.30E-09 | 5.12E-08 |
| GOBP_REGULATION_OF_T_HELPER_CELL_DIFFERENTIATION | -0.792945945 | -2.463261957 | 2.49E-09 | 5.51E-08 |
| GOCC_MHC_CLASS_II_PROTEIN_COMPLEX | -0.949216396 | -2.242100453 | 2.65E-09 | 5.85E-08 |
| GOCC_ENDOCYTIC_VESICLE_MEMBRANE | -0.53259681 | -2.066373138 | 2.77E-09 | 6.09E-08 |
| GOBP_ACTOMYOSIN_STRUCTURE_ORGANIZATION | 0.52229691 | 2.038610416 | 2.96E-09 | 6.47E-08 |
| GOBP_ANTIGEN_PROCESSING_AND_PRESENTATION_OF_ENDOGENOUS_PEPTIDE_ANTIGEN | -0.885168499 | -2.329180282 | 3.10E-09 | 6.75E-08 |
| GOBP_HEART_MORPHOGENESIS | 0.489444049 | 1.959360302 | 3.30E-09 | 7.14E-08 |
| GOBP_REGULATION_OF_T_HELPER_1_TYPE_IMMUNE_RESPONSE | -0.833627423 | -2.421615583 | 3.32E-09 | 7.17E-08 |
| GOBP_TOLERANCE_INDUCTION | -0.838242854 | -2.400975624 | 3.43E-09 | 7.36E-08 |
| GOBP_T_CELL_CYTOKINE_PRODUCTION | -0.790698888 | -2.453035543 | 4.59E-09 | 9.80E-08 |
| GOBP_NEGATIVE_REGULATION_OF_ADAPTIVE_IMMUNE_RESPONSE | -0.712555732 | -2.379831403 | 4.63E-09 | 9.85E-08 |
| GOBP_DENDRITIC_CELL_CHEMOTAXIS | -0.849899379 | -2.382848498 | 4.76E-09 | 1.01E-07 |
| GOMF_CYTOKINE_RECEPTOR_ACTIVITY | -0.637118217 | -2.271149099 | 4.81E-09 | 1.02E-07 |
| GOMF_CHEMOKINE_ACTIVITY | -0.780917216 | -2.442887906 | 5.06E-09 | 1.05E-07 |
| GOBP_NEGATIVE_REGULATION_OF_CELL_KILLING | -0.835611288 | -2.39343804 | 5.02E-09 | 1.05E-07 |
| GOBP_CALCIUM_ION_TRANSPORT | -0.426623505 | -1.814364372 | 5.06E-09 | 1.05E-07 |
| GOBP_NEUROINFLAMMATORY_RESPONSE | -0.705464968 | -2.402120257 | 5.16E-09 | 1.07E-07 |
| GOBP_NEGATIVE_REGULATION_OF_INFLAMMATORY_RESPONSE | -0.565327766 | -2.118804875 | 5.23E-09 | 1.07E-07 |
| GOBP_REGULATION_OF_RESPONSE_TO_CYTOKINE_STIMULUS | -0.562276721 | -2.11470121 | 5.22E-09 | 1.07E-07 |
| GOBP_RECEPTOR_SIGNALING_PATHWAY_VIA_STAT | -0.545351621 | -2.08426806 | 5.21E-09 | 1.07E-07 |
| GOCC_VACUOLAR_MEMBRANE | -0.425256118 | -1.810375726 | 5.43E-09 | 1.11E-07 |
| GOBP_REGULATORY_T_CELL_DIFFERENTIATION | -0.795832928 | -2.394064446 | 5.46E-09 | 1.11E-07 |
| GOBP_CANONICAL_WNT_SIGNALING_PATHWAY | 0.467226957 | 1.89561309 | 6.43E-09 | 1.30E-07 |
| GOBP_LEUKOCYTE_ACTIVATION_INVOLVED_IN_INFLAMMATORY_RESPONSE | -0.756705711 | -2.420438021 | 6.89E-09 | 1.39E-07 |
| GOBP_MAST_CELL_ACTIVATION | -0.682810119 | -2.381211802 | 6.93E-09 | 1.39E-07 |
| GOBP_POSITIVE_REGULATION_OF_LEUKOCYTE_MIGRATION | -0.574036253 | -2.146270459 | 7.14E-09 | 1.43E-07 |
| GOBP_INTERLEUKIN_17_PRODUCTION | -0.784465118 | -2.405451387 | 7.68E-09 | 1.53E-07 |
| GOMF_PEPTIDE_ANTIGEN_BINDING | -0.846294551 | -2.372741701 | 7.74E-09 | 1.53E-07 |
| GOCC_T_CELL_RECEPTOR_COMPLEX | -0.899620214 | -2.340473086 | 8.13E-09 | 1.60E-07 |
| GOBP_REGULATION_OF_NATURAL_KILLER_CELL_MEDIATED_IMMUNITY | -0.753194293 | -2.443370558 | 8.41E-09 | 1.65E-07 |
| GOCC_CELL_CELL_JUNCTION | 0.413077804 | 1.754522961 | 8.78E-09 | 1.72E-07 |
| GOBP_POSITIVE_REGULATION_OF_NF_KAPPAB_TRANSCRIPTION_FACTOR_ACTIVITY | -0.548207534 | -2.059985644 | 8.88E-09 | 1.73E-07 |
| GOBP_INNATE_IMMUNE_RESPONSE_ACTIVATING_CELL_SURFACE_RECEPTOR_SIGNALING_PATHWAY | -0.679692638 | -2.37660521 | 9.06E-09 | 1.76E-07 |
| GOBP_B_CELL_ACTIVATION_INVOLVED_IN_IMMUNE_RESPONSE | -0.659010893 | -2.319873821 | 9.75E-09 | 1.88E-07 |
| GOCC_TERTIARY_GRANULE_MEMBRANE | -0.678428588 | -2.372185347 | 1.06E-08 | 2.03E-07 |
| GOBP_POSITIVE_REGULATION_OF_INTERLEUKIN_6_PRODUCTION | -0.640495764 | -2.293339812 | 1.09E-08 | 2.09E-07 |
| GOCC_TIGHT_JUNCTION | 0.570186815 | 2.09139752 | 1.13E-08 | 2.15E-07 |
| GOBP_PEPTIDE_ANTIGEN_ASSEMBLY_WITH_MHC_CLASS_II_PROTEIN_COMPLEX | -0.947331688 | -2.19120046 | 1.19E-08 | 2.27E-07 |
| GOCC_SPECIFIC_GRANULE | -0.55159937 | -2.086577055 | 1.25E-08 | 2.37E-07 |
| GOBP_REGULATION_OF_CALCIUM_ION_TRANSPORT | -0.484266998 | -1.941212601 | 1.43E-08 | 2.70E-07 |
| GOBP_POSITIVE_REGULATION_OF_B_CELL_PROLIFERATION | -0.760340055 | -2.408786747 | 1.56E-08 | 2.94E-07 |
| GOBP_REGULATION_OF_ENDOPEPTIDASE_ACTIVITY | -0.454655943 | -1.873196545 | 1.62E-08 | 3.03E-07 |
| GOBP_POSITIVE_REGULATION_OF_TYPE_I_INTERFERON_PRODUCTION | -0.708031921 | -2.364439989 | 1.67E-08 | 3.12E-07 |
| GOCC_SPECIFIC_GRANULE_MEMBRANE | -0.633560222 | -2.249577401 | 2.50E-08 | 4.64E-07 |
| GOBP_PEPTIDE_ANTIGEN_ASSEMBLY_WITH_MHC_PROTEIN_COMPLEX | -0.912046905 | -2.254787533 | 2.57E-08 | 4.76E-07 |
| GOBP_CELLULAR_RESPONSE_TO_INTERLEUKIN_1 | -0.607275928 | -2.170888334 | 2.60E-08 | 4.79E-07 |
| GOBP_MAINTENANCE_OF_LOCATION | -0.440893842 | -1.825134222 | 2.60E-08 | 4.79E-07 |
| GOBP_REGULATION_OF_B_CELL_MEDIATED_IMMUNITY | -0.706669187 | -2.341647714 | 2.65E-08 | 4.85E-07 |
| GOBP_REGULATION_OF_INFLAMMATORY_RESPONSE_TO_ANTIGENIC_STIMULUS | -0.771402495 | -2.396337897 | 2.72E-08 | 4.94E-07 |
| GOBP_POSITIVE_REGULATION_OF_LEUKOCYTE_CHEMOTAXIS | -0.631598485 | -2.261482483 | 2.72E-08 | 4.94E-07 |
| GOBP_CD8_POSITIVE_ALPHA_BETA_T_CELL_ACTIVATION | -0.838115882 | -2.325791608 | 2.73E-08 | 4.95E-07 |
| GOBP_POSITIVE_REGULATION_OF_T_CELL_MEDIATED_CYTOTOXICITY | -0.795539369 | -2.377843425 | 3.01E-08 | 5.42E-07 |
| GOBP_NEPHRON_EPITHELIUM_DEVELOPMENT | 0.56997812 | 2.072866169 | 3.00E-08 | 5.42E-07 |
| GOBP_INTERLEUKIN_8_PRODUCTION | -0.640080158 | -2.257590893 | 3.08E-08 | 5.52E-07 |
| GOBP_REGULATION_OF_DEFENSE_RESPONSE_TO_VIRUS_BY_HOST | -0.779506117 | -2.344949317 | 3.09E-08 | 5.52E-07 |
| GOBP_REGULATION_OF_CHEMOTAXIS | -0.497564527 | -1.968387695 | 3.30E-08 | 5.86E-07 |
| GOBP_LIPOPOLYSACCHARIDE_MEDIATED_SIGNALING_PATHWAY | -0.679930089 | -2.298178692 | 3.58E-08 | 6.34E-07 |
| GOBP_LYMPHOCYTE_CHEMOTAXIS | -0.732968089 | -2.41301789 | 3.67E-08 | 6.47E-07 |
| GOBP_POSITIVE_THYMIC_T_CELL_SELECTION | -0.921664925 | -2.222002625 | 3.88E-08 | 6.82E-07 |
| GOBP_NEGATIVE_REGULATION_OF_CELL_ADHESION | -0.457958703 | -1.871228803 | 3.92E-08 | 6.87E-07 |
| GOCC_ENDOSOME_MEMBRANE | -0.395449405 | -1.709152687 | 4.28E-08 | 7.48E-07 |
| GOBP_NEPHRON_DEVELOPMENT | 0.533261489 | 2.01950862 | 4.30E-08 | 7.48E-07 |
| GOBP_NEGATIVE_REGULATION_OF_T_CELL_PROLIFERATION | -0.67445304 | -2.307549461 | 4.40E-08 | 7.60E-07 |
| GOCC_CHROMOSOMAL_REGION | -0.43020686 | -1.79421565 | 4.40E-08 | 7.60E-07 |
| GOBP_THYMIC_T_CELL_SELECTION | -0.857985124 | -2.309055771 | 4.51E-08 | 7.76E-07 |
| GOBP_REGULATION_OF_LEUKOCYTE_DEGRANULATION | -0.730210533 | -2.36881099 | 5.25E-08 | 9.00E-07 |
| GOBP_POSITIVE_REGULATION_OF_B_CELL_MEDIATED_IMMUNITY | -0.789683814 | -2.360341344 | 5.39E-08 | 9.21E-07 |
| GOBP_POSITIVE_REGULATION_OF_INTERLEUKIN_10_PRODUCTION | -0.76448444 | -2.374847175 | 5.68E-08 | 9.68E-07 |
| GOBP_REGULATION_OF_PEPTIDASE_ACTIVITY | -0.421060743 | -1.779780892 | 5.78E-08 | 9.82E-07 |
| GOBP_LYMPHOCYTE_APOPTOTIC_PROCESS | -0.634902192 | -2.227712676 | 5.92E-08 | 1.00E-06 |
| GOBP_RESPONSE_TO_INTERLEUKIN_1 | -0.567742881 | -2.094331275 | 6.29E-08 | 1.06E-06 |
| GOBP_RESPONSE_TO_TYPE_I_INTERFERON | -0.653761527 | -2.293466298 | 6.37E-08 | 1.07E-06 |
| GOBP_NEGATIVE_REGULATION_OF_TYPE_II_INTERFERON_PRODUCTION | -0.774932661 | -2.352866014 | 6.80E-08 | 1.14E-06 |
| GOBP_NEGATIVE_REGULATION_OF_CYTOKINE_PRODUCTION_INVOLVED_IN_IMMUNE_RESPONSE | -0.771155905 | -2.319829791 | 7.34E-08 | 1.22E-06 |
| GOBP_POSITIVE_REGULATION_OF_INTERLEUKIN_1_PRODUCTION | -0.665853625 | -2.285608666 | 7.49E-08 | 1.24E-06 |
| GOBP_T_HELPER_17_TYPE_IMMUNE_RESPONSE | -0.75970926 | -2.360013227 | 8.05E-08 | 1.33E-06 |
| GOMF_MHC_CLASS_II_PROTEIN_COMPLEX_BINDING | -0.854142682 | -2.298714784 | 8.61E-08 | 1.42E-06 |
| GOBP_NEGATIVE_REGULATION_OF_VIRAL_PROCESS | -0.632915538 | -2.236766171 | 8.79E-08 | 1.45E-06 |
| GOBP_POSITIVE_REGULATION_OF_INTERLEUKIN_17_PRODUCTION | -0.825479622 | -2.290725684 | 9.01E-08 | 1.48E-06 |
| GOBP_ANTIGEN_PROCESSING_AND_PRESENTATION_OF_PEPTIDE_ANTIGEN_VIA_MHC_CLASS_I | -0.779648463 | -2.309358983 | 9.08E-08 | 1.48E-06 |
| GOBP_MESONEPHROS_DEVELOPMENT | 0.582349383 | 2.087909044 | 9.30E-08 | 1.52E-06 |
| GOBP_REGULATION_OF_MONONUCLEAR_CELL_MIGRATION | -0.582012282 | -2.114856635 | 9.35E-08 | 1.52E-06 |
| GOBP_REGULATION_OF_WNT_SIGNALING_PATHWAY | 0.434855106 | 1.787145963 | 9.40E-08 | 1.52E-06 |
| GOCC_RECEPTOR_COMPLEX | -0.412938362 | -1.747534024 | 9.46E-08 | 1.53E-06 |
| GOBP_CHROMOSOME_SEGREGATION | -0.410234317 | -1.73890731 | 9.65E-08 | 1.55E-06 |
| GOBP_POSITIVE_REGULATION_OF_INTERLEUKIN_12_PRODUCTION | -0.743097565 | -2.354161871 | 9.84E-08 | 1.58E-06 |
| GOBP_GRANULOCYTE_ACTIVATION | -0.720378212 | -2.322640923 | 9.88E-08 | 1.58E-06 |
| GOBP_CELL_SURFACE_PATTERN_RECOGNITION_RECEPTOR_SIGNALING_PATHWAY | -0.69677589 | -2.297528432 | 1.02E-07 | 1.62E-06 |
| GOBP_NEGATIVE_REGULATION_OF_NATURAL_KILLER_CELL_MEDIATED_IMMUNITY | -0.858314374 | -2.239479609 | 1.20E-07 | 1.91E-06 |
| GOBP_BONE_RESORPTION | -0.665333402 | -2.267365713 | 1.22E-07 | 1.94E-06 |
| GOBP_MAINTENANCE_OF_LOCATION_IN_CELL | -0.484023538 | -1.9182994 | 1.26E-07 | 1.99E-06 |
| GOBP_ALPHA_BETA_T_CELL_PROLIFERATION | -0.726694474 | -2.324442527 | 1.27E-07 | 2.00E-06 |
| GOBP_NEGATIVE_REGULATION_OF_PRODUCTION_OF_MOLECULAR_MEDIATOR_OF_IMMUNE_RESPONSE | -0.710530571 | -2.307020866 | 1.45E-07 | 2.26E-06 |
| GOBP_NEGATIVE_REGULATION_OF_TUMOR_NECROSIS_FACTOR_SUPERFAMILY_CYTOKINE_PRODUCTION | -0.683811892 | -2.283558318 | 1.45E-07 | 2.26E-06 |
| GOBP_REGULATION_OF_DNA_BINDING_TRANSCRIPTION_FACTOR_ACTIVITY | -0.404298976 | -1.721390059 | 1.44E-07 | 2.26E-06 |
| GOBP_POSITIVE_REGULATION_OF_ALPHA_BETA_T_CELL_PROLIFERATION | -0.819819849 | -2.275019679 | 1.61E-07 | 2.49E-06 |
| GOBP_POSITIVE_REGULATION_OF_I_KAPPAB_KINASE_NF_KAPPAB_SIGNALING | -0.499614539 | -1.92666868 | 1.86E-07 | 2.88E-06 |
| GOBP_INTERFERON_BETA_PRODUCTION | -0.688762491 | -2.271888394 | 1.98E-07 | 3.06E-06 |
| GOBP_NEGATIVE_REGULATION_OF_T_CELL_RECEPTOR_SIGNALING_PATHWAY | -0.81715405 | -2.267622024 | 2.06E-07 | 3.17E-06 |
| GOBP_REGULATION_OF_LYMPHOCYTE_APOPTOTIC_PROCESS | -0.67967924 | -2.269757517 | 2.11E-07 | 3.23E-06 |
| GOBP_INTRACELLULAR_MONOATOMIC_ION_HOMEOSTASIS | -0.386161537 | -1.661856661 | 2.12E-07 | 3.23E-06 |
| GOBP_AXIS_ELONGATION | 0.797765676 | 2.246530607 | 2.16E-07 | 3.30E-06 |
| GOBP_VIRAL_LIFE_CYCLE | -0.442483823 | -1.816879227 | 2.24E-07 | 3.40E-06 |
| GOBP_B_CELL_HOMEOSTASIS | -0.775172257 | -2.316966735 | 2.36E-07 | 3.57E-06 |
| GOBP_DEFENSE_RESPONSE_TO_GRAM_POSITIVE_BACTERIUM | -0.62224249 | -2.188471534 | 2.48E-07 | 3.74E-06 |
| GOBP_PATTERN_SPECIFICATION_PROCESS | 0.400503585 | 1.685542759 | 2.67E-07 | 4.01E-06 |
| GOBP_NUCLEAR_CHROMOSOME_SEGREGATION | -0.433331974 | -1.787200632 | 2.71E-07 | 4.06E-06 |
| GOBP_CELL_SURFACE_TOLL_LIKE_RECEPTOR_SIGNALING_PATHWAY | -0.713385484 | -2.314230344 | 2.80E-07 | 4.19E-06 |
| GOBP_NEGATIVE_REGULATION_OF_VIRAL_GENOME_REPLICATION | -0.702963061 | -2.308360324 | 2.86E-07 | 4.26E-06 |
| GOBP_REGULATION_OF_CANONICAL_WNT_SIGNALING_PATHWAY | 0.460983938 | 1.843001946 | 2.92E-07 | 4.34E-06 |
| GOBP_REGULATION_OF_NEUROINFLAMMATORY_RESPONSE | -0.78140786 | -2.300467405 | 2.96E-07 | 4.38E-06 |
| GOBP_BIOLOGICAL_PROCESS_INVOLVED_IN_SYMBIOTIC_INTERACTION | -0.436563807 | -1.792469629 | 2.96E-07 | 4.38E-06 |
| GOBP_STRIATED_MUSCLE_TISSUE_DEVELOPMENT | 0.470936605 | 1.876352145 | 2.98E-07 | 4.38E-06 |
| GOBP_POSITIVE_REGULATION_OF_TYPE_2_IMMUNE_RESPONSE | -0.866633687 | -2.228643268 | 3.33E-07 | 4.89E-06 |
| GOBP_REGULATION_OF_ANTIGEN_PROCESSING_AND_PRESENTATION | -0.864863161 | -2.25004832 | 3.55E-07 | 5.19E-06 |
| GOBP_REGULATION_OF_ANIMAL_ORGAN_MORPHOGENESIS | 0.543304123 | 2.017652065 | 3.68E-07 | 5.36E-06 |
| GOBP_ANTIGEN_PROCESSING_AND_PRESENTATION_OF_PEPTIDE_ANTIGEN_VIA_MHC_CLASS_IB | -0.894639958 | -2.195624265 | 3.74E-07 | 5.44E-06 |
| GOCC_CONTRACTILE_FIBER | 0.470855347 | 1.867882569 | 3.80E-07 | 5.51E-06 |
| GOBP_CELL_COMMUNICATION_INVOLVED_IN_CARDIAC_CONDUCTION | 0.662256102 | 2.160475177 | 4.10E-07 | 5.93E-06 |
| GOBP_POSITIVE_REGULATION_OF_MAPK_CASCADE | -0.38931491 | -1.671135299 | 4.19E-07 | 6.03E-06 |
| GOCC_I_BAND | 0.536612911 | 1.992506403 | 4.50E-07 | 6.46E-06 |
| GOBP_POSITIVE_REGULATION_OF_MONONUCLEAR_CELL_MIGRATION | -0.640570425 | -2.233906342 | 4.66E-07 | 6.67E-06 |
| GOCC_PHAGOCYTIC_VESICLE_MEMBRANE | -0.635620345 | -2.228133316 | 4.76E-07 | 6.79E-06 |
| GOBP_SEQUESTERING_OF_CALCIUM_ION | -0.545365254 | -2.011463031 | 4.86E-07 | 6.92E-06 |
| GOBP_REGULATION_OF_MACROPHAGE_ACTIVATION | -0.707157884 | -2.328047634 | 5.07E-07 | 7.20E-06 |
| GOBP_MAST_CELL_MEDIATED_IMMUNITY | -0.671890616 | -2.233184398 | 5.40E-07 | 7.65E-06 |
| GOMF_G_PROTEIN_COUPLED_CHEMOATTRACTANT_RECEPTOR_ACTIVITY | -0.802358945 | -2.226565253 | 5.75E-07 | 8.12E-06 |
| GOBP_MYELOID_DENDRITIC_CELL_ACTIVATION | -0.801887339 | -2.268977613 | 5.84E-07 | 8.22E-06 |
| GOBP_REGULATED_EXOCYTOSIS | -0.471216367 | -1.86754156 | 5.91E-07 | 8.30E-06 |
| GOBP_NEGATIVE_REGULATION_OF_INTERLEUKIN_10_PRODUCTION | -0.840442612 | -2.19284932 | 6.08E-07 | 8.48E-06 |
| GOCC_ADHERENS_JUNCTION | 0.49821223 | 1.925093185 | 6.06E-07 | 8.48E-06 |
| GOMF_TUMOR_NECROSIS_FACTOR_RECEPTOR_BINDING | -0.787314002 | -2.255100319 | 6.10E-07 | 8.50E-06 |
| GOBP_GAMMA_DELTA_T_CELL_ACTIVATION | -0.83733817 | -2.203322371 | 6.66E-07 | 9.24E-06 |
| GOBP_POSITIVE_REGULATION_OF_CHEMOTAXIS | -0.527832471 | -1.973873923 | 6.75E-07 | 9.34E-06 |
| GOMF_MHC_PROTEIN_BINDING | -0.824710211 | -2.262912241 | 6.90E-07 | 9.52E-06 |
| GOBP_NEGATIVE_REGULATION_OF_WNT_SIGNALING_PATHWAY | 0.504147315 | 1.924501833 | 7.08E-07 | 9.74E-06 |
| GOBP_POSITIVE_REGULATION_OF_IMMUNOGLOBULIN_PRODUCTION | -0.699871903 | -2.270392138 | 7.74E-07 | 1.06E-05 |
| GOBP_POSITIVE_REGULATION_OF_LEUKOCYTE_APOPTOTIC_PROCESS | -0.798582431 | -2.216085337 | 7.86E-07 | 1.08E-05 |
| GOBP_T_CELL_MIGRATION | -0.652445361 | -2.22158759 | 8.43E-07 | 1.15E-05 |
| GOBP_RESPIRATORY_BURST | -0.761305995 | -2.251654577 | 9.42E-07 | 1.28E-05 |
| GOBP_REGULATION_OF_NIK_NF_KAPPAB_SIGNALING | -0.585559493 | -2.099353756 | 9.96E-07 | 1.35E-05 |
| GOBP_REGULATION_OF_CHROMOSOME_ORGANIZATION | -0.455072804 | -1.816050149 | 1.00E-06 | 1.35E-05 |
| GOBP_EMBRYONIC_ORGAN_DEVELOPMENT | 0.392825741 | 1.653185486 | 1.00E-06 | 1.35E-05 |
| GOBP_POSITIVE_REGULATION_OF_PEPTIDASE_ACTIVITY | -0.497469415 | -1.89458673 | 1.05E-06 | 1.41E-05 |
| GOBP_ANTIGEN_PROCESSING_AND_PRESENTATION_VIA_MHC_CLASS_IB | -0.827429532 | -2.226822915 | 1.07E-06 | 1.44E-05 |
| GOBP_EOSINOPHIL_MIGRATION | -0.796887956 | -2.234221268 | 1.14E-06 | 1.52E-05 |
| GOBP_MATURE_B_CELL_DIFFERENTIATION | -0.744304404 | -2.259871889 | 1.18E-06 | 1.58E-05 |
| GOBP_TYPE_2_IMMUNE_RESPONSE | -0.72743334 | -2.259749083 | 1.18E-06 | 1.58E-05 |
| GOBP_CALCIUM_ION_TRANSMEMBRANE_IMPORT_INTO_CYTOSOL | -0.483353258 | -1.869662704 | 1.20E-06 | 1.59E-05 |
| GOBP_REGULATION_OF_DEFENSE_RESPONSE_TO_VIRUS | -0.621634577 | -2.145653937 | 1.24E-06 | 1.64E-05 |
| GOBP_ERK1_AND_ERK2_CASCADE | -0.423900583 | -1.747224172 | 1.24E-06 | 1.64E-05 |
| GOBP_RENAL_SYSTEM_PROCESS | 0.53662529 | 1.960990393 | 1.26E-06 | 1.65E-05 |
| GOBP_NEGATIVE_REGULATION_OF_T_CELL_MEDIATED_IMMUNITY | -0.795867605 | -2.231360528 | 1.28E-06 | 1.69E-05 |
| GOBP_POSITIVE_REGULATION_OF_CYSTEINE_TYPE_ENDOPEPTIDASE_ACTIVITY | -0.523773952 | -1.94739874 | 1.30E-06 | 1.71E-05 |
| GOBP_RESPONSE_TO_PROTOZOAN | -0.816919154 | -2.241534454 | 1.33E-06 | 1.73E-05 |
| GOBP_CYTOKINE_PRODUCTION_INVOLVED_IN_INFLAMMATORY_RESPONSE | -0.665146271 | -2.193990111 | 1.33E-06 | 1.73E-05 |
| GOBP_NEGATIVE_REGULATION_OF_T_CELL_DIFFERENTIATION | -0.688283014 | -2.219159699 | 1.34E-06 | 1.74E-05 |
| GOBP_POSITIVE_REGULATION_OF_RESPONSE_TO_CYTOKINE_STIMULUS | -0.659873367 | -2.203616717 | 1.34E-06 | 1.74E-05 |
| GOBP_POSITIVE_REGULATION_OF_CHEMOKINE_PRODUCTION | -0.631888142 | -2.169018776 | 1.37E-06 | 1.77E-05 |
| GOBP_CD4_POSITIVE_ALPHA_BETA_T_CELL_CYTOKINE_PRODUCTION | -0.849613692 | -2.210374944 | 1.41E-06 | 1.81E-05 |
| GOBP_POSITIVE_REGULATION_OF_INTERLEUKIN_8_PRODUCTION | -0.644549213 | -2.164935067 | 1.41E-06 | 1.81E-05 |
| GOBP_POSITIVE_REGULATION_OF_ERK1_AND_ERK2_CASCADE | -0.476821598 | -1.868409269 | 1.41E-06 | 1.81E-05 |
| GOBP_T_CELL_HOMEOSTASIS | -0.711513832 | -2.254103381 | 1.44E-06 | 1.84E-05 |
| GOBP_NEGATIVE_REGULATION_OF_CANONICAL_WNT_SIGNALING_PATHWAY | 0.526805006 | 1.94695186 | 1.46E-06 | 1.86E-05 |
| GOBP_REGULATION_OF_TYPE_2_IMMUNE_RESPONSE | -0.74802396 | -2.215685574 | 1.46E-06 | 1.86E-05 |
| GOBP_POSITIVE_REGULATION_OF_ANTIGEN_RECEPTOR_MEDIATED_SIGNALING_PATHWAY | -0.812643614 | -2.220636101 | 1.50E-06 | 1.90E-05 |
| GOBP_POSITIVE_REGULATION_OF_LEUKOCYTE_ADHESION_TO_VASCULAR_ENDOTHELIAL_CELL | -0.812625031 | -2.220585321 | 1.50E-06 | 1.90E-05 |
| GOBP_CYTOSOLIC_PATTERN_RECOGNITION_RECEPTOR_SIGNALING_PATHWAY | -0.59824928 | -2.110860315 | 1.54E-06 | 1.95E-05 |
| GOBP_T_CELL_LINEAGE_COMMITMENT | -0.763851792 | -2.218923406 | 1.62E-06 | 2.03E-05 |
| GOBP_REGULATION_OF_PATTERN_RECOGNITION_RECEPTOR_SIGNALING_PATHWAY | -0.574374291 | -2.047484468 | 1.62E-06 | 2.03E-05 |
| GOBP_SISTER_CHROMATID_SEGREGATION | -0.445230563 | -1.774481872 | 1.62E-06 | 2.03E-05 |
| GOBP_SUPEROXIDE_ANION_GENERATION | -0.713374707 | -2.22191683 | 1.63E-06 | 2.03E-05 |
| GOBP_NEGATIVE_REGULATION_OF_HEMOPOIESIS | -0.55200253 | -1.9927447 | 1.63E-06 | 2.03E-05 |
| GOBP_POSITIVE_REGULATION_OF_PROTEOLYSIS | -0.403566927 | -1.68311146 | 1.67E-06 | 2.08E-05 |
| GOBP_REGULATION_OF_VIRAL_GENOME_REPLICATION | -0.61544152 | -2.15903556 | 1.70E-06 | 2.10E-05 |
| GOBP_GLOMERULUS_DEVELOPMENT | 0.613824823 | 2.070068233 | 1.72E-06 | 2.12E-05 |
| GOBP_T_CELL_ACTIVATION_VIA_T_CELL_RECEPTOR_CONTACT_WITH_ANTIGEN_BOUND_TO_MHC_MOLECULE_ON_ANTIGEN_PRESENTING_CELL | -0.933423045 | -2.076614152 | 1.72E-06 | 2.13E-05 |
| GOBP_FC_GAMMA_RECEPTOR_SIGNALING_PATHWAY | -0.758336454 | -2.232545107 | 1.83E-06 | 2.25E-05 |
| GOBP_POSITIVE_REGULATION_OF_T_CELL_CYTOKINE_PRODUCTION | -0.789374427 | -2.213155715 | 1.95E-06 | 2.39E-05 |
| GOBP_MORPHOGENESIS_OF_EMBRYONIC_EPITHELIUM | 0.499228937 | 1.892233876 | 1.95E-06 | 2.39E-05 |
| GOBP_REGULATION_OF_NEUTROPHIL_MIGRATION | -0.707156659 | -2.240299688 | 1.96E-06 | 2.39E-05 |
| GOBP_NEGATIVE_REGULATION_OF_LEUKOCYTE_APOPTOTIC_PROCESS | -0.664020814 | -2.193261199 | 1.99E-06 | 2.42E-05 |
| GOBP_EXOCYTOSIS | -0.412345586 | -1.715266481 | 2.07E-06 | 2.52E-05 |
| GOBP_CARDIAC_MUSCLE_CELL_ACTION_POTENTIAL | 0.600083413 | 2.04875428 | 2.08E-06 | 2.53E-05 |
| GOBP_SENSORY_ORGAN_MORPHOGENESIS | 0.442648955 | 1.781111917 | 2.15E-06 | 2.60E-05 |
| GOCC_FICOLIN_1_RICH_GRANULE_LUMEN | -0.54096116 | -1.98108897 | 2.21E-06 | 2.66E-05 |
| GOBP_VACUOLAR_LOCALIZATION | -0.594212782 | -2.095814638 | 2.24E-06 | 2.70E-05 |
| GOBP_T_HELPER_CELL_LINEAGE_COMMITMENT | -0.843575454 | -2.169346502 | 2.26E-06 | 2.71E-05 |
| GOBP_POSITIVE_REGULATION_OF_INTERLEUKIN_2_PRODUCTION | -0.742580772 | -2.199562571 | 2.31E-06 | 2.77E-05 |
| GOBP_REGULATION_OF_CYSTEINE_TYPE_ENDOPEPTIDASE_ACTIVITY | -0.45608997 | -1.807794065 | 2.32E-06 | 2.77E-05 |
| GOBP_RENAL_TUBULE_DEVELOPMENT | 0.553816166 | 1.973346734 | 2.38E-06 | 2.83E-05 |
| GOBP_METANEPHROS_DEVELOPMENT | 0.571409048 | 1.990857848 | 2.39E-06 | 2.84E-05 |
| GOMF_ENDOPEPTIDASE_ACTIVITY | -0.393123715 | -1.662764055 | 2.42E-06 | 2.87E-05 |
| GOBP_MUSCLE_CONTRACTION | 0.407792915 | 1.680500201 | 2.57E-06 | 3.04E-05 |
| GOBP_TYPE_II_INTERFERON_MEDIATED_SIGNALING_PATHWAY | -0.809222459 | -2.220415588 | 2.67E-06 | 3.15E-05 |
| GOBP_DETECTION_OF_EXTERNAL_BIOTIC_STIMULUS | -0.82100493 | -2.160344043 | 2.77E-06 | 3.25E-05 |
| GOBP_POSITIVE_REGULATION_OF_T_HELPER_1_TYPE_IMMUNE_RESPONSE | -0.840772869 | -2.162139348 | 2.78E-06 | 3.26E-05 |
| GOBP_POSITIVE_REGULATION_OF_INFLAMMATORY_RESPONSE_TO_ANTIGENIC_STIMULUS | -0.911829819 | -2.072992447 | 2.78E-06 | 3.26E-05 |
| GOBP_IMMUNOLOGICAL_SYNAPSE_FORMATION | -0.861815617 | -2.130604356 | 2.82E-06 | 3.29E-05 |
| GOBP_CELLULAR_COMPONENT_ASSEMBLY_INVOLVED_IN_MORPHOGENESIS | 0.523189237 | 1.902706844 | 2.91E-06 | 3.40E-05 |
| GOBP_NEGATIVE_T_CELL_SELECTION | -0.908599759 | -2.101612598 | 2.95E-06 | 3.43E-05 |
| GOBP_MUSCLE_ORGAN_DEVELOPMENT | 0.405707992 | 1.671908307 | 3.09E-06 | 3.58E-05 |
| GOBP_POSITIVE_REGULATION_OF_PROTEIN_CONTAINING_COMPLEX_ASSEMBLY | -0.472229335 | -1.826634166 | 3.18E-06 | 3.68E-05 |
| GOBP_AXON_DEVELOPMENT | 0.38053687 | 1.606127831 | 3.18E-06 | 3.68E-05 |
| GOBP_INFLAMMATORY_CELL_APOPTOTIC_PROCESS | -0.820489319 | -2.140787985 | 3.22E-06 | 3.70E-05 |
| GOBP_FAT_CELL_DIFFERENTIATION | 0.459154985 | 1.816349766 | 3.21E-06 | 3.70E-05 |
| GOBP_DIGESTIVE_SYSTEM_DEVELOPMENT | 0.520050711 | 1.936308163 | 3.24E-06 | 3.72E-05 |
| GOBP_EPIDERMIS_DEVELOPMENT | 0.40885487 | 1.682601881 | 3.28E-06 | 3.75E-05 |
| GOBP_HOMOPHILIC_CELL_ADHESION_VIA_PLASMA_MEMBRANE_ADHESION_MOLECULES | 0.508995698 | 1.909600293 | 3.30E-06 | 3.76E-05 |
| GOBP_POSITIVE_REGULATION_OF_HUMORAL_IMMUNE_RESPONSE | -0.839161327 | -2.183181825 | 3.37E-06 | 3.84E-05 |
| GOMF_TRANSMEMBRANE_RECEPTOR_PROTEIN_KINASE_ACTIVITY | 0.590442212 | 2.014653928 | 3.46E-06 | 3.93E-05 |
| GOBP_CELL_CELL_JUNCTION_ORGANIZATION | 0.460154635 | 1.809616404 | 3.54E-06 | 4.01E-05 |
| GOBP_REGULATION_OF_MAST_CELL_ACTIVATION | -0.700529999 | -2.192882539 | 3.60E-06 | 4.06E-05 |
| GOBP_CARDIAC_CHAMBER_MORPHOGENESIS | 0.524815907 | 1.927325077 | 3.61E-06 | 4.07E-05 |
| GOBP_POSITIVE_REGULATION_OF_T_CELL_DIFFERENTIATION_IN_THYMUS | -0.908514817 | -2.065455982 | 3.67E-06 | 4.12E-05 |
| GOBP_NEGATIVE_REGULATION_OF_ALPHA_BETA_T_CELL_DIFFERENTIATION | -0.776533987 | -2.154900376 | 3.73E-06 | 4.19E-05 |
| GOBP_REGULATION_OF_B_CELL_RECEPTOR_SIGNALING_PATHWAY | -0.816159936 | -2.147595213 | 3.95E-06 | 4.42E-05 |
| GOBP_FC_RECEPTOR_MEDIATED_STIMULATORY_SIGNALING_PATHWAY | -0.74634187 | -2.19723301 | 3.96E-06 | 4.42E-05 |
| GOBP_NEGATIVE_REGULATION_OF_INTERLEUKIN_12_PRODUCTION | -0.834311387 | -2.145522941 | 4.24E-06 | 4.72E-05 |
| GOBP_MACROPHAGE_ACTIVATION_INVOLVED_IN_IMMUNE_RESPONSE | -0.833493836 | -2.143420519 | 4.54E-06 | 5.05E-05 |
| GOBP_REGULATION_OF_DNA_RECOMBINATION | -0.538397026 | -1.948531881 | 4.74E-06 | 5.26E-05 |
| GOCC_FICOLIN_1_RICH_GRANULE_MEMBRANE | -0.643753637 | -2.149785621 | 4.83E-06 | 5.33E-05 |
| GOCC_PHAGOCYTIC_CUP | -0.774225404 | -2.148494006 | 4.82E-06 | 5.33E-05 |
| GOMF_T_CELL_RECEPTOR_BINDING | -0.924596389 | -2.056977228 | 4.85E-06 | 5.35E-05 |
| GOBP_PYROPTOSIS | -0.773400414 | -2.146204638 | 4.97E-06 | 5.46E-05 |
| GOCC_MEMBRANE_MICRODOMAIN | -0.40776468 | -1.681540225 | 4.99E-06 | 5.48E-05 |
| GOBP_BRANCH_ELONGATION_OF_AN_EPITHELIUM | 0.822644996 | 2.157463296 | 5.10E-06 | 5.59E-05 |
| GOBP_REGULATION_OF_CD8_POSITIVE_ALPHA_BETA_T_CELL_ACTIVATION | -0.832306768 | -2.140367842 | 5.21E-06 | 5.68E-05 |
| GOBP_REGULATION_OF_CHRONIC_INFLAMMATORY_RESPONSE | -0.924158025 | -2.056001986 | 5.22E-06 | 5.68E-05 |
| GOBP_POSITIVE_REGULATION_OF_DEFENSE_RESPONSE_TO_VIRUS_BY_HOST | -0.772252781 | -2.143019929 | 5.56E-06 | 6.05E-05 |
| GOBP_REGULATION_OF_TOLERANCE_INDUCTION | -0.833063612 | -2.167317868 | 5.71E-06 | 6.20E-05 |
| GOBP_SENSORY_SYSTEM_DEVELOPMENT | 0.391043571 | 1.633843122 | 5.76E-06 | 6.23E-05 |
| GOBP_ACUTE_INFLAMMATORY_RESPONSE | -0.540974172 | -1.948759684 | 5.90E-06 | 6.37E-05 |
| GOBP_DENDRITIC_CELL_ANTIGEN_PROCESSING_AND_PRESENTATION | -0.901208705 | -2.084516915 | 5.94E-06 | 6.40E-05 |
| GOBP_VIRAL_GENOME_REPLICATION | -0.536664954 | -1.950078157 | 6.01E-06 | 6.47E-05 |
| GOBP_INTERFERON_ALPHA_PRODUCTION | -0.756642057 | -2.16724679 | 6.22E-06 | 6.68E-05 |
| GOBP_T_HELPER_17_CELL_DIFFERENTIATION | -0.75635875 | -2.166435317 | 6.45E-06 | 6.87E-05 |
| GOBP_CELL_SUBSTRATE_JUNCTION_ORGANIZATION | 0.537962784 | 1.928768871 | 6.45E-06 | 6.87E-05 |
| GOBP_MUSCLE_SYSTEM_PROCESS | 0.384984337 | 1.615574776 | 6.44E-06 | 6.87E-05 |
| GOBP_CARDIAC_CHAMBER_DEVELOPMENT | 0.483188114 | 1.847739688 | 6.67E-06 | 7.10E-05 |
| GOBP_MESENCHYME_DEVELOPMENT | 0.413309261 | 1.682536994 | 6.96E-06 | 7.38E-05 |
| GOBP_RESPONSE_TO_INTERFERON_BETA | -0.772341261 | -2.185375609 | 6.98E-06 | 7.40E-05 |
| GOBP_REGULATION_OF_T_HELPER_17_TYPE_IMMUNE_RESPONSE | -0.772088163 | -2.184659457 | 7.13E-06 | 7.54E-05 |
| GOBP_OUTFLOW_TRACT_MORPHOGENESIS | 0.57330832 | 1.958637718 | 7.23E-06 | 7.62E-05 |
| GOBP_POSITIVE_REGULATION_OF_CYTOKINE_PRODUCTION_INVOLVED_IN_INFLAMMATORY_RESPONSE | -0.803348818 | -2.1620156 | 7.33E-06 | 7.71E-05 |
| GOBP_COLLAGEN_METABOLIC_PROCESS | -0.564545915 | -2.008385542 | 7.44E-06 | 7.81E-05 |
| GOMF_CARBOHYDRATE_BINDING | -0.439888933 | -1.75440487 | 7.50E-06 | 7.86E-05 |
| GOBP_T_CELL_APOPTOTIC_PROCESS | -0.64827688 | -2.141258977 | 7.66E-06 | 8.00E-05 |
| GOBP_FC_RECEPTOR_SIGNALING_PATHWAY | -0.646705026 | -2.134842576 | 7.80E-06 | 8.13E-05 |
| GOBP_DETECTION_OF_CHEMICAL_STIMULUS_INVOLVED_IN_SENSORY_PERCEPTION_OF_TASTE | 0.689585883 | 2.063847069 | 7.88E-06 | 8.21E-05 |
| GOBP_REGULATION_OF_CELL_JUNCTION_ASSEMBLY | 0.455633145 | 1.78889756 | 7.91E-06 | 8.22E-05 |
| GOMF_STRUCTURAL_CONSTITUENT_OF_MUSCLE | 0.671980994 | 2.052459744 | 7.96E-06 | 8.24E-05 |
| GOBP_LIPID_OXIDATION | 0.529516311 | 1.921679187 | 7.96E-06 | 8.24E-05 |
| GOBP_CD4_POSITIVE_OR_CD8_POSITIVE_ALPHA_BETA_T_CELL_LINEAGE_COMMITMENT | -0.796807315 | -2.186349825 | 8.08E-06 | 8.34E-05 |
| GOBP_REGULATION_OF_MICROGLIAL_CELL_ACTIVATION | -0.850162262 | -2.101794609 | 8.26E-06 | 8.51E-05 |
| GOBP_NEGATIVE_REGULATION_OF_CD4_POSITIVE_ALPHA_BETA_T_CELL_DIFFERENTIATION | -0.809437682 | -2.111952494 | 8.53E-06 | 8.75E-05 |
| GOBP_EMBRYONIC_ORGAN_MORPHOGENESIS | 0.42022666 | 1.695986174 | 8.52E-06 | 8.75E-05 |
| GOBP_ANTIMICROBIAL_HUMORAL_RESPONSE | -0.534236206 | -1.924487405 | 8.61E-06 | 8.81E-05 |
| GOBP_ACTION_POTENTIAL | 0.502521388 | 1.865374864 | 8.63E-06 | 8.82E-05 |
| GOBP_SOMATIC_DIVERSIFICATION_OF_IMMUNE_RECEPTORS | -0.606341356 | -2.106717876 | 9.00E-06 | 9.17E-05 |
| GOBP_SENSORY_PERCEPTION_OF_BITTER_TASTE | 0.700889013 | 2.104270237 | 9.08E-06 | 9.24E-05 |
| GOBP_ACTIN_MEDIATED_CELL_CONTRACTION | 0.545663226 | 1.932418642 | 9.11E-06 | 9.24E-05 |
| GOMF_TUMOR_NECROSIS_FACTOR_RECEPTOR_SUPERFAMILY_BINDING | -0.68849454 | -2.181177373 | 9.27E-06 | 9.39E-05 |
| GOBP_OSTEOCLAST_DIFFERENTIATION | -0.546161605 | -1.944853661 | 9.43E-06 | 9.53E-05 |
| GOBP_RESPONSE_TO_FUNGUS | -0.642348816 | -2.121678575 | 9.57E-06 | 9.65E-05 |
| GOBP_REGULATION_OF_NATURAL_KILLER_CELL_ACTIVATION | -0.701686336 | -2.179766814 | 9.62E-06 | 9.68E-05 |
| GOMF_CELL_CELL_ADHESION_MEDIATOR_ACTIVITY | 0.646427984 | 2.054315418 | 9.97E-06 | 9.99E-05 |
| GOBP_REGULATION_OF_CHROMOSOME_SEGREGATION | -0.518856874 | -1.921009457 | 9.96E-06 | 9.99E-05 |
| GOBP_FOCAL_ADHESION_ASSEMBLY | 0.561064251 | 1.941226863 | 1.00E-05 | 0.000100502 |
| GOBP_CD8_POSITIVE_ALPHA_BETA_T_CELL_DIFFERENTIATION | -0.881385454 | -2.0818801 | 1.03E-05 | 0.000103246 |
| GOMF_SIGNALING_RECEPTOR_REGULATOR_ACTIVITY | -0.366374991 | -1.578308141 | 1.04E-05 | 0.000103718 |
| GOBP_DETECTION_OF_OTHER_ORGANISM | -0.844192701 | -2.139001473 | 1.06E-05 | 0.00010533 |
| GOBP_INTRINSIC_APOPTOTIC_SIGNALING_PATHWAY | -0.411944891 | -1.683215409 | 1.18E-05 | 0.000117199 |
| GOBP_LUNG_CELL_DIFFERENTIATION | 0.741212426 | 2.087275061 | 1.20E-05 | 0.000118416 |
| GOBP_POSITIVE_REGULATION_OF_CALCIUM_ION_TRANSPORT | -0.511438724 | -1.873710056 | 1.22E-05 | 0.000120183 |
| GOBP_HEART_PROCESS | 0.4308907 | 1.718550641 | 1.26E-05 | 0.000124704 |
| GOBP_MORPHOGENESIS_OF_AN_EPITHELIUM | 0.361974409 | 1.543269577 | 1.31E-05 | 0.000128551 |
| GOBP_REGULATION_OF_MYELOID_CELL_DIFFERENTIATION | -0.454799508 | -1.764534576 | 1.31E-05 | 0.000128719 |
| GOBP_POSITIVE_REGULATION_OF_NEUTROPHIL_MIGRATION | -0.721283814 | -2.133284136 | 1.33E-05 | 0.000130656 |
| GOMF_NON_MEMBRANE_SPANNING_PROTEIN_TYROSINE_KINASE_ACTIVITY | -0.682403738 | -2.136141555 | 1.35E-05 | 0.000131663 |
| GOBP_REGULATION_OF_VENTRICULAR_CARDIAC_MUSCLE_CELL_ACTION_POTENTIAL | 0.875776451 | 2.030372396 | 1.38E-05 | 0.000134273 |
| GOBP_RESPONSE_TO_TUMOR_CELL | -0.69301797 | -2.152838806 | 1.39E-05 | 0.000135857 |
| GOBP_CARDIAC_MUSCLE_CELL_PROLIFERATION | 0.646435415 | 2.043281734 | 1.41E-05 | 0.000136742 |
| GOMF_PHOSPHOTYROSINE_RESIDUE_BINDING | -0.645759433 | -2.096715535 | 1.42E-05 | 0.000137653 |
| GOBP_MORPHOGENESIS_OF_A_POLARIZED_EPITHELIUM | 0.533851814 | 1.878475566 | 1.43E-05 | 0.000138852 |
| GOBP_RENAL_ABSORPTION | 0.721649453 | 2.054686987 | 1.44E-05 | 0.000138938 |
| GOBP_STRIATED_MUSCLE_CELL_PROLIFERATION | 0.579783148 | 1.962601552 | 1.51E-05 | 0.000145886 |
| GOBP_POSITIVE_REGULATION_OF_REGULATORY_T_CELL_DIFFERENTIATION | -0.798590185 | -2.101363204 | 1.52E-05 | 0.00014615 |
| GOCC_AZUROPHIL_GRANULE_LUMEN | -0.567529617 | -2.00170194 | 1.53E-05 | 0.000147413 |
| GOCC_CLATHRIN_COATED_VESICLE_MEMBRANE | -0.511128011 | -1.884440775 | 1.55E-05 | 0.000148997 |
| GOBP_INTERMEDIATE_FILAMENT_BASED_PROCESS | 0.554564008 | 1.918736665 | 1.56E-05 | 0.00014975 |
| GOBP_POSITIVE_REGULATION_OF_ANIMAL_ORGAN_MORPHOGENESIS | 0.709544153 | 2.079240028 | 1.61E-05 | 0.000154031 |
| GOBP_EAR_MORPHOGENESIS | 0.504169361 | 1.830190825 | 1.62E-05 | 0.000154031 |
| GOBP_CAMERA_TYPE_EYE_MORPHOGENESIS | 0.500523486 | 1.842588358 | 1.62E-05 | 0.000154592 |
| GOBP_REGULATION_OF_MYELOID_LEUKOCYTE_DIFFERENTIATION | -0.523097349 | -1.900777585 | 1.67E-05 | 0.000158694 |
| GOBP_APPENDAGE_DEVELOPMENT | 0.455725832 | 1.759739034 | 1.67E-05 | 0.000158694 |
| GOBP_TUMOR_NECROSIS_FACTOR_MEDIATED_SIGNALING_PATHWAY | -0.538746801 | -1.925256748 | 1.72E-05 | 0.000162712 |
| GOBP_MONOCARBOXYLIC_ACID_CATABOLIC_PROCESS | 0.498923199 | 1.836697187 | 1.75E-05 | 0.000165665 |
| GOBP_REGULATION_OF_REGULATED_SECRETORY_PATHWAY | -0.503236077 | -1.85637388 | 1.76E-05 | 0.000166259 |
| GOBP_NEGATIVE_REGULATION_OF_NF_KAPPAB_TRANSCRIPTION_FACTOR_ACTIVITY | -0.568026405 | -2.006847676 | 1.77E-05 | 0.000166513 |
| GOBP_ESTABLISHMENT_OF_TISSUE_POLARITY | 0.575752773 | 1.969546087 | 1.77E-05 | 0.000166513 |
| GOBP_REGULATION_OF_LYMPHOCYTE_MIGRATION | -0.605772086 | -2.04752301 | 1.79E-05 | 0.000167892 |
| GOBP_SUPEROXIDE_METABOLIC_PROCESS | -0.602947548 | -2.062903132 | 1.81E-05 | 0.000168945 |
| GOBP_CHEMOKINE_PRODUCTION | -0.546961857 | -1.958435127 | 1.82E-05 | 0.000170159 |
| GOBP_REGULATION_OF_DENDRITIC_CELL_DIFFERENTIATION | -0.8729115 | -2.061864163 | 1.90E-05 | 0.000176909 |
| GOCC_AZUROPHIL_GRANULE | -0.493087547 | -1.846945763 | 1.94E-05 | 0.000180195 |
| GOBP_REGULATION_OF_CARDIAC_MUSCLE_CELL_ACTION_POTENTIAL | 0.714747515 | 2.035035724 | 1.96E-05 | 0.000181444 |
| GOBP_COLLAGEN_CATABOLIC_PROCESS | -0.6752917 | -2.103301364 | 2.02E-05 | 0.000186671 |
| GOBP_T_CELL_TOLERANCE_INDUCTION | -0.848283211 | -2.081855596 | 2.02E-05 | 0.000186671 |
| GOBP_REGULATION_OF_B_CELL_DIFFERENTIATION | -0.710906994 | -2.124879781 | 2.03E-05 | 0.000187021 |
| GOCC_NADPH_OXIDASE_COMPLEX | -0.837713911 | -2.071019454 | 2.11E-05 | 0.000194183 |
| GOBP_POSITIVE_REGULATION_OF_GTPASE_ACTIVITY | -0.425256952 | -1.708258434 | 2.14E-05 | 0.000196989 |
| GOMF_CYTOKINE_BINDING | -0.49277889 | -1.830765117 | 2.16E-05 | 0.000198087 |
| GOBP_RESPONSE_TO_BMP | 0.463730759 | 1.769570323 | 2.21E-05 | 0.00020202 |
| GOBP_REGULATION_OF_APOPTOTIC_SIGNALING_PATHWAY | -0.39054867 | -1.635965114 | 2.23E-05 | 0.000203443 |
| GOBP_PEPTIDYL_TYROSINE_MODIFICATION | -0.385233168 | -1.614753524 | 2.29E-05 | 0.000208626 |
| GOBP_REGULATION_OF_ESTABLISHMENT_OF_PLANAR_POLARITY | 0.614299706 | 2.004027234 | 2.37E-05 | 0.000215733 |
| GOBP_PLATELET_ACTIVATION | -0.496576094 | -1.841823939 | 2.41E-05 | 0.000219415 |
| GOBP_FATTY_ACID_METABOLIC_PROCESS | 0.385468609 | 1.593735403 | 2.54E-05 | 0.000230128 |
| GOBP_FATTY_ACID_BETA_OXIDATION | 0.576949442 | 1.964460132 | 2.56E-05 | 0.000231673 |
| GOBP_CARDIAC_CONDUCTION | 0.535878373 | 1.869243505 | 2.58E-05 | 0.000232917 |
| GOMF_SERINE_HYDROLASE_ACTIVITY | -0.459475757 | -1.767998189 | 2.58E-05 | 0.000233013 |
| GOBP_MYD88_DEPENDENT_TOLL_LIKE_RECEPTOR_SIGNALING_PATHWAY | -0.816247898 | -2.12356971 | 2.61E-05 | 0.000235256 |
| GOBP_T_HELPER_1_CELL_DIFFERENTIATION | -0.790506139 | -2.08009132 | 2.62E-05 | 0.000235256 |
| GOBP_CELL_CELL_JUNCTION_ASSEMBLY | 0.470915064 | 1.780915869 | 2.72E-05 | 0.00024439 |
| GOBP_CALCIUM_ION_HOMEOSTASIS | -0.397872053 | -1.640535226 | 2.74E-05 | 0.000245526 |
| GOBP_POSITIVE_REGULATION_OF_LYMPHOCYTE_MIGRATION | -0.685150915 | -2.125587341 | 2.79E-05 | 0.000249408 |
| GOBP_ACTIN_FILAMENT_BASED_MOVEMENT | 0.494418013 | 1.820112142 | 2.80E-05 | 0.000249408 |
| GOBP_NEGATIVE_REGULATION_OF_B_CELL_PROLIFERATION | -0.833302756 | -2.060114075 | 2.86E-05 | 0.000254385 |
| GOBP_NEGATIVE_REGULATION_OF_REGULATED_SECRETORY_PATHWAY | -0.774097223 | -2.124035883 | 2.87E-05 | 0.000255102 |
| GOBP_POSITIVE_REGULATION_OF_T_CELL_RECEPTOR_SIGNALING_PATHWAY | -0.843500241 | -2.070117236 | 2.92E-05 | 0.000258766 |
| GOBP_RESPONSE_TO_FATTY_ACID | 0.599700387 | 1.972929612 | 2.93E-05 | 0.000259319 |
| GOMF_G_PROTEIN_COUPLED_RECEPTOR_BINDING | -0.420107628 | -1.692338587 | 2.97E-05 | 0.000262551 |
| GOBP_REGULATION_OF_PEPTIDYL_TYROSINE_PHOSPHORYLATION | -0.419048542 | -1.671387194 | 3.03E-05 | 0.000267237 |
| GOBP_ANTIMICROBIAL_HUMORAL_IMMUNE_RESPONSE_MEDIATED_BY_ANTIMICROBIAL_PEPTIDE | -0.586948642 | -2.039338376 | 3.04E-05 | 0.000268117 |
| GOBP_POSITIVE_REGULATION_OF_PEPTIDYL_TYROSINE_PHOSPHORYLATION | -0.460387631 | -1.757127548 | 3.05E-05 | 0.000268623 |
| GOBP_STRIATED_MUSCLE_CELL_DEVELOPMENT | 0.586363374 | 1.977933838 | 3.20E-05 | 0.000280613 |
| GOBP_NUCLEOTIDE_BINDING_DOMAIN_LEUCINE_RICH_REPEAT_CONTAINING_RECEPTOR_SIGNALING_PATHWAY | -0.781438438 | -2.103049206 | 3.20E-05 | 0.000280751 |
| GOBP_IMMUNE_RESPONSE_TO_TUMOR_CELL | -0.771739695 | -2.108861741 | 3.30E-05 | 0.000288404 |
| GOBP_NON_CANONICAL_WNT_SIGNALING_PATHWAY | 0.571545578 | 1.946060467 | 3.35E-05 | 0.000292391 |
| GOBP_POSITIVE_REGULATION_OF_NIK_NF_KAPPAB_SIGNALING | -0.597318275 | -2.033878921 | 3.39E-05 | 0.000295036 |
| GOBP_MESONEPHRIC_TUBULE_MORPHOGENESIS | 0.566246286 | 1.916778443 | 3.46E-05 | 0.000300574 |
| GOMF_CXCR_CHEMOKINE_RECEPTOR_BINDING | -0.825201855 | -2.090882783 | 3.50E-05 | 0.000304008 |
| GOBP_EPITHELIAL_TUBE_FORMATION | 0.484888716 | 1.810521101 | 3.51E-05 | 0.000304008 |
| GOBP_DOUBLE_STRAND_BREAK_REPAIR | -0.397944317 | -1.621565508 | 3.55E-05 | 0.000306858 |
| GOBP_PROTEIN_LOCALIZATION_TO_CHROMOSOME | -0.550516886 | -1.94088969 | 3.57E-05 | 0.00030807 |
| GOMF_CELL_ADHESION_MEDIATOR_ACTIVITY | 0.59725695 | 1.989828055 | 3.77E-05 | 0.0003247 |
| GOBP_SEX_DIFFERENTIATION | 0.408009153 | 1.647669531 | 3.78E-05 | 0.000325665 |
| GOBP_EXTRACELLULAR_MATRIX_DISASSEMBLY | -0.603869403 | -2.018473913 | 3.79E-05 | 0.000325932 |
| GOMF_TRANSMEMBRANE_RECEPTOR_PROTEIN_TYROSINE_KINASE_ACTIVITY | 0.589298809 | 1.968662877 | 3.80E-05 | 0.000325932 |
| GOBP_POSITIVE_REGULATION_OF_MYELOID_LEUKOCYTE_MEDIATED_IMMUNITY | -0.782850273 | -2.042581689 | 3.82E-05 | 0.000327078 |
| GOBP_CARDIAC_MUSCLE_CELL_CONTRACTION | 0.561954337 | 1.906488597 | 3.85E-05 | 0.000328664 |
| GOBP_OTIC_VESICLE_DEVELOPMENT | 0.824312763 | 2.041688904 | 3.88E-05 | 0.000330861 |
| GOBP_POSITIVE_REGULATION_OF_INTERFERON_BETA_PRODUCTION | -0.687560189 | -2.068352975 | 3.93E-05 | 0.000334626 |
| GOBP_NEGATIVE_REGULATION_OF_CHROMOSOME_ORGANIZATION | -0.534806161 | -1.88940367 | 3.97E-05 | 0.000337719 |
| GOBP_CHROMOSOME_SEPARATION | -0.548280389 | -1.923778481 | 3.98E-05 | 0.000337858 |
| GOBP_ORGAN_GROWTH | 0.460103834 | 1.75931052 | 4.02E-05 | 0.000340493 |
| GOMF_WNT_RECEPTOR_ACTIVITY | 0.823864039 | 2.040577486 | 4.14E-05 | 0.000350001 |
| GOBP_NEGATIVE_REGULATION_OF_ALPHA_BETA_T_CELL_ACTIVATION | -0.677182291 | -2.100865768 | 4.18E-05 | 0.000353258 |
| GOMF_WNT_PROTEIN_BINDING | 0.708500187 | 2.037423371 | 4.21E-05 | 0.000354735 |
| GOMF_PROTEIN_PHOSPHORYLATED_AMINO_ACID_BINDING | -0.601769258 | -2.011454037 | 4.23E-05 | 0.000355643 |
| GOBP_LUNG_EPITHELIUM_DEVELOPMENT | 0.647711339 | 1.978331919 | 4.25E-05 | 0.000355947 |
| GOBP_HEART_GROWTH | 0.553851526 | 1.896357319 | 4.24E-05 | 0.000355947 |
| GOCC_CHROMOSOME_CENTROMERIC_REGION | -0.428194966 | -1.697037604 | 4.25E-05 | 0.000355947 |
| GOBP_NEGATIVE_REGULATION_OF_TYPE_I_INTERFERON_MEDIATED_SIGNALING_PATHWAY | -0.80046065 | -2.05847207 | 4.29E-05 | 0.000358763 |
| GOBP_EAR_DEVELOPMENT | 0.432775752 | 1.701569618 | 4.53E-05 | 0.000377833 |
| GOBP_REGULATION_OF_NEUTROPHIL_ACTIVATION | -0.861801277 | -2.035621216 | 4.54E-05 | 0.000377953 |
| GOBP_POSITIVE_REGULATION_OF_TOLERANCE_INDUCTION | -0.876963442 | -2.028437053 | 4.64E-05 | 0.000385622 |
| GOBP_POSITIVE_REGULATION_OF_NATURAL_KILLER_CELL_MEDIATED_IMMUNITY | -0.745650779 | -2.109853645 | 4.70E-05 | 0.000389998 |
| GOBP_GLANDULAR_EPITHELIAL_CELL_DIFFERENTIATION | 0.580036557 | 1.956592081 | 4.83E-05 | 0.000400577 |
| GOBP_POSITIVE_REGULATION_OF_DNA_BINDING_TRANSCRIPTION_FACTOR_ACTIVITY | -0.410622444 | -1.656687711 | 4.88E-05 | 0.000403961 |
| GOCC_COATED_VESICLE_MEMBRANE | -0.436830969 | -1.694820102 | 4.99E-05 | 0.000412153 |
| GOBP_RESPONSE_TO_INTERLEUKIN_4 | -0.695819904 | -2.079784921 | 5.03E-05 | 0.000414257 |
| GOBP_POSITIVE_REGULATION_OF_T_CELL_MIGRATION | -0.700793018 | -2.072680126 | 5.09E-05 | 0.000418215 |
| GOBP_POSITIVE_REGULATION_OF_CYTOSOLIC_CALCIUM_ION_CONCENTRATION | -0.454460032 | -1.734504119 | 5.09E-05 | 0.000418215 |
| GOBP_REGULATION_OF_GRANULOCYTE_CHEMOTAXIS | -0.61950229 | -2.045043432 | 5.13E-05 | 0.000420622 |
| GOBP_REGULATION_OF_T_CELL_APOPTOTIC_PROCESS | -0.67720041 | -2.076539322 | 5.26E-05 | 0.000430662 |
| GOBP_RELEASE_OF_CYTOCHROME_C_FROM_MITOCHONDRIA | -0.604052718 | -2.001613614 | 5.31E-05 | 0.00043367 |
| GOBP_POSITIVE_REGULATION_OF_T_HELPER_CELL_DIFFERENTIATION | -0.778530317 | -2.048578847 | 5.63E-05 | 0.000459755 |
| GOBP_CARDIAC_VENTRICLE_DEVELOPMENT | 0.485529881 | 1.782815929 | 5.74E-05 | 0.000467212 |
| GOBP_ANTERIOR_POSTERIOR_PATTERN_SPECIFICATION | 0.430711234 | 1.688791692 | 5.74E-05 | 0.000467212 |

**Table 6b. KEGG of GSEA analysis**.

| ID | enrichmentScore | NES | pvalue | qvalue |
| --- | --- | --- | --- | --- |
| KEGG_AUTOIMMUNE_THYROID_DISEASE | -0.876638967 | -2.715915597 | 1.00E-10 | 7.43E-10 |
| KEGG_ANTIGEN_PROCESSING_AND_PRESENTATION | -0.799834833 | -2.708609203 | 1.00E-10 | 7.43E-10 |
| KEGG_LEISHMANIA_INFECTION | -0.804899995 | -2.69870256 | 1.00E-10 | 7.43E-10 |
| KEGG_ALLOGRAFT_REJECTION | -0.921298492 | -2.692376486 | 1.00E-10 | 7.43E-10 |
| KEGG_GRAFT_VERSUS_HOST_DISEASE | -0.909739604 | -2.688564825 | 1.00E-10 | 7.43E-10 |
| KEGG_PRIMARY_IMMUNODEFICIENCY | -0.892174583 | -2.636654699 | 1.00E-10 | 7.43E-10 |
| KEGG_TYPE_I_DIABETES_MELLITUS | -0.872008874 | -2.613690252 | 1.00E-10 | 7.43E-10 |
| KEGG_SYSTEMIC_LUPUS_ERYTHEMATOSUS | -0.820009606 | -2.601242741 | 1.00E-10 | 7.43E-10 |
| KEGG_NATURAL_KILLER_CELL_MEDIATED_CYTOTOXICITY | -0.702906355 | -2.592166403 | 1.00E-10 | 7.43E-10 |
| KEGG_TOLL_LIKE_RECEPTOR_SIGNALING_PATHWAY | -0.735965063 | -2.567292737 | 1.00E-10 | 7.43E-10 |
| KEGG_CHEMOKINE_SIGNALING_PATHWAY | -0.646681912 | -2.473912193 | 1.00E-10 | 7.43E-10 |
| KEGG_INTESTINAL_IMMUNE_NETWORK_FOR_IGA_PRODUCTION | -0.793054895 | -2.439609404 | 1.00E-10 | 7.43E-10 |
| KEGG_ASTHMA | -0.877038659 | -2.430725212 | 1.00E-10 | 7.43E-10 |
| KEGG_CYTOKINE_CYTOKINE_RECEPTOR_INTERACTION | -0.605968702 | -2.42352993 | 1.00E-10 | 7.43E-10 |
| KEGG_T_CELL_RECEPTOR_SIGNALING_PATHWAY | -0.661474723 | -2.373526205 | 1.00E-10 | 7.43E-10 |
| KEGG_CELL_ADHESION_MOLECULES_CAMS | -0.625576081 | -2.294534692 | 1.00E-10 | 7.43E-10 |
| KEGG_HEMATOPOIETIC_CELL_LINEAGE | -0.67019628 | -2.304672682 | 6.67E-10 | 4.66E-09 |
| KEGG_CYTOSOLIC_DNA_SENSING_PATHWAY | -0.76266433 | -2.413266778 | 7.86E-10 | 5.19E-09 |
| KEGG_B_CELL_RECEPTOR_SIGNALING_PATHWAY | -0.6755742 | -2.296746985 | 3.40E-09 | 2.13E-08 |
| KEGG_JAK_STAT_SIGNALING_PATHWAY | -0.550188683 | -2.071576086 | 7.92E-09 | 4.71E-08 |
| KEGG_NOD_LIKE_RECEPTOR_SIGNALING_PATHWAY | -0.706179857 | -2.305922158 | 9.64E-09 | 5.46E-08 |
| KEGG_VIRAL_MYOCARDITIS | -0.668652325 | -2.221308937 | 1.00E-07 | 5.18E-07 |
| KEGG_DRUG_METABOLISM_CYTOCHROME_P450 | 0.674522152 | 2.210616371 | 9.84E-08 | 5.18E-07 |
| KEGG_LEUKOCYTE_TRANSENDOTHELIAL_MIGRATION | -0.576555034 | -2.073660718 | 1.44E-07 | 7.12E-07 |
| KEGG_LYSOSOME | -0.558539247 | -2.034178745 | 2.37E-07 | 1.13E-06 |
| KEGG_FC_EPSILON_RI_SIGNALING_PATHWAY | -0.633709466 | -2.160024593 | 5.09E-07 | 2.33E-06 |
| KEGG_FC_GAMMA_R_MEDIATED_PHAGOCYTOSIS | -0.576744556 | -1.988360517 | 2.51E-06 | 1.11E-05 |
| KEGG_PROTEASOME | -0.712864745 | -2.132967722 | 2.96E-06 | 1.26E-05 |
| KEGG_RIG_I_LIKE_RECEPTOR_SIGNALING_PATHWAY | -0.598732349 | -1.992961036 | 6.78E-06 | 2.78E-05 |
| KEGG_METABOLISM_OF_XENOBIOTICS_BY_CYTOCHROME_P450 | 0.602860295 | 1.945414749 | 3.13E-05 | 0.000124227 |
| KEGG_APOPTOSIS | -0.532892499 | -1.837453961 | 4.31E-05 | 0.000165437 |
| KEGG_DNA_REPLICATION | -0.668592701 | -1.983691879 | 6.60E-05 | 0.00024532 |
| KEGG_VALINE_LEUCINE_AND_ISOLEUCINE_DEGRADATION | 0.631837894 | 1.952151255 | 7.57E-05 | 0.000272757 |
| KEGG_BUTANOATE_METABOLISM | 0.66358175 | 1.933948158 | 0.000134481 | 0.000470476 |
| KEGG_TIGHT_JUNCTION | 0.474195014 | 1.750565401 | 0.000152386 | 0.000517885 |
| KEGG_CELL_CYCLE | -0.459598749 | -1.690255203 | 0.000226065 | 0.00074694 |
| KEGG_VASCULAR_SMOOTH_MUSCLE_CONTRACTION | 0.476098343 | 1.705241029 | 0.000376004 | 0.001208777 |
| KEGG_PROPANOATE_METABOLISM | 0.652681741 | 1.885367393 | 0.000583764 | 0.001827294 |
| KEGG_WNT_SIGNALING_PATHWAY | 0.427811558 | 1.612064417 | 0.000606724 | 0.001850466 |
| KEGG_PPAR_SIGNALING_PATHWAY | 0.506642012 | 1.711015703 | 0.001209069 | 0.003595388 |
| KEGG_FATTY_ACID_METABOLISM | 0.579240786 | 1.768890225 | 0.001477857 | 0.004235533 |
| KEGG_TYROSINE_METABOLISM | 0.579158763 | 1.768639743 | 0.001495555 | 0.004235533 |
| KEGG_ENDOCYTOSIS | -0.382101551 | -1.459227334 | 0.002984311 | 0.008255254 |
| KEGG_ARRHYTHMOGENIC_RIGHT_VENTRICULAR_CARDIOMYOPATHY_ARVC | 0.494659476 | 1.686270257 | 0.003306879 | 0.008939649 |
| KEGG_MELANOGENESIS | 0.436848286 | 1.561678557 | 0.004024041 | 0.010636648 |
| KEGG_PRION_DISEASES | -0.576360033 | -1.693291869 | 0.004328325 | 0.011192237 |
| KEGG_MAPK_SIGNALING_PATHWAY | -0.339082438 | -1.360508227 | 0.00480522 | 0.012161026 |
| KEGG_P53_SIGNALING_PATHWAY | -0.481747476 | -1.600398193 | 0.006638377 | 0.016027355 |
| KEGG_COMPLEMENT_AND_COAGULATION_CASCADES | -0.465472177 | -1.560654693 | 0.006737162 | 0.016027355 |
| KEGG_PATHWAYS_IN_CANCER | -0.3237076 | -1.337290278 | 0.006644312 | 0.016027355 |
| KEGG_RETINOL_METABOLISM | 0.533272075 | 1.663911689 | 0.009250766 | 0.021575575 |
| KEGG_PATHOGENIC_ESCHERICHIA_COLI_INFECTION | -0.491650728 | -1.565336609 | 0.009838417 | 0.022091471 |
| KEGG_GAP_JUNCTION | 0.426834809 | 1.491184188 | 0.009843412 | 0.022091471 |
| KEGG_ADHERENS_JUNCTION | 0.44750218 | 1.511290497 | 0.010805252 | 0.023801043 |
| KEGG_PROTEIN_EXPORT | 0.604466216 | 1.620751219 | 0.01124523 | 0.024319828 |
| KEGG_HISTIDINE_METABOLISM | 0.572337983 | 1.612758606 | 0.011756868 | 0.024972294 |
| KEGG_O_GLYCAN_BIOSYNTHESIS | 0.566621202 | 1.596649614 | 0.013776429 | 0.028748596 |
| KEGG_PROXIMAL_TUBULE_BICARBONATE_RECLAMATION | 0.592288409 | 1.588098947 | 0.015310833 | 0.031399713 |
| KEGG_AMYOTROPHIC_LATERAL_SCLEROSIS_ALS | -0.470905114 | -1.499562524 | 0.01893576 | 0.038175574 |
| KEGG_PHENYLALANINE_METABOLISM | 0.626107901 | 1.570419713 | 0.019366241 | 0.038392723 |
| KEGG_ARGININE_AND_PROLINE_METABOLISM | 0.481365446 | 1.516090747 | 0.020427776 | 0.03919081 |
| KEGG_REGULATION_OF_ACTIN_CYTOSKELETON | -0.331888783 | -1.294018741 | 0.020355969 | 0.03919081 |
| KEGG_BETA_ALANINE_METABOLISM | 0.573786439 | 1.538489738 | 0.022601522 | 0.042672881 |
| KEGG_BASE_EXCISION_REPAIR | -0.521478748 | -1.506806975 | 0.024528303 | 0.045587143 |
| KEGG_GLYCINE_SERINE_AND_THREONINE_METABOLISM | 0.524973842 | 1.516464307 | 0.026567157 | 0.048616822 |
| KEGG_NEUROTROPHIN_SIGNALING_PATHWAY | -0.363788434 | -1.337895924 | 0.02724236 | 0.049097076 |
| KEGG_DILATED_CARDIOMYOPATHY | 0.398079614 | 1.403558625 | 0.029780389 | 0.052870132 |
| KEGG_VASOPRESSIN_REGULATED_WATER_REABSORPTION | 0.484681209 | 1.495999732 | 0.036994792 | 0.063774393 |
| KEGG_COLORECTAL_CANCER | -0.425321165 | -1.399331692 | 0.03681336 | 0.063774393 |
| KEGG_TYPE_II_DIABETES_MELLITUS | -0.466370928 | -1.435178865 | 0.044512512 | 0.075637802 |
| KEGG_LONG_TERM_POTENTIATION | 0.414349205 | 1.396792553 | 0.046507878 | 0.077915348 |

# Appendix 7

**Immune Correlation Analysis**

**Table 7. Immune Correlation Analysis.**

| Gene | Cell | cor | pvalue |
| --- | --- | --- | --- |
| GPR146 | B cells naive | -0.330388285 | 2.30E-07 |
| GPR146 | B cells memory | 0.242141174 | 0.000184004 |
| GPR146 | Plasma cells | 0.287733537 | 7.72E-06 |
| GPR146 | T cells CD8 | 0.26599483 | 3.76E-05 |
| GPR146 | T cells CD4 naive | -0.201786523 | 0.001920985 |
| GPR146 | T cells CD4 memory resting | -0.049160095 | 0.454200542 |
| GPR146 | T cells CD4 memory activated | -0.317035326 | 7.34E-07 |
| GPR146 | T cells follicular helper | 0.023848149 | 0.716676155 |
| GPR146 | T cells regulatory (Tregs) | 0.083952763 | 0.200681148 |
| GPR146 | T cells gamma delta | -0.175390042 | 0.007156694 |
| GPR146 | NK cells resting | -0.071637291 | 0.275108193 |
| GPR146 | NK cells activated | 0.268370977 | 3.18E-05 |
| GPR146 | Monocytes | 0.242402133 | 0.000180986 |
| GPR146 | Macrophages M0 | -0.477756658 | 9.56E-15 |
| GPR146 | Macrophages M1 | -0.295059413 | 4.39E-06 |
| GPR146 | Macrophages M2 | 0.259597609 | 5.85E-05 |
| GPR146 | Dendritic cells resting | 0.126149949 | 0.053967807 |
| GPR146 | Dendritic cells activated | 0.007565641 | 0.908354801 |
| GPR146 | Mast cells resting | 0.411741794 | 5.46E-11 |
| GPR146 | Mast cells activated | -0.246198477 | 0.000141991 |
| GPR146 | Eosinophils | -0.085286306 | 0.19359647 |
| GPR146 | Neutrophils | 0.056357566 | 0.390798236 |

# Appendix 8

**MiRNA and LncRNA**

**Table 8a. Gene-miRNA**.

| Gene | miRNA | miRanda | miRDB | TargetScan | Sum |
| --- | --- | --- | --- | --- | --- |
| GPR146 | hsa-miR-4269 | 1 | 1 | 1 | 3 |
| GPR146 | hsa-miR-1237-3p | 1 | 1 | 1 | 3 |
| GPR146 | hsa-miR-149-5p | 1 | 1 | 1 | 3 |
| GPR146 | hsa-miR-558 | 1 | 1 | 1 | 3 |
| GPR146 | hsa-miR-892b | 1 | 1 | 1 | 3 |
| GPR146 | hsa-miR-3175 | 1 | 1 | 1 | 3 |
| GPR146 | hsa-miR-1299 | 1 | 1 | 1 | 3 |
| GPR146 | hsa-miR-939-5p | 1 | 1 | 1 | 3 |
| GPR146 | hsa-miR-4270 | 1 | 1 | 1 | 3 |

**Table 8b. Gene-miRNA**.

| miRNA | lncRNA |
| --- | --- |
| hsa-miR-149-5p | LINC01043 |
| hsa-miR-939-5p | AATBC |
| hsa-miR-1237-3p | GS1-519E5.1 |
| hsa-miR-939-5p | RP11-627G23.1 |
| hsa-miR-149-5p | RP11-394A14.2 |
| hsa-miR-558 | RP11-458F8.4 |
| hsa-miR-939-5p | HP09025 |
| hsa-miR-558 | RP11-384K6.6 |
| hsa-miR-558 | RP5-991B18.1 |
| hsa-miR-939-5p | LINC00173 |
| hsa-miR-558 | LINC00265 |
| hsa-miR-1237-3p | AC091153.4 |
| hsa-miR-1237-3p | RP11-90K6.1 |
| hsa-miR-939-5p | VPS9D1-AS1 |
| hsa-miR-939-5p | CTA-941F9.10 |
| hsa-miR-939-5p | RP11-311F12.1 |
| hsa-miR-939-5p | RP11-278A23.4 |
| hsa-miR-558 | CITF22-24E5.1 |
| hsa-miR-558 | RP11-503N18.1 |
| hsa-miR-939-5p | RP5-1171I10.5 |
| hsa-miR-558 | RP11-638I8.1 |
| hsa-miR-939-5p | AP001469.9 |
| hsa-miR-149-5p | CTD-2008P7.3 |
| hsa-miR-939-5p | RP11-186N15.3 |
| hsa-miR-1237-3p | RP11-407A16.4 |
| hsa-miR-939-5p | LINC00599 |
| hsa-miR-1237-3p | CTC-548K16.6 |
